# Mapping global effects of the anti-sigma factor MucA in *Pseudomonas fluorescens* SBW25 through genome-scale metabolic modeling

**DOI:** 10.1186/1752-0509-7-19

**Published:** 2013-03-11

**Authors:** Sven EF Borgos, Sergio Bordel, Håvard Sletta, Helga Ertesvåg, Øyvind Jakobsen, Per Bruheim, Trond E Ellingsen, Jens Nielsen, Svein Valla

**Affiliations:** 1 Department of Biotechnology, Norwegian University of Science and Technology, Trondheim, N 7491, Norway; 2 Department of Biotechnology, SINTEF Materials and Chemistry, Sem Sælands v. 2A, Trondheim, N 7465, Norway; 3 Department of Chemical and Biological Engineering, Chalmers University of Technology, Kemivägen 10, Gothenburg, SE 412 96, Sweden

**Keywords:** *Pseudomonas*, Polysaccharide, Alginate, Genome-scale metabolic modeling

## Abstract

**Background:**

Alginate is an industrially important polysaccharide, currently produced commercially by harvesting of marine brown sea-weeds. The polymer is also synthesized as an exo-polysaccharide by bacteria belonging to the genera *Pseudomonas* and *Azotobacter*, and these organisms may represent an alternative alginate source in the future. The current work describes an attempt to rationally develop a biological system tuned for very high levels of alginate production, based on a fundamental understanding of the system through metabolic modeling supported by transcriptomics studies and carefully controlled fermentations.

**Results:**

Alginate biosynthesis in *Pseudomonas fluorescens* was studied in a genomics perspective, using an alginate over-producing strain carrying a mutation in the anti-sigma factor gene *mucA*. Cells were cultivated in chemostats under nitrogen limitation on fructose or glycerol as carbon sources, and cell mass, growth rate, sugar uptake, alginate and CO_2_ production were monitored. In addition a genome scale metabolic model was constructed and samples were collected for transcriptome analyses. The analyses show that polymer production operates in a close to optimal way with respect to stoichiometric utilization of the carbon source and that the cells increase the uptake of carbon source to compensate for the additional needs following from alginate synthesis. The transcriptome studies show that in the presence of the *mucA* mutation, the *alg* operon is upregulated together with genes involved in energy generation, genes on both sides of the succinate node of the TCA cycle and genes encoding ribosomal and other translation-related proteins. Strains expressing a functional MucA protein (no alginate production) synthesize cellular biomass in an inefficient way, apparently due to a cycle that involves oxidation of NADPH without ATP production. The results of this study indicate that the most efficient way of using a *mucA* mutant as a cell factory for alginate production would be to use non-growing conditions and nitrogen deprivation.

**Conclusions:**

The insights gained in this study should be very useful for a future efficient production of microbial alginates.

## Background

*Pseudomonas* is a genus of Gram-negative bacteria with features that have made them interesting to a wide community of researchers for a long time. *Pseudomonas* spp. range from important human pathogens (*P. aeruginosa*), via biocontrol agents and plant commensals useful in agriculture (*P. fluorescens)* to one of the most widely used “cell factories” for production of high-value-added products in industrial biotechnology (*P. putida*), and the genus is characterized by a great metabolic versatility.

Several species of *Pseudomonas* are also notable and well-studied in their capability to form biofilms [1], aggregates of cells that adhere to each other and to surfaces, embedded in an extracellular polymeric matrix. Formation of such biofilms can have serious clinical consequences, as seen in infections by the opportunistic human pathogen *P. aeruginosa*. Patients that are immunocompromised, including the general aging population, are susceptible to this pathogen both topically and systemically, and among cystic fibrosis patients, lung infections by *P. aeruginosa* are prevalent and have a major impact on morbidity and mortality [2,3].

One striking feature that is present in the majority of *P. aeruginosa* infections of the CF lung, is the so-called mucoid conversion of the pathogen, yielding a phenotype that produces large amounts of the exopolysaccharide alginate. This phenotype correlates with the ability of *P. aeruginosa* to persist in the lungs of CF patients [4] and is a general marker of poor survival for these patients [5]. The mucoid conversion typically takes place through inactivating mutations arising in the regulatory gene *mucA*[6], or proteolytic degradation of the encoded MucA protein [7]. MucA acts as an anti-sigma factor, binding and sequestering the alternative sigma factor σ^22^, encoded by the *algU* gene, that is essential for alginate production [8]. σ^22^ sigma factors are members of the ECF (extra cytoplasmic function) family of transcription factors that are known to respond to membrane stresses, and a recent microarray analysis [9] found that AlgU is a global stress response sigma factor, inducing several systems apart from alginate biosynthesis. This study also identified gene subsets encoding virulence factors specifically induced on conversion to mucoidy, including HCN biosynthesis. The metabolic features controlled by the AlgU-MucA system are not well studied, but a very recent metabolic footprinting study concluded that MucA modulates osmotic stress tolerance [10].

Alginate is a linear copolymer of mannuronic acid and its C-5 epimer guluronic acid, and bacterial alginate is only known to be produced by two genera, *Pseudomonas* and *Azotobacter*. An important industrial polymer, alginate is produced in bulk from seaweeds, but bacterial alginates have attracted significant interest over the last years due to the presence of alginate modifying enzymes, *i.e.* epimerases [11], in the producing organisms. These enzymes, taken together with the possibility to produce very homogeneous alginate from liquid bacterial cultivations, allow precise manipulation of the relative content and sequence of mannuronic and guluronic acid residues in the alginate, which influences the physico-chemical and immunological properties of the purified polymer significantly [12]. The genes encoding the alginate biosynthetic machinery are highly similar in the producing species, and are in *Pseudomonas* spp. organized in a 12-gene operon (*algD–8–44–K–E–G–X–L–I–J–F–*A) under the *algD* promoter – the only exception being the *algC* gene, encoding a phosphomannomutase, that is localized outside of the operon and transcribed from its own promoter. The alginate biosynthetic pathway (for reviews, see *e.g.*[13,14]) originates from fructose-6-phosphate and proceeds via the activated monomer GDP-mannuronic acid, to concomitant polymerization and export to the extracellular space. During the polymerization/export, mannuronic acid residues can be epimerized to guluronic acid, and can be *O*-acetylated in the O-2 and/or the O-3 position. One molecule of GTP and two molecules of NAD^+^ are consumed per monomer unit incorporated (counting from fructose-6-phosphate), whereas the degree of acetyl group incorporation depends strongly on the producing species [15]. *P. fluorescens* SBW25 is an organism well suited for industrial production of alginate as compared to either *P. aeruginosa* or *A. vinelandii*, because of both its non-pathogenicity and the relative simplicity of cultivation on an industrial scale.

Genome-scale metabolic reconstruction has become a well-established method for analysis of microbial metabolism, with numerous applications [16,17]. Although not directed towards dynamic modeling by nature, the steady-state assumption that underlies a stoichiometric metabolic reconstruction is well approximated in continuous liquid culture. Based on the annotated genome, the full spectrum of available metabolic pathways in the organism is reconstructed, and coupled with isotope labeling experiments, the actual flux distribution between these pathways can be assessed [18]. Alternatively, global data sets, most commonly transcriptomics data, can be used to identify highly regulated parts of the metabolic network [19], even if the metabolic reconstruction does not contain representations of regulatory mechanisms *per se*. This, in combination with computer simulations of gene knock-outs or knock-ins in the stoichiometric model, can identify attractive targets for metabolic engineering in the organism [20].

In this study, we have investigated the plant commensal *P. fluorescens* SBW25 by genome-scale modeling complemented by chemostat cultivations and microarray analysis of gene expression. The recently published genome sequence of SBW25 [21] (accession number [EMBL:AM181176]) shows that it contains all genes necessary for alginate biosynthesis, and from previous work, we have demonstrated the potential for high levels of alginate production in *P. fluorescens*[22]. Through genome-scale metabolic modeling, we aim to identify genetic and metabolic features that are crucial for alginate biosynthesis. The close coupling of alginate biosynthesis to central carbon metabolism necessitates a systems biology approach, and the results gained from the investigation of this non-pathogenic alginate producer could help to identify both novel antimicrobial targets for human medicine and key pathways features for optimized production of an industrially important biopolymer. Furthermore, we could by using the genome-scale model for performing integrative analysis identify the global role of the anti-sigma factor MucA on metabolism. The *P. fluorescens* metabolic reconstruction complements existing models of *P. aeruginosa*[23] and *P. putida*[24,25] and allowed us to perform a comparative metabolic analyses of these three important pseudomonads.

## Results and discussion

### Strain constructions

*P. fluorescens* wild-type strains have not been found to produce alginate under laboratory conditions, whereas it is known that *mucA* deficient strains of *P. aeruginosa* do(see *e.g.*[26]). An SBW25 *mucA* strain was constructed by introducing a point mutation leading to a truncated version of MucA in which the 37 C-terminal amino acids are absent. The *algC* gene is the only one located outside the *alg* operon that encodes an alginate biosynthetic enzyme. Whereas expression of the *alg* operon genes is known to be regulated by MucA, this is not the case for *algC* in *P. fluorescens* (unpublished results).

As the absence of a functional MucA protein in the cell is expected to have pleiotropic effects beyond alginate biosynthesis [9], it was necessary to construct control strains in order to discern between effects caused by alginate production, by over-producing the alginate biosynthetic enzymes, and by the more general pleiotropic effects of a defective anti-sigma factor MucA. In addition to the wild-type and the alginate-producing *mucA* strain, three control strains were constructed as described in Additional file 1. Relevant phenotypical features of the strains used in this study are summarized in Table 1. Firstly, the in-frame deletion inactivation of *algC*, i.e. a Δ*algC* strain, encoding wild-type MucA would allow us to observe potential roles of AlgC outside the alginate biosynthetic pathway, including consequences of potential effects on LPS [27] and rhamnolipid [28] biosynthesis. Secondly, a *mucA* Δ*algC* strain would not produce alginate, but would show pleiotropic effects following from *mucA* inactivation and, importantly, the cellular effect of overproducing the remaining alginate biosynthetic proteins. Finally, strain *mucA* TT*algD* was constructed that does not produce alginate, as a transcription terminator (TT) is inserted between *P*_*algD*_ and the translation start of *algD*. This strain should still exhibit the pleiotropic effects of *mucA* inactivation, although with minimal alginate-related effects as neither alginate nor the alginate biosynthetic proteins (except AlgC) should be produced. It needs mentioning, however, that very recently, Paletta and Ohman [29] found two putative promoters internal to the *alg* operon in *P. aeruginosa*, upstream of the alginate epimerse gene *algG* and the alginate acetylation gene *algI*, respectively. This could indicate the possibility of differential regulation within the operon to alter polymer structure under varying conditions.

**Table 1:** Overview of alginate-related phenotypes in the strains used in this study

**Strain**	***mucA***	***algC***	***alg *operon proteins produced**	**Alginate production**
Wild-type	+	+	-	-
Δ*algC*	+	-	-	-
*mucA*	-	+	+	+
*mucA* Δ*algC*	-	-	+	-
*mucA* TT*algD*	-	+	-	-

### Chemostat cultivations

Chemostat cultivation, allowing precise control of the cellular specific growth rate μ, ensures maximal homogeneity of the sampled cellular population both in the spatial and temporal dimensions (steady-state conditions) provided genetic stability.

A preliminary screening of carbon sources in *P. fluorescens* fermentations indicated that fructose and glycerol supported a significantly higher alginate yield per mmolC than did either glucose, sucrose, galactose, lactose or lactate. As both the uptake mechanism and point of entry into metabolism is different for fructose and glycerol, both carbon sources were selected for cultivations. Fructose is imported by the fructose-specific PTS FruAB-system [30], yielding fructose-1-phosphate, whereas glycerol is taken up by GlpF-facilitated diffusion and concomitant phosphorylation to glycerol-3-phosphate upon cytoplasmic entry [31]. For biosynthesis of alginate, fructose-1,6-bisphosphate is then the common precursor from both fructose and glycerol, from fructose by direct phosphorylation of fructose-1-phosphate, and from glycerol-3-phosphate via triose phosphates. Fructose-6-phosphate is then generated from fructose-1,6-bisphosphate by fructose bisphosphatase, and is the immediate substrate for the alginate biosynthetic enzymes by first converting fructose-6-phosphate to mannose-6-phosphate. Note that similarly to other pseudomonads, SBW25 does not contain a phosphofructokinase, thus lacking the possibility to phosphorylate fructose-6-phosphate.

In addition to the SBW25 wild-type (WT) strain, two genetically engineered strains were grown on both carbon sources in order to elucidate the impact of alginate biosynthesis on cellular metabolism. As described above, the *mucA* strain has the full mucoid phenotype, whereas the *mucA* TT*algD* strain has a second mutation that abolishes expression of the *alg* operon biosynthetic genes. In the fructose cultivations, the two additional control strains *ΔalgC* and *mucA* Δ*algC* were included to better understand the effects of MucA outside alginate biosynthesis as discussed above.

Chemostat cultivations were performed as described in Materials and Methods, with fructose or glycerol as the sole carbon source (both 40 g/l), under nitrogen limitation of growth. Measured variables were biomass concentration, residual carbon source in the medium, alginate production and CO_2_ evolution (respiration). In a chemostat, specific growth rate is fixed and based on this, specific rates of carbon source uptake, carbon dioxide production and alginate production were calculated, and the data are summarized in Table 2. Carbon balance calculation, as listed in the rightmost column, shows that all carbon taken up can be accounted for to within 95% in all conditions tested.

**Table 2:** Physiological characteristics of the strains and conditions studied

***Carbon source***	***Strain***	***OD***_***660***_	***C source Uptake [mmolC/ gDW h]***	***Alginate production, deacetylated [mmolC/gDW h]***	***Acetate (acetyl from alginate) [mmolC/gDW h]***	***CO***_***2 ***_***excretion [mmolC/ gDW h]***	***C in biomass [mmolC/ gDW h]***	***C balance [%]***
Fructose	Wild-type	7,8	9,4	-	-	7,5	1,51	95,7
Fructose	*mucA*	7,0	18,3	12,3	0,77	4,6	1,51	104,4
Fructose	Δ*algC*	8,1	9,1	-	-	7,4	1,51	97,6
Fructose	*mucA* Δ*algC*	8,4	5,8	-	-	4,3	1,51	99,3
Fructose	*mucA* TT*algD*	9,2	5,6	-	-	3,9	1,51	96,9
Glycerol	SBW25	9,2	8,3	-	-	6,4	1,51	96,3
Glycerol	*mucA*	7,4	17,5	10,6	1,22	3,6	1,51	96,8
Glycerol	*mucA* TT*algD*	9,9	6,9	-	-	5,0	1,51	95,4

Measured and calculated physiological parameters for chemostat cultivations (D = 0.04) of *P. fluorescens* SBW25 (wild-type) and derived mutant strains. The value for mmolC/gDW h in biomass is calculated from the specific growth rate used in the experiments (μ = 0.04 hr^-1^) and the biomass stoichiometric composition assumed in the genome scale model. The % value for carbon balance represents the sum of carbon accounted for in alginate, CO_2_ and biomass as a percentage of the carbon taken up by the cells.

Bacterial alginates are O-acetylated to varying degrees [15]. The degree of acetylation was determined by HPLC measurements of acetate released from the alginate by hydrolysis to be 0.19 and 0.32 on fructose and glycerol, respectively. No free acetate was detected in the culture growth media.

It is apparent from Table 2 that the *mucA* inactivation has a profound effect on respiration in *P. fluorescens* SWB25. Whereas the measured differences between the WT strain and the Δ*algC* mutant on fructose are negligible, the *mucA* Δ*algC* mutant, which also does not produce alginate, has a significantly lower respiration, and hence a lower fructose uptake. This is even more pronounced in the *mucA* TT*algD* strain, which makes neither alginate nor the dedicated alginate biosynthetic proteins. In the *mucA* strain, respiration is still low, whereas the fructose uptake is strongly elevated to accommodate the carbon drain into alginate. On glycerol, the reduction in respiration going from the WT strain to the *mucA* mutant reflects the case on fructose, whereas the *mucA* TT*algD* strain on glycerol has a level of respiration that is intermediate to those of the WT and *mucA* strains, as opposed to the fructose case. This is an early indication that metabolism of the two carbon sources is regulated differently in *P. fluorescens* SBW25.

### Transcriptome analyses

Based on the annotated *P. fluorescens* SBW25 genome sequence, a custom expression microarray was constructed for transcriptome analysis. This array covers 6,519 genes, including 478 genes from the environmental plasmid pQBR103. The apparent discrepancy with the 6,009 chromosomal genes published by Silby *et al.*[21] is due to the inclusion of some putative pseudogenes in the microarray. Note that the pQBR103 plasmid included in the published genome sequence was shown to be acquired by the organism during a field release experiment [32], and is not present in the originally isolated SBW25 strain used in this study; it was included in the microarray design for versatility in potentially comparative studies.

**Figure 1:**
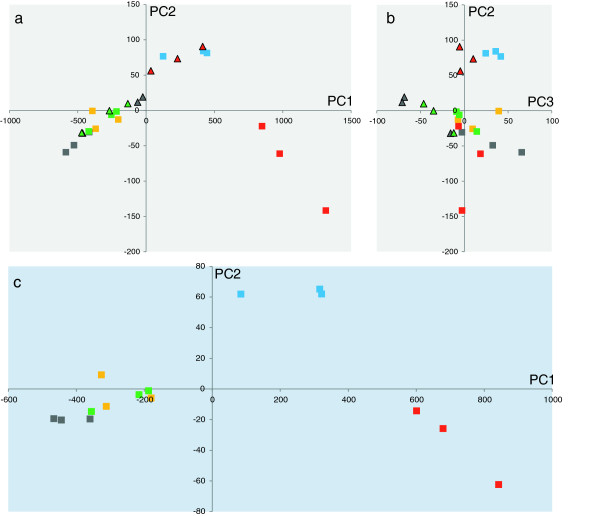
**PCA plots of microarray data from chemostat cultivations.***Panel ****1a****):* All strain–carbon source datapoints, PC1 (principal component 1, x axis, 85.1% explained variation) vs. PC2 (y axis, 9.3% explained variation). *Panel ****1b****):* All strain–carbon source datapoints, PC3 (x axis, 5.5% explained variation) vs. PC2 (y axis, 9.3% explained variation). Panel **1a** and **1b** will combine to show all three dimension in three first PCs. *Panel ****1c****):* All datapoints from fructose-grown cultivations, PC1 (90.1% explained variation) vs. PC2 (7.4% explained variation). Squares, carbon source fructose; triangles, carbon source glycerol. Strains: Grey, WT; yellow, Δ*algC*; red, *mucA*; blue, *mucA* Δ*algC*; green, *mucA* TT*algD*. All datapoints are independent fermentations, *i.e.* all biological conditions are present in triplicate data points. Genes used to construct the PCA are selected by ANOVA with p-values < 0.005.

RNA samples were isolated from triplicate, independent chemostat cultivations as described above. The complete transcriptome data set is available in Additional file 2, both as raw and quantile normalized data. Process variability in RNA isolation, preparation and analysis was investigated (data not shown), confirming that the majority of the observed variability between replicates does indeed represent biological differences between cultivations. Principal component analysis (PCA) was performed on the transcriptome data described here, and the results are shown in Figure 1. It confirms that there is good reproducibility within replicate sample sets. There is a clear PCA separation of the strains into three distinct groups, along the two first principal components. (Figure 1a,c). The *mucA* and *mucA* Δ*algC* strains grown on fructose are separated from each other, and from the remaining three strains which cluster closely together. The *mucA* Δ*algC* is phenotypically distinct in that it does not produce alginate, but the alginate biosynthesis genes are transcribed and translated. The anti-sigma factor MucA is effectively a global regulator through the MucA–AlgU system, and a strong effect on cellular processes can be expected. Interestingly, the *mucA* TT*algD* strain, which does not make either alginate or most of the alginate biosynthetic enzymes, does not group with either the *mucA* or the *mucA* Δ*algC* strains, but rather with the WT. This could indicate that even the expression of only the alginate biosynthetic enzymes with no concomitant alginate biosynthesis (as is the case in the *mucA* Δ*algC* strain) imparts a significant physiological load on the cells. This is supported by the observation that when genes are clustered according to their expression pattern across all strains (data not shown), virtually all genes encoding ribosomal proteins show an expression profile similar to that of the *alg* operon (*i.e.* high relative expression in the *mucA* and *mucA* Δ*algC* strains). It should also be noted that in the PCA plots, the *mucA* strain on glycerol is clustered with the *mucA* Δ*algC* strain, rather than the *mucA* strain, on fructose (Figure 1a), although a separation between carbon sources can be observed along PC3 (Figure 1b). This somewhat counterintuitive clustering could be an indication that the *mucA* inactivation has different physiological effects depending on the carbon source utilized by the cells.

If only the subset of metabolic genes is considered and all mutant strains on fructose are compared against the WT, the indicative differences observed in the PCA plot are confirmed. Using 2-fold regulation and ANOVA p-value of < 0.005 as significance cut-off, no metabolic genes are changed in the Δ*algC* mutant, whereas in the *mucA* TT*algD* strain, only *algC* and the PFLU5987 gene are regulated above the significance cut-off, both up in the mutant strain. The latter gene encodes an acetylglutamate kinase ArgB (EC 2.7.2.9). However, this gene is localized immediately downstream of *algC* on the chromosome, and they are most likely co-transcribed.

In the *mucA* strain, and to some extent in the *mucA* Δ*algC* strain, two functional sets of genes besides the alginate biosynthetic operon are recognized as significantly upregulated, in addition to the genes encoding ribosomal proteins and other translator proteins. The first category encompasses genes directly involved in energy generation, including the ATP synthase subunits, cytochrome C encoding genes and NADH dehydrogenase genes. This correlates very well with the recent findings by Lien *et al.*[33] on metabolome changes in *P. fluorescens* SBW25, where it is shown that the most significant metabolome changes are changes in the GXP pool related to alginate synthesis and changes in the AXP pool related to *mucA* inactivation. The second notable set of upregulated genes encode functions on both sides of the succinate node in the TCA cycle, *i.e.* the succinate dehydrogenase and succinyl-CoA synthetase complexes. In this context, it is noteworthy that succinate dehydrogenase is the only enzyme that catalyzes reactions in both the TCA cycle and the electron transport chain. Other metabolic genes upregulated above the 2-fold threshold in the *mucA* strain include the purine pathway genes *purF*, *purN*; carbonic anhydrase; isocitrate dehydrogenase; S-adenosylmethionine synthetase; PEP synthase and both isoenzymes of glucose-6-phosphate dehydrogenase.

**Figure 2:**
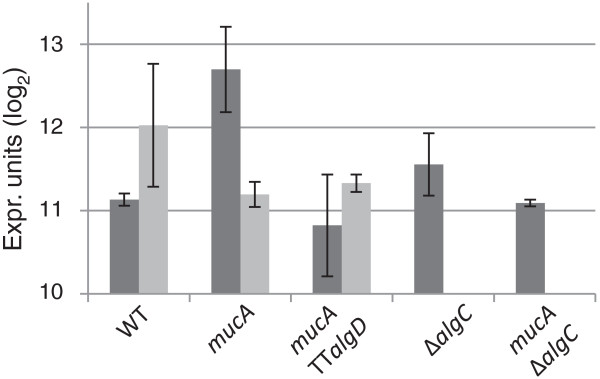
**Differential gene expression profiles in carbon source regulated operon.** Gene expression profiles (log_2_ normalized expression values) in all strain–carbon source combinations for gene PFLU3201, which is the first gene in the putative operon PFLU3201–PFLU3193. Dark grey, fructose as carbon source; light grey, glycerol as carbon source. Data points are averages of three independent cultivations; error bars equals one standard deviation.

The genes PFLU3193–PFLU3201 display a remarkable expression profile that is unique in the transcriptome data, where the *mucA* inactivation seems to have opposite effect depending on the carbon source. As these genes have the same direction on the chromosome and very short intergenic linkers, it seems probable that they are co-transcribed. The genes upstream and downstream, PFLU3192 and PFLU3202, do not share the same expression profile. Normalized gene expression profiles for the first gene in the putative operon is shown in Figure 2; the downstream genes show very similar expression profiles (data not shown), supporting the assignment of these genes to an operon structure. The expression level is upregulated approximately twofold on fructose compared to the wild-type, in the alginate producing *mucA* strain, whereas this ratio is inverted in the glycerol-grown cultures. Absolute expression values in the two cases also vary approx. twofold between carbon source conditions for the alginate producing strain, indicating that expression of these genes is regulated in a multifactorial way. All of the genes in this putative operon are indicated by BLAST sequence similarity to encode metabolic enzymes; PFLU3193 shows high similarity to 2-ketoacid dehydrogenase subunits, more specifically to pyruvate dehydrogenase subunit E1. For the remaining genes in the cluster, the exact reaction catalyzed is less clear, although there is a strong indication of an involvement in fatty acid biosynthesis or metabolism, as encoded by the presence of 3-ketoacyl-acyl carrier protein (ACP) synthases (PFLU3201 and PFLU3199), ACP (PFLU3200), acyl-CoA oxidase (PFLU3198) and a possible thioesterase (PFLU3197). Alternatively, these enzymatic activities could participate in biosynthesis of polyketides; other strains of *P. fluorescens* are known to produce such compounds, including mupirocin [34], 4-diacetylphloroglucinol [35] and pyoluterin [36]. In such a context, it is also interesting to note that for all the genes PFLU3196–PFLU3201, the BLAST hits with the highest similarity originate from members of the *Streptomyces* genus, known for their extensive polyketide biosynthetic capabilities.

### Genome-scale metabolic modelling

#### Metabolic reconstruction at the genome level

Reconstruction of an organism's complete metabolic network comprises the assignment of well-defined biochemical reactions to gene products, and the integration of these reactions into a set that allows for simulation of cellular growth, *i.e.* the conversion of growth substrates into biomass and by-products (*e.g.* CO_2_), and potentially the production of secondary metabolites like biopolymers. The stoichiometry and completeness of the reaction network is then validated by comparison of the simulated results with wet-lab experiments. It is worth noting that most genome-scale reconstructions up to date make the simplifying assumption that growth occur under steady-state conditions, so that the simulation can be run as purely stoichiometric on an elemental basis without time-dependent changes. Also, the effect of regulation, which is critical to any organism's adaptation to its environment, is usually ignored in the steady-state assumption, although some work is beginning to appear that integrates data on regulation with the metabolic network structure [37].

The manual reconstruction of a metabolic network continues to be a labour-intensive process that is mainly based on incorporation of knowledge published previously on the organism in question. Since the first genome-scale reconstructions were completed, the use of software and databases for aid in this process has gathered significant and increasing attention. Although the essential data is mostly interconvertible between the different model formats, the choice of reconstruction software will have some influence on the exact model building strategy, format and workflow. An attempt to automate the reconstruction process for bacteria is the SEED database [38], which maps genes through a web interface to a pre-constructed metabolic network, generating a draft model in SBML format that can in principle be used directly for simulations. Another software package for model reconstruction is the Pathway Tools suite [39,40], which is built around the BioCyc database collection of metabolic pathways encompassing virtually all domains of life; currently, 1129 genomes and their respective databases are contained in BioCyc. The comprehensiveness and degree of manual database curation, as well as the fact that Pathway Tools is still in active development almost a decade after it was launched, contributed strongly to the decision of making it a central tool in this work.

A more recent development in the quest for (semi-)automation of the reconstruction process is the RAVEN Toolbox software suite [Agren *et al.*, submitted] [41] that integrates both the reconstruction and subsequent flux simulations on the model. One significant advantage of the latter approach is the complete control that the user exerts on every sub-step of the model construction; the reconstructed network and the input parameters for simulation all reside in a spreadsheet format that constitutes a detailed, intuitive and easily accessible overview of all reactions, metabolites, genes and simulation parameters.

Here, a hybrid approach was pursued, combining the proven reconstruction power of the Pathway Tools software with the user accessibility and simulation power of the RAVEN Toolbox. Based on the annotated *P. fluorescens* SBW25 genome [21] we used Pathway Tools v12.0 to reconstruct a draft metabolic model, as described in Materials and Methods. As the model reconstructed in Pathway Tools was not formulated or formatted for simulation purposes, the model was restructured into RAVEN Toolbox format.

**Figure 3:**
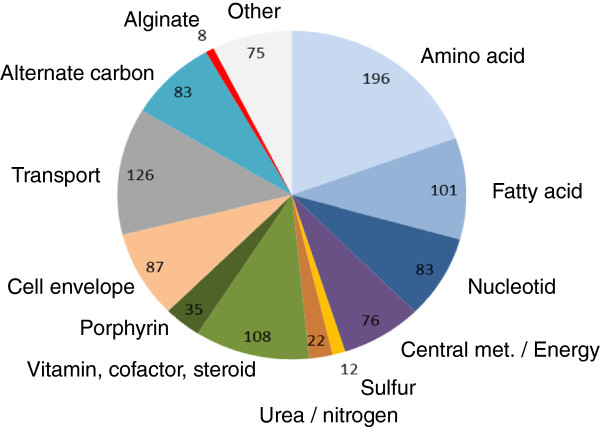
**Reaction classes present in the *i*SB1139 metabolic network.** Classification and distribution of all reactions in the *i*SB1139 genome-scale metabolic model of P. fluorescens SBW25 into functional groups of the overall metabolic network.

The final model contains 1194 metabolites participating in 1012 unique biochemical reactions, of which 126 are transport reactions. 1139 ORFs are associated to the reactions, and 367 of the proteins encoded participate in 112 protein complexes. The model is denoted *i*SB1139 by nomenclature convention [42], and the distribution of reactions in functional classes is shown in Figure 3.

#### Comparison between *Pseudomonas* genome-scale models

**Figure 4:**
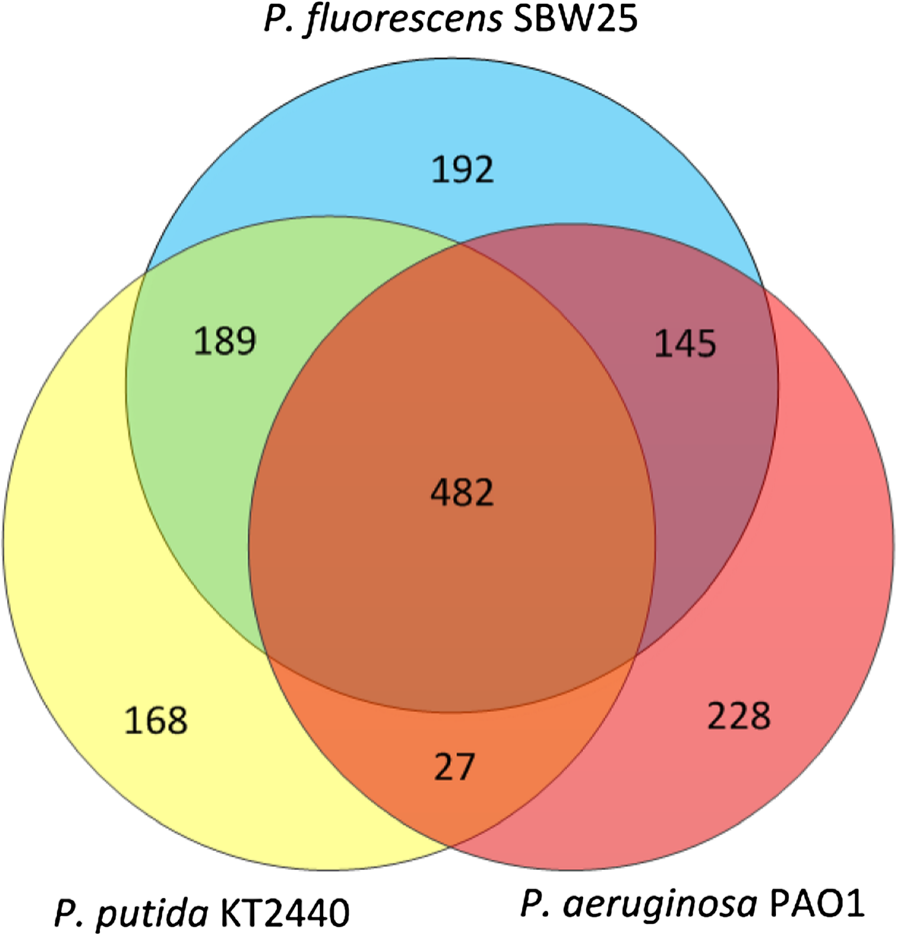
**Metabolic reaction overlap and uniqueness in published *Pseudomonas *models.** Venn diagram of reactions present in the *i*MO1056 (*P. aeruginosa* PAO1), *i*JN746 (*P. putida* KT2440) and *i*SB1139 (*P. fluorescens* SBW25) genome-scale metabolic models discussed here.

Genome-scale metabolic reconstructions of bacteria are conceptually similar, the differences being mainly the selection and number of entities (genes, reactions, metabolites) included in each reconstruction. We performed a reaction-by-reaction manual comparison of the three *Pseudomonas* genome-scale models considered here, *i*JN746 (*P. putida* KT2440) [25], *i*MO1056 (P*. aeruginosa* PAO1) [23] and *i*SB1139 (*P. fluorescens* SBW25, this work). The complete comparison in tabular format can be found in Additional file 3. Reactions in the *P. putida* model describing diffusion from periplasm to extracellular space were excluded, as were so-called exchange reactions in both the *P. putida* and *P. fluorescens* models. Such exchange reactions are added to facilitate flux simulations, but do not represent cellular processes. The complement of shared and unique reactions between the three *Pseudomonas* models is visualized as a Venn diagram in Figure 4. All three models have a shared core inventory of 482 reactions. The alginate biosynthetic genes, of special interest here, are conserved in the *P. aeruginosa* and *P. fluorescens* models, but are not included in the *P. putida* model, although the genes are present in the organism.

Among reactions unique to the *i*MO1056model of *P. aeruginosa*, two functional groups stand out in numbers; the first group is transport (46 reactions) with unique substrates or unique transport mechanisms on common substrates. The second group constitutes biosynthesis of virulence factors (44 reactions), including LPS/lipid A biosynthesis, quorum sensing biosynthetic pathways and phenazine biosynthesis, reflecting the pathogenic lifestyle of *P. aeruginosa* and its emphasis in the modeling process.

For the *i*JN746 *P. putida* model, degradation of aromatic compounds, including the beta-ketoadipate pathway, constitutes 56 reactions unique to this model, whereas the biosynthetic pathway for the industrially relevant polymer polyhydroxyalkanoate (PHA) contains 26 unique reactions. The existence of an alternative, 12-step lysine degradation pathway in *P. putida*, as described by Revelles *et al.*[43] is also noteworthy in the comparison of metabolic capabilities contained in the three models, as this pathway has been suggested [44] to replenish TCA cycle intermediates.

In the *P. fluorescens* model, unique reactions encompass a significant number concerning transport (33 reactions). Metabolism of alternate carbon compounds, either as complete pathways (*e.g.* metabolism of 4-hydroxyphenyl acetate) or as smaller sets of reactions, constitute a significant fraction of these unique reactions; some are introduced by the reconstruction software without being well connected to the metabolic network. They are, however, retained in the model so it can serve as a knowledge base for the organism and also for the future potential integration in the main metabolic network as improved annotation of metabolic genes becomes available. These detached reactions are not detrimental to the flux simulations as they are excluded from the working SBML model.

When assessing model completeness, 27 reactions are found to be part of both the *P. aeruginosa* and *P. putida* model, but not included in the *P. fluorescens* model described here. All of these, however, represent reactions with dead-end metabolites, reactions with alternative cofactors, transport reactions or reactions already described (above) as absent from the *P. fluorescens* SBW25 model. Thus, no significant metabolic 'holes' exist solely in the *P. fluorescens* model. Comparison of manually constructed genome-scale models is challenging, in that the reconstruction process involves a significant number of non-standardized decisions, notably in the association of metabolic reaction substrates, products and cofactors to genes and gene products. Oberhardt *et al*. [45] have very recently developed an elaborate process for reconciliation of genome-scale metabolic models, and demonstrated its application on the previously published models of *P. aeruginosa* PAO1 and *P. putida* KT2440. Although the described process was based on a manual reconciliation of the two models, the authors suggest a workflow that should be amenable to a degree of automation and might provide a first step on the path towards full multi-model comparisons on a metabolic network level.

#### Estimation of biomass composition and energetic parameters in the *i*SB1139 model

Biomass composition in the *i*SB1139 model was chosen to be identical to that used for the *P. putida i*JN746 model [25], in turn based very closely on measured values for *E. coli*[46,47]. For the second *P. putida* KT2440 model *i*JP815 [24], simulations indicated that using the experimentally verified biomass composition for *E. coli,* in the absence of experimental data for *P. putida,* was a sound approximation. Furthermore, Roels [48] found very similar elemental composition and ash content for *P. fluorescens* and *E. coli* under unlimited growth. The growth associated ATP consumption that we have used is an average between the values for *P. putida* and *P. aeruginosa* (both closely based on the value from *E. coli*), which is equal to 43 mmol of ATP per gram of biomass produced.

The annotated genome of *P. fluorescens* SBW25 indicated the presence in the electron transport chain of at least three terminal oxidases, cytochrome *c* oxidase, *bd* (ubiquinol) oxidase and *bo* (ubiquinol) oxidase, with different ATP-generating efficiencies. Such complementary systems are thought to be optimized for cellular energy generation under different dissolved oxygen tension in the cytoplasm [49,50].

While cytochrome c oxidase translocates 6 protons for each pair of electrons transferred to oxygen, *bd* and *bo* translocate only 2 protons. The overall theoretical P/O ratio (in absence of proton leakages across the membrane) can therefore vary between 2.5 and 1.5 depending on which terminal oxidase is active.

#### Theoretical and experimental biomass yields

The strains not producing alginate show different biomass yields on fructose (see Table 1). The wild type strain and the Δ*algC* mutant have biomass yields of 0.14 g-DW/g-fructose, while the double mutants *mucA* Δ*algC* and *mucA* TT*algD* show yields of 0.23 and 0.24 g-DW/g-fructose, respectively. As calculated from the model, the optimal biomass yields in fructose using P/O ratios of 2.5 and 1.5, respectively, are 0.72 and 0.58 g-DW/g-fructose. A typical bacterial non-growth associated ATP consumption of 1.5 mmol-ATP per g-DW per hour [51] would explain only a further drop of 0.06 g-DW/g-fructose. This means that the experimental biomass yield is far from optimal and the cells are using inefficient biomass synthesis pathways or are dissipating energy in futile cycles. In the next section it will be shown that the difference in biomass yield between the wild type strain and the Δ*algC* mutant with respect to the *mucA* Δ*algC* and *mucA* TT*algD* mutants can be explained by the activity of a cycle oxidizing NADPH without ATP production, which is consistent with a suboptimal metabolism in terms of energy utilization. This lack of optimality could be due to the difference in growth conditions between the chemostat situation (40 g/l of fructose or glycerol) and the native soil habitat of *P. fluorescens, e.g.* that the chemostat condition is one of relative carbon excess for which the metabolic network is not evolved optimally.

The same lack of optimality in the biomass yield can be observed when the cells grow using glycerol as a carbon source. The wild type and the *mucA* TT*algD* strains show biomass yields of 0.16 and 0.20 g-DW/g-glycerol, while the optimal yields for P/O ratios of 2.5 and 1.5 respectively are 0.81 and 0.65 g-DW/g-glycerol.

#### Theoretical and experimental alginate yields

In order to calculate the actual efficiency of alginate biosynthesis of the alginate producing *mucA* strain we have subtracted from the carbon source consumed the amount of carbon that is dedicated to biomass production in the non-alginate-producing strain with the highest biomass yield, *i.e.* the maximum amount of carbon *P. fluorescens* SBW25 can channel into biomass under any of the conditions tested here. Alginate yields calculated on the remaining carbon available will then be relative to carbon used for alginate biosynthesis. The resulting efficiencies of alginate biosynthesis are 0.99 mmol-alginate/mmol-fructose and 0.5 mmol-alginate/mmol-glycerol. The theoretical yields on fructose using P/O ratios of 2.5 and 1.5 respectively are 0.93 and 0.85 mmol-alginate/mmol-fructose, which means that under the above approximation of an upper threshold of biomass yield, the alginate biosynthesis is operating close to its optimal stoichiometric yield. The alginate biosynthesis from glycerol does not require the oxidative phosphorylation and has a theoretical yield of 0.45 mmol-alginate/mmol-glycerol, which is independent of the P/O ratio chosen, this is due to the fact that, when glycerol is supplied as the carbon source, the metabolic network is flexible enough to compensate the difference in respiratory ATP yield by using the reaction catalyzed by pyruvate kinase as an alternative ATP source. The results seem to indicate that the cell is able to transform both carbon sources into alginate in a close to optimal way even if their biomass production is far from optimal. Indeed, the fraction of imported carbon that goes into alginate is very high; 71% and 68% on fructose and glycerol, respectively. All the above would indicate that in order to use the *mucA* strain as an optimal alginate-producing cell factory it could be enough to use it in non-growing conditions such as for example under nitrogen deprivation.

### Random sampling

The functional association of the measured transcriptome data to actual metabolic fluxes is challenging, as it has been shown that there is no clear correlation between gene expression and protein concentration [52] or metabolic fluxes [53,54]. Recently, we developed a ‘convex basis random sampling’ method [55], which allows for a genome-scale statistical comparison of experimental gene expression changes and estimated metabolic fluxes. The advantage of this method is that it uses the observed changes (between two conditions) in a small set of experimentally measured fluxes such as respiration, carbon source uptake rate, secondary metabolite production etc. to infer the probabilities of flux change in each of the fluxes in the genome-scale metabolic network. This method facilitates the classification of enzymes as either transcriptionally regulated (*i.e.* showing significant correlation between flux change and gene expression change), metabolically regulated if change in the corresponding reaction is likely to be driven only by metabolite concentrations or post-transcriptionally regulated (*i.e.* where a significant expression change does not correlate with the estimated flux change). Each reaction in the network is assigned a score between 0 and 1, which indicates the likelihood for the reaction to be transcriptionally regulated. The reactions can be sorted by their corresponding scores in order to identify which transcriptional changes have a greater impact on the observed changes in the metabolic phenotype.

Here this method was applied to the genome-scale reconstruction of *P. fluorescens* SBW25. The experimental conditions for comparison in this case were the strain–carbon source combinations, whereas the measured variables were the microarray data sets and observed exchange fluxes in the chemostat cultivations, *i.e.* carbon source consumption and production of CO_2_ (respiration), alginate (including acetylation), and cell biomass. Note that specific growth rate was fixed at 0.04 hr^-1^ for all cultivations. Pairwise comparisons were done of the wild-type strain vs. all mutants on each carbon source.

#### Wild type versus *mucA* strain on fructose

In the alginate-producing *mucA* strain growing on fructose, unsurprisingly, the reactions involved in the alginate biosynthetic pathway, encoded by *algD, algG, algX, algI, algJ, algF, algA* and *algC*, appeared to be transcriptionally up-regulated with high evidence scores. The reaction PEP synthase (generating PEP for the fructose PTS transport system), which is associated in the model with the gene PFLU4620, shows a strong transcriptional up-regulation (with the 8th top ranking evidence score). This reaction is coupled to fructose transport and phosphorylation, which is consistent with the much higher specific fructose uptake rate in *mucA* compared with the wild type. The gene association that appears in the model is uncertain and the gene PFLU4620 is annotated as a *putative PEP synthase*. The fact that the flux in the reaction PEP synthase appears to be well correlated with the change in the transcription level of PFLU4620 indicates the gene association.

Interestingly, a highly significant transcriptional down-regulation of ribose-5-phosphate isomerase A (RpiA) is also found (with the 4th ranking evidence score). This enzyme is central in the pentose phosphate pathway. A down regulation of this pathway would mean that the cells produce less NADPH and more NADH.

The amount of energy required for alginate biosynthesis is significant; per mole of extracellular fructose incorporated in (extracellular) alginate, three moles of high-energy phosphate esters (PEP, ATP and GTP) are hydrolyzed, and two moles of NAD^+^ are reduced to NADH. From the random sampling analysis, pyruvate dehydrogenase cytochrome (PoxB), ubiquinol-cytochrome C reductase and cytochrome C oxidase appear as transcriptionally up-regulated reactions (6th, 9th and 10th evidence scores). The F_0_F_1_ ATP synthase appears ranked in the 15th position by evidence score. These alterations would support an increased ATP production rate in order to supply the necessary energy for alginate biosynthesis. As shown by Lien *et al.*[33], the most significant changes in the metabolome of SBW25 are alterations in the GXP and AXP pools upon biosynthesis of alginate and *mucA* inactivation, respectively. This is in good correlation with the results from the random sampling analysis.

#### Wild type versus Δ*algC* mutant in fructose

Comparison of the Δ*algC* mutant with the wild-type yielded only very small phenotypical changes and it was therefore not possible to detect any significantly perturbed fluxes using the random sampling algorithm.

#### Wild type versus the double mutants *mucA* Δ*algC* and *mucA* TT*algD* in fructose

For the double mutants *mucA* Δ*algC* and *mucA* TT*algD*, an interesting pattern occurs; the three highest ranked reactions identified as showing transcriptional regulation of their fluxes in the *mucA* Δ*algC* strain (all down-regulated) form an NADPH oxidizing cycle together with the 9th ranked reaction (also down-regulated), with the overall net stoichiometry NADPH + H^+^ + ½ O_2_ → H_2_O + NADP^+^. The reactions underlying this cycle are:

(1) *Aspartate aminotransferase* (AatB; PFLU3176)*:* L-glutamate + oxaloacetate → L-aspartate + alpha-ketoglutarate

(2) *Catalase* (KatB; PFLU5339)*:* H_2_O_2_ → H_2_O + ½ O_2_

(3) *NADP-specific glutamate dehydrogenase* (GdhA; PFLU5326) *:* NADPH + alpha-ketoglutarate + NH_4_^+^ → L-glutamate + NADP^+^ + H_2_O

(4) *L-aspartate oxidase* (NadB; PFLU1465)*:* L-aspartate + O_2_ + H_3_O^+^ → NH_4_^+^ + H_2_O_2_ + oxaloacetate

Strikingly, down-regulation of three of the above four reactions, specifically (2), (3) and (4) also rank within the top four in the *mucA* TT*algD* strain, giving a strong indication that the same metabolic mechanism is functioning in this strain too.

Cyclic operation of pathways in central metabolism is widespread in bacteria [56], although such pathways rarely imply a direct dissipation of energy or cellular reductive potential, as suggested for the cycle (1)–(4). There are, however, examples both in bacteria [57,58] and yeast [59] where overproduction of NADPH, primarily from increased flux through the pentose phosphate pathway, causes a metabolic imbalance that must be corrected by reoxidation of NADPH without concomitant anabolism. Soluble transhydrogenases, like UdhA in *E. coli*[60], are considered important for NAPDH balancing in some species through the equilibrium NADPH + NAD^+^ ↔ NADP^+^ + NADH. However, as *udhA* analogs are only found in Enterobacteria, other mechanisms for NADPH reoxidation must exist. Fuhrer and Sauer [61] investigated growth and NADPH balancing in eight bacterial species, including *P. fluorescens* strain 52–1C, on glucose. They found that for six of these species, NADPH biosynthesis exceeded anabolic demands, and four of these six species relied on other redox-balancing systems than transhydrogenase reactions. Such systems were suggested to include dual cofactor specificities of catabolic enzymes or isoenzymes with distinctive cofactor specificities, as well as the possible existence of redox cycles and NADH kinases. In the *P. fluorescens* strain, NADPH biosynthesis seemed balanced under glucose growth, and the absence of soluble transhydrogenase activity was experimentally verified. Interestingly, Chavarría *et al.*[62] very recently demonstrated regulation of the pyruvate shunt (malate – pyruvate – oxaloacetate) in *P. putida* KT2440 by the PtsN paralog of the FruB fructose-specific PTS protein. When PtsN was inactivated, either by gene knock-out or by mutagenesis of the phosphorylation site, the flux through the pyruvate shunt was increased when either glucose or fructose was used as carbon source. Moreover, flux through the shunt was 2.4-fold higher on fructose (a PTS substrate) than on glucose (a non-PTS substrate). This is of relevance for the results reported here since the pyruvate shunt constitutes a seemingly futile bypass of one reaction in the TCA cycle, as ATP is hydrolyzed in the shunt. All the relevant genes described in the *P. putida* case (*ptsP, ptsO, ptsN*) are present (and virtually identical at the amino acid level) in *P. fluorescens* SBW25, strongly suggesting that similar mechanisms will be at play in the latter strain and thereby implying the conditional use of futile reactions in central metabolism as regulatory mechanisms.

For both the *mucA* Δ*algC* and *mucA* TT*algD* strains, the *rpiA* encoded ribose-5-phopshate isomerase A (PFLU5824) appears transcriptionally down-regulated compared to the wild type, with evidence scores ranking 4th and 10th in the two former strains, respectively. As suggested for the *mucA* strain, this could represent a reduced activity of the pentose phosphate pathway. For the wild-type and the Δ*algC* strains, the cycle (1)–(4) could re-oxidise surplus NADPH generated by the higher flux through the pentose phosphate pathway, effectively generating a cycle dissipating reductive potential. Phenotypically, this would then translate into lower biomass yield, observable as higher relative respiration for the wild-type and the Δ*algC* strains (see Table 2).

#### Wild type versus *mucA* TT*algD* in glycerol

When glycerol is used as a carbon source, the random sampling algorithm could not identify any significant transcriptionally regulated fluxes in the alginate non-producing *mucA* TT*algD* mutant relative to the wild type.

It is noteworthy that the reactions constituting the NADPH consuming cycle suggested above for two of the fructose-grown strains do not come out as significantly transcriptionally regulated in the wild type–*mucA* TT*algD* comparison on glycerol. The genes *aatB* and *katB* showed a down-regulation in the *mucA TTalgD* with respect to the wild type that is as strong during growth on glycerol as on fructose; however the mentioned down-regulation does not seem to have any impact in the metabolic fluxes when glycerol is the carbon source, as the mentioned cycle is only modeled as active when the cells grow on fructose. This is in agreement with observed differences in respiration; if ratios of respiration per dry weight is calculated (from the values in Table 2) for the wild type relative to the *mucA* TT*algD* mutant, this ratio is significantly higher when fructose is used as a carbon source (wild type = 7.5 mmolC/g-DW hr; mutant = 3.9 mmolC/g-DW hr; ratio = 1.92) than when glycerol is used (wild type = 6.4 mmolC/g-DW hr; mutant = 5.0 mmolC/g-DW hr; ratio = 1.28). Both in relative and absolute terms, the change in respiration as a function of the *mucA* inactivation is clearly more profound on fructose than on glycerol again supporting the hypothesis of an NADPH oxidizing cycle in operation when the wild type cells are growing on fructose.

#### Wild type versus *mucA* in glycerol

When the wild type and the *mucA* strains were grown with glycerol as the sole carbon source five proteins encoded by genes from the alginate biosynthetic operon are ranked 1st to 5th in the list of transcriptionally regulated enzymes, which is similar to the observations for this strain on fructose. Within the 6th to 10th ranking enzymes, two are directly involved in glycerol uptake and conversion (fructose bisphosphate aldolase Fba [PFLU5701] and the GlpF glycerol uptake facilitator protein [PFLU1143]), two contribute to energy generation by oxidative phosphorylation (the Ndh NADH dehydrogenase II [PFLU0783] and ATP synthase) and one (Ndk; nucleoside diphosphate kinase, PFLU5061) generates GTP from ATP for consumption in alginate biosynthesis. It thus seems that all the top ranking enzymes suggested to be transcriptionally regulated can be linked directly to the uptake and conversion of the carbon source into alginate when the organism is grown on glycerol. This is in contrast to the situation on fructose as described above, and it could indicate that although only a few biochemical steps are different between the pathways leading to alginate from fructose and glycerol, respectively, the organism seems to regulate the metabolism surrounding the alginate biosynthetic precursors and cofactors quite differently on the two carbon sources. This could be of relevance if metabolic engineering of the alginate biosynthesis were to be pursued.

## Conclusions

We have described the reconstruction and application of a *P. fluorescens* SBW25 genome-scale metabolic model. Genetic engineering was used to construct selected strains with alterations in both the anti-sigma factor *mucA* and alginate-specific genes, and the physiological response was characterized by chemostat cultivations and transcriptome analysis. Integration of the metabolic model with the experimental data allowed us to obtain new insight about the metabolism of this producer of the commercially interesting biopolymer alginate. Firstly, we could show that the yields of alginate are close to the theoretical stoichiometric optimum, demonstrating the very high efficiency of the alginate biosynthetic machinery in this organism. We could also, however, see that the biomass production of *P. fluorescens* SBW25 is markedly suboptimal in the wild-type strain, and by the use of random sampling analysis in the transcriptome data set we could give strong indications that this suboptimality on fructose is related to a cycle in the central metabolism, effectively wasting cellular energy in the form of NADPH. This cycle does not seem to be active when the organism is grown on glycerol. The down-regulation of the suspected NADPH oxidizing cycle was observed in the *mucA* strains, adding to the putative regulon of this global regulator.

The model described in this work, validated by comprehensive continuous-culture experimental data and integrated with transcriptome analyses, nicely complements the existing *P. aeruginosa* and *P. putida* models. Alginate is a significant virulence factor in *P. aeruginosa* infections, and the emphasis on alginate biosynthesis in the (non-pathogenic) *P. fluorescens* system described here could support the clinical research on this important pathogen. Also, alginate is a valuable industrial product, and the optimization of alginate production in bacterial systems is of great commercial interest. As compared to the other major alginate-producing bacterial genus, *Azotobacter*, the relative simplicity that applies to both engineering and cultivating *P. fluorescens* – taken together with its highly efficient alginate biosynthesis as described here – should provide an argument for its applicability in an industrial context. The heterologous expression of *Azotobacter* alginate epimerases could then be used to produce high yields of alginates with tailored monomer sequences.

## Methods

### Strain construction

The plasmids and strains used in this study are described in Additional file 1. Plasmid isolations, enzymatic manipulations of DNA, agarose gel electrophoresis and other routine DNA manipulations were performed according to the methods of Sambrook and Russell [63]. The QIAquick Gel Extraction Kit and QIAquick PCR purification kit (Qiagen, Germany) were used for DNA-purifications from agarose gels and enzymatic reactions, respectively. PCR for cloning and allele identification was performed using the Expand High Fidelity PCR system (Roche Diagnostics, Switzerland). DNA was sequenced using the Big-Dye Terminator v1.1 Cycle kit (Applied Biosystems, CA). Transformations of *E. coli* were performed using the rubidium-chloride method (available at http://www.neb.com). Matings and selection of double recombinants were performed as described earlier [22,64].

### Cultivation and sampling

Triplicate chemostat experiments were performed in 3 liter fermenters (Applikon, Netherlands) with a 0.75 liter working volume and a feeding rate of 30 g/hr, corresponding to a dilution rate, D = 0.04. Inoculation of the chemostats was 3% from an overnight culture grown in LB medium (all growth media are described in Additional file 4), and the initial (batch phase) medium in the fermenter was Def4m. Temperature was controlled at 25°C, and pH was kept at 6.8 by addition of NaOH. Aeration was 0.5 liters per liter culture volume, and dissolved oxygen was controlled at 20% by adjusting stirrer speed. Feeding was started after 24 hrs, and was controlled on a weight basis. The chemostat feeding growth medium, Def4, was fully defined with either fructose or glycerol (both 40 g/l) as the carbon source. Exhaust CO_2_ was measured continuously by online mass spectrometry. Sampling was performed at steady state for offline analysis of remaining carbon source, alginate and transcriptome. Detailed sampling protocols can be found in Additional file 4. During the cultivations biomass was measured as OD_660_ and this was converted to biomass concentration in gDW/L using a conversion of 0.36 g/L OD unit.

### Carbon source, alginate and transcriptome analysis

Remaining carbon source in the chemostat growth medium was analyzed by HPLC on a 300x7.8 mm Aminex HPX-87H ion exchange column (BioRad, PA) at 45°C with 0.6 ml/min of 5 mM H_2_SO_4_ as the mobile phase. Alginate in the medium was quantified as described previously [11]. Since the alginate quantification assay operates on deacetylated alginate, acetylation had to be determined separately. This was done with the same anion exchange HPLC method that was used for quantification of residual carbon source. The acidic buffer used is sufficient to deacetylate alginate, so acetate can be measured directly.

Transcriptome analysis was done on a custom 385K 60-mer expression microarray (Roche NimbleGen Inc., WI) covering the *P. fluorescens* SBW25 genome. RNA was isolated with the RNEasy Midi Kit (Qiagen), and when necessary was up-concentrated with Microcon YM 30 spin columns (Millipore, MA). cDNA was produced from isolated RNA according to Roche NimbleGen Inc. instructions, from which hybridization and array scanning was purchased as a service. Analysis of the microarray data was done with GeneSpring GX software, version 11 (Agilent, CA).

### Metabolic network reconstruction and analysis

Reconstruction of the draft metabolic network in *P. fluorescens* SBW25 was based on the recently published annotated genome of the organism [21], and was done in a semi-automated fashion by using the Pathway Tools software suite [39], including the HoleFiller algorithm for closing gaps in the metabolic network. During the first phase of the reconstruction, the software automatically connects the gene products and reactions that can be unambiguously established from the genome annotation and sequence when compared to the existing inventory of genome/pathway databases. For enzyme–reactions associations that have a higher degree of uncertainty, the user is presented with the reactions that are hypothesized to be present in the network and potential enzymes (or, more accurately, their encoding genes) that could catalyse the reaction in question. The accurate assignment is then made based on manual curation. After this curation process is completed, an algorithm is called that identifies 'holes' in the metabolic network. A metabolic hole is a single biochemical reaction that is absent from an otherwise complete pathway, in such a way that filling of the hole yields a functional pathway that can carry a metabolic flux. If candidate gene products for filling the metabolic holes can be identified by the software, these are presented to the user for manual curation. This hole-filling is an iterative process, since introduction of new reactions into the network might justify the introduction of yet new pathways in the model, depending on the manually set cut-off for presence of a given pathway in the model. In the case of the draft model of *P. fluorescens* SBW25, the manual curation constituted verification of 90 enzyme–reaction connections and assignment of 43 protein complexes. 92 'holes' in the metabolic network were filled by manual curation, distributed as 74, 16 and 2 in the first, second and third iterations of the algorithm, respectively. Manual curation of the draft model was done by literature studies and use of online databases. Literature references used in the manual curation of the draft model are listed in Additional file 5.

The draft model was translated into the RAVEN Toolbox format [Agren *et al*., submitted; for overview see [41]] by the use of custom Perl scripts. Concomitantly, a further manual pruning of the model was performed. This included the removal of non-metabolic reactions and reactions with generalized substrates, dereplication of reactions performed by isoenzymes and alternative formulations of reactions as found in Pathway Tools, assignment of reaction irreversibility where applicable and verification of cofactor specificities. Furthermore, sequence and network comparison with the *i*JN746 and *i*MO1056 models for *P. putida* KT2440 and *P. aeruginosa* PAO1, respectively, allowed for addition of 38 reactions. Also, the pathways in the *P. fluorescens* SBW25 model defining fatty acid biosynthesis, glycerophospholipid metabolism and peptidoglycan biosynthesis were incomplete in the Pathway Tools formulation, and was manually completed by comparison with the *i*JN746 model as highly homologous gene products could be found for all reactions.

The final model was designated *i*SB1139*.* The model is available for download in both Excel format and in SBML at http://www.sysbio.se/biomet. Flux balance analysis of the SBW25 model was performed with the linear programming solver Mosek (Mosek ApS, Denmark) for Matlab (The Mathworks, MA).

## Competing interests

The authors declare that they have no competing interests.

## Authors' contributions

SEFB participated in construction of the custom expression array, performed microarray data analysis, contributed to analysis of physiological parameters, constructed the genome-scale metabolic model and performed comparisons with other species, and drafted the manuscript. SB performed the random sampling flux analysis, the calculations about theoretical yields and wrote part of the manuscript. HS and ØJ had major roles in cultivations, while HE was responsible for strain constructions. PB was involved in experimental design. TEE was leading the cultivation activities, JN was leading the modelling parts and SV was project leader for the overall project. All authors were to varying degrees involved in the planning, design and evaluation of the project, and read and approved the final manuscript.

## Supplementary Material

Additional file 1 An overview of the strains and plasmids used in this study. Click here for file

Additional file 2 The complete microarray data, both in raw and quantile normalized formats. Click here for file

Additional file 3 **A spreadsheet comparison and alignment of the reactions present in the published *Pseudomonas *genome-scale models *i*MO1056, *i*JN746 and *i*SB1139.** Click here for file

Additional file 4 Description of the growth media used for cultivations, and standard operating procedures used in sampling for transcriptome analysis, as well as analysis of alginate produced and remaining carbon source in the chemostat cultures. Click here for file

Additional file 5 The literature references used in the manual curation of the draft genome-scale metabolic model. Click here for file
