# Supplementary material for: Mapping global effects of the anti-sigma factor MucA in Pseudomonas fluorescens SBW25 through genome-scale metabolic modeling
Source: BMC Syst Biol. 2013 Mar 11;7:19. doi: 10.1186/1752-0509-7-19 (PMC3641028; doi:10.1186/1752-0509-7-19)
Supplement: Additional file 1 — An overview of the strains and plasmids used in this study. [file 1752-0509-7-19-S1.docx]

# Strain construction

The plasmids and strains used in this study are described in Table 1 below. Plasmid isolations, enzymatic manipulations of DNA, agarose gel electrophoresis and other routine DNA manipulations were performed according to the methods of Sambrook and Russell [[1](#_ENREF_1)]. The QIAquick Gel Extraction Kit and QIAquick PCR purification kit (Qiagen) were used for DNA-purifications from agarose gels and enzymatic reactions, respectively. PCR for cloning and allele identification was performed using the Expand High Fidelity PCR-system (Boehringer Mannheim). DNA was sequenced using the Big-Dye Terminator v1.1 Cycle kit (Applied Biosystems). Transformations of *E. coli* were performed using the rubidium-chloride method (available at [www.neb.com](http://micro.nwfsc.noaa.gov/protocols/rbcl.html)). Matings and selection of double recombinants were performed as described earlier [[2](#_ENREF_2), [3](#_ENREF_3)].

Table 1. Strains and plasmids in this study.

| **Strains** | **Description** | **Ref** |
| --- | --- | --- |
| *E. coli* S17.1 | RP4 2-Tc::Mu-Km::*Tn7* *pro res mod^+^* | [[4](#_ENREF_4)] |
| *P. fluorescens* SBW25 | Wild type | [[5](#_ENREF_5)] |
| *P. fluorescens* SBW25*mucA* | Derivative of wild type strain using pAT71 to introduce a stop codon in *mucA.* | This work |
| *P. fluorescens* SBW25 Δ*algC mucA* | Derivative of  *P. fluorescens* SBW25*mucA* where pKB22 were used to delete parts of *algC.* | This work |
| *P. fluorescens* SBW25Δ*algC* | Derivative of  *P. fluorescens* SBW25 Δ*algC mucA* using pAT70 to repair *mucA.* | This work |
| *P. fluorescens* SBW25*mucA* AlgD^-^ | Derivative of  *P. fluorescens* SBW25*mucA* using pMBN15 to insert a transcription terminator between P_algD_ and *algD.* | This work |
| **Plasmids** |  |  |
| pHE179 | ColEI cloning vector. Tc^r^, Ap^r^ | [[6](#_ENREF_6)] |
| pMG48 | RK2-based suicide vector encoding β-galactosidase; Tc^r^  Ap^r^ | [[3](#_ENREF_3)] |
| pAT70 | pMG48 based vector used to repair *mucA*. Tc^r^  Ap^r^ | [[6](#_ENREF_6)] |
| pAT71 | pMG48 based vector used to introduce a stop codon in *mucA*. Tc^r^  Ap^r^ | [[6](#_ENREF_6)] |
| pKB22 | pMG48 based vector used to delete *algC* | [[2](#_ENREF_2)] |
| pHE139 | RK2 derivative encoding P*_algD_* from *P. fluorescens* | [[6](#_ENREF_6)] |
| pHE142 | RK2 derivative encoding *algD* from *P. fluorescens* | [[6](#_ENREF_6)] |
| pMBN14 | Derivative of pHE179 where a 0.8 kb DNA fragment containing the *algD*-promoter, and a 1.6 kb DNA-fragment encoding *rrnB* and *algD* were inserted. | This work |
| pMBN15 | Derivative of pMG48 in which a 2.4 kb DNA fragment from pMBN14 containing *P_algD_-rrnB-algD* was inserted. Used to create a strain with no promoter before *algD*. | This work |

# Acknowledgments:

Heidi Myrset participated in making the plasmids and strains used in this study.

# References

1. Sambrook J, Russell D: **Molecular Cloning: A Laboratory Manual (Third Edition)**. New York: Cold Spring Harbor Laboratory Press; 2001.

2. Bakkevig K, Sletta H, Gimmestad M, Aune R, Ertesvåg H, Degnes K, Christensen BE, Ellingsen TE, Valla S: **Role of the *Pseudomonas fluorescens* alginate lyase (AlgL) in clearing the periplasm of alginates not exported to the extracellular environment**. *J Bacteriol* 2005, **187**(24):8375-8384.

3. Gimmestad M, Sletta H, Ertesvåg H, Bakkevig K, Jain S, Suh S-j, Skjåk-Bræk G, Ellingsen TE, Ohman DE, Valla S: **The *Pseudomonas fluorescens* AlgG protein, but not its mannuronan C5-epimerase activity, is needed for alginate polymer formation**. *J Bacteriol* 2003, **185**(12):3515-3523.

4. Simon R, Priefer U, Pühler A: **A broad host range mobilization system for in vivo engineering: Transposon mutagenesis in Gram negative bacteria**. *Biotechnology (N Y)* 1983, **1**:784-791.

5. Silby MW, Cerdeno-Tarraga AM, Vernikos GS, Giddens SR, Jackson RW, Preston GM, Zhang XX, Moon CD, Gehrig SM, Godfrey SA *et al*: **Genomic and genetic analyses of diversity and plant interactions of *Pseudomonas fluorescens***. *Genome Biol* 2009, **10**(5):R51.

6. Ertesvåg H, Degnes KF, Jørgensen H, Homberset H, Bakke I, Bakkevig K, Tøndervik A, Steigedal M, Ellingsen TE, Valla S *et al*: **Use of an inducible promoter and promoter mutant derivatives to analyze factors important for designing an alginate-overproducing strain of *Pseudomonas fluorescens***. *Manuscript in preparation*.
