# Supplementary material for: Mapping global effects of the anti-sigma factor MucA in Pseudomonas fluorescens SBW25 through genome-scale metabolic modeling
Source: BMC Syst Biol. 2013 Mar 11;7:19. doi: 10.1186/1752-0509-7-19 (PMC3641028; doi:10.1186/1752-0509-7-19)
Supplement: Additional file 4 — Description of the growth media used for cultivations, and standard operating procedures used in sampling for transcriptome analysis, as well as analysis of alginate produced and remaining carbon source in the chemostat cultures. [file 1752-0509-7-19-S4.docx]

# Supplementary File 1

### Inoculation growth medium LB

| Component | Concentration [g/l] |
| --- | --- |
| Tryptone | 10 |
| Yeast Extract | 5 |
| NaCl | 5 |

### Chemostat batch phase growth medium ‘Def4m’

| Component | Concentration [g/l] |
| --- | --- |
| Yeast extract | 0.500 |
| KH_2_PO_4_ | 0.648 |
| (NH_4_)_2_HPO_4_ | 2.750 |
| Citric acid*H_2_O | 0.900 |
| Fe(II) citrate hydrate | 0.0204 |
| H_3_BO_3_ | 0.00105 |
| MnCl_2_*4H_2_O | 0.005 |
| EDTA*2H_2_O | 0.0042 |
| CuCl*2H_2_0 | 0.0000525 |
| Na_2_Mo_4_O_4_*2H_2_O | 0.000875 |
| CoCl_2_*6H_2_O | 0.000875 |
| Zn(CH_3_COO)_2_*2H_2_O | 0.0026 |
| NaCl | 2.000 |
| MgSO_4_ | 0.616 |
| Clerol (antifoam) | 0.400 |
| Carbon source (fructose OR glycerol) | 40.0 |
| Water | (tap water) |

MgSO4, clerol and carbon source solutions were autoclaved separately and added directly to the fermenter.

### Chemostat fully defined growth medium ‘Def4’

| Component | Concentration [g/l] |
| --- | --- |
| KH_2_PO_4_ | 0.648 |
| (NH_4_)_2_HPO_4_ | 2.750 |
| Citric acid*H_2_O | 0.900 |
| Fe(II) citrate hydrate | 0.0204 |
| H_3_BO_3_ | 0.00105 |
| MnCl_2_*4H_2_O | 0.005 |
| EDTA*2H_2_O | 0.0042 |
| CuCl*2H_2_0 | 0.0000525 |
| Na_2_Mo_4_O_4_*2H_2_O | 0.000875 |
| CoCl_2_*6H_2_O | 0.000875 |
| Zn(CH_3_COO)_2_*2H_2_O | 0.0026 |
| NaCl | 2.000 |
| MgSO_4_ | 0.616 |
| Clerol (antifoam) | 0.400 |
| Carbon source (fructose OR glycerol) | 40.0 |
| Water | (tap water) |

MgSO_4_, clerol and carbon source solutions were autoclaved separately and added directly to the fermenter.

### Sampling for analysis of remaining carbon source

- Add 0.4 ml 0.6M perchloric acid to 1.5 ml Eppendorf tube
- Sample 0.6 ml culture to the 1.5 ml tube and mix with pipette
- Freeze at -20°C until HPLC analysis
- Centrifuge at 14 000 rpm for 10 mins
- Filter supernatant through 0.22 µm syringe filter

### Sampling for alginate analysis

- Sample 1.8ml culture to 2ml tube
- Centrifuge at 14 000 rpm for 15 mins
- Transfer 1000 µl supernatant to a new 1.5ml tube.
- Add 33µl 3M NaOH
- Freeze at -20°C until analysis

### Sampling for transcriptome analysis

- Add 4 x 40ml ice-cold 0.9% NaCl to 4 x sterile 50ml tubes on ice.
- Add 4 x 2ml culture to the 4 x 50ml tubes above. Invert tubes to mix.
- Centrifuge at 6000 G for 6 mins, 5°C
- Remove supernatant (remove last drops with Q-tips)
- Resuspend pellets in 4 x 2ml ice-cold 0.9% NaCl
- Add 4 x 2ml RNA Protect Bacteria Reagent (Qiagen, Germany)
- Combine two and two tubes with culture + RNA Protect into one 50 ml tube (2 x 4 ml).
- Incubate statically 5 min at room temperature
- Centrifuge at 3200 G for 10 min, 20°C
- Remove supernatant (remove last drops with Q-tips)
- Freeze pellet at -80°C
