# Supplementary material for: Mapping global effects of the anti-sigma factor MucA in Pseudomonas fluorescens SBW25 through genome-scale metabolic modeling
Source: BMC Syst Biol. 2013 Mar 11;7:19. doi: 10.1186/1752-0509-7-19 (PMC3641028; doi:10.1186/1752-0509-7-19)
Supplement: Additional file 5 — The literature references used in the manual curation of the draft genome-scale metabolic model. [file 1752-0509-7-19-S5.docx]

Contents

[Probable enzymes assignment 8](#_Toc303687057)

[PFLU0987 – AlgK 8](#_Toc303687058)

[PFLU0984 – AlgX 8](#_Toc303687059)

[PFLU0981 – AlgJ 8](#_Toc303687060)

[PFLU0980 – AlgF 8](#_Toc303687061)

[PFLU0989 – Alg8 8](#_Toc303687062)

[PFLU0988 – Alg44 8](#_Toc303687063)

[PFLU5706 – Epd (/GapB) 8](#_Toc303687064)

[PFLU4836 – Eda 8](#_Toc303687065)

[PFLU5919 – FolX 8](#_Toc303687066)

[PFLU0982 – AlgI 9](#_Toc303687067)

[PFLU5932 9](#_Toc303687068)

[PFLU4482 – CobC 9](#_Toc303687069)

[PFLU3931 – FolD 9](#_Toc303687070)

[PFLU0482 – HldE / RfaE 9](#_Toc303687071)

[PFLU5820 – NudH / YgdP 9](#_Toc303687072)

[PFLU1434 – PhaG 9](#_Toc303687073)

[PFLU4394 10](#_Toc303687074)

[PFLU5137/5138/5139/5140 10](#_Toc303687075)

[PFLU3271 – HpcC / HpaE 10](#_Toc303687076)

[PFLU3275 – HpcG / HpaH 11](#_Toc303687077)

[PFLU3276 – HpcH / HpaI 11](#_Toc303687078)

[PFLU3579 – NspC 11](#_Toc303687079)

[PFLU1865 – FadE 11](#_Toc303687080)

[PFLU0860 – GatB 12](#_Toc303687081)

[PFLU0618 – AccB 12](#_Toc303687082)

[PFLU4560 – CcoQ 12](#_Toc303687083)

[PFLU5759 – PyrC (PyrB component) 12](#_Toc303687084)

[PFLU3269 – HpcE / HpaG 13](#_Toc303687085)

[PFLU0331 – HisF 13](#_Toc303687086)

[PFLU3966 – BkdB 13](#_Toc303687087)

[PFLU0463, 0464, 0465 – WaaC, G, P (alternative names RfaC, G, P) 13](#_Toc303687088)

[PFLU3940 – AmaB 13](#_Toc303687089)

[PFLU3823, 3830 – NuoG, N and 0783 – ndh 14](#_Toc303687090)

[PFLU4182 – MetZ 14](#_Toc303687091)

[PFLU4533 – PpiA 14](#_Toc303687092)

[PFLU5017 – PurT 14](#_Toc303687093)

[PFLU5743, 42, 41, 40, 39, 38, 37, 36, 35 – MdcA, B, C, D, E, G, H, MadL, MadM (/McdL, McdM?) 14](#_Toc303687094)

[PFLU0031 15](#_Toc303687095)

[PFLU2150 – PbhA 15](#_Toc303687096)

[PFLU5940 – CyaA 15](#_Toc303687097)

[PFLU4482, 4484, 4487, 4488, 3211, 2666, 0604, 0607, 2670, 2669, – Cobalamin biosynthesis 15](#_Toc303687098)

[PFLU1647 17](#_Toc303687099)

[PFLU0346 18](#_Toc303687100)

[PFLU3944 18](#_Toc303687101)

[PFLU2304 – Gcd 18](#_Toc303687102)

[PFLU2323 18](#_Toc303687103)

[PFLU3208 18](#_Toc303687104)

[PFLU2328 – FolD 19](#_Toc303687105)

[PFLU4459, 4460 – PhhB, C 19](#_Toc303687106)

[PFLU2344 – RibBA 19](#_Toc303687107)

[PFLU0953 – LpxC 19](#_Toc303687108)

[PFLU1280 - LpxD 19](#_Toc303687109)

[PFLU0389 – UbiE 19](#_Toc303687110)

[PFLU5773 – ThiG 20](#_Toc303687111)

[PFLU0492 – ThiC 20](#_Toc303687112)

[PFLU1816, 1817 – SdhC, D 20](#_Toc303687113)

[PFLU4902 – NadA 20](#_Toc303687114)

[PFLU1063 – PdxJ 20](#_Toc303687115)

[PFLU0387 – UbiB 20](#_Toc303687116)

[PFLU5879 – UbiH 20](#_Toc303687117)

[PFLU6035 – UbiC 20](#_Toc303687118)

[PFLU0123 – TauD 21](#_Toc303687119)

[PFLU5400 – ThiE 21](#_Toc303687120)

[PFLU5774 – ThiS 21](#_Toc303687121)

[Rejected enzyme assignment 22](#_Toc303687122)

[PFLU4110 22](#_Toc303687123)

[PFLU3268 22](#_Toc303687124)

[PFLU0323 – MutY 22](#_Toc303687125)

[PFLU6061 – AphA 22](#_Toc303687126)

[PFLU3199 22](#_Toc303687127)

[PFLU5597, 5598, 5599, 5601, 5602 – PqqF, A, B, D, E 23](#_Toc303687128)

[PFLU2642 – MltD 23](#_Toc303687129)

[PFLU0283 – NudE 23](#_Toc303687130)

[PFLU5408 – Lnt 23](#_Toc303687131)

[PFLU0382 – Dtd 23](#_Toc303687132)

[PFLU5578 – KsgA 23](#_Toc303687133)

[PFLU1326 – EstC 23](#_Toc303687134)

[PFLU3186 - Ggt 23](#_Toc303687135)

[PFLU5119 – BphO 24](#_Toc303687136)

[PFLU3802 – Aat 24](#_Toc303687137)

[PFLU3699 – WaaE 24](#_Toc303687138)

[PFLU5416, 5417 – LipA, LipB 24](#_Toc303687139)

[PFLU5776 – MtgA(?) 25](#_Toc303687140)

[PFLU5585 – Cca 25](#_Toc303687141)

[PFLU0798 – AmpD 25](#_Toc303687142)

[PFLU3172 – NemA 25](#_Toc303687143)

[PFLU1657, 1658 – WbjB, C 25](#_Toc303687144)

[PFLU0880 – PtsN 25](#_Toc303687145)

[PFLU0394, 0395, 0396 – PhaC, B, A (and PFLU0391 – PhaI) 26](#_Toc303687146)

[PFLU3670 – WcaF 26](#_Toc303687147)

[PFLU3211 26](#_Toc303687148)

[PFLU5612, 5613 – BioC, BioH 26](#_Toc303687149)

[PFLU3943 – GltB 26](#_Toc303687150)

[PFLU0366 – HutH 26](#_Toc303687151)

[PFLU2547 – PvdF 26](#_Toc303687152)

[PFLU1586, 0614 – DusA, B 27](#_Toc303687153)

[PFLU3486 – MiaE 27](#_Toc303687154)

[PFLU3364, 3365 27](#_Toc303687155)

[Assigning protein complexes 28](#_Toc303687156)

[Thiazole synthase 28](#_Toc303687157)

[CobN - cobalamin cobalt insertion complex 28](#_Toc303687158)

[CarAB – Carbamoyl phosphate synthase 28](#_Toc303687159)

[ACC; acetyl CoA carboxylase 28](#_Toc303687160)

[Aspartyl/glutamyl-tRNA(Asn/Gln) amidotransferase 28](#_Toc303687161)

[Aspartyl/glutamyl-tRNA(Asn/Gln) amidotransferase 29](#_Toc303687162)

[Succinyl-CoA synthetase 29](#_Toc303687163)

[Phenylalanyl-tRNA synthetase 29](#_Toc303687164)

[Glycyl-tRNA synthetase 29](#_Toc303687165)

[Ubiquinol–cytochrome C reductase 29](#_Toc303687166)

[Cytochrome O / ubiquinol oxidase 29](#_Toc303687167)

[Protochatechuate 3,4–dioxygenase 30](#_Toc303687168)

[Benzoate 1,2–dioxygenase 30](#_Toc303687169)

[Ribonucleoside-diphosphate reductase 30](#_Toc303687170)

[Tryptophan synthase 30](#_Toc303687171)

[Nitrile hydratase (cobalt-containing) 30](#_Toc303687172)

[Branched-chain keto acid dehydrogenase 30](#_Toc303687173)

[3–isoropylmalate dehydratase 31](#_Toc303687174)

[Succinate dehydrogenase 31](#_Toc303687175)

[Anthranilate synthase 31](#_Toc303687176)

[Glutamate synthase 31](#_Toc303687177)

[Sarcosine oxidase 31](#_Toc303687178)

[Phosphoribosylaminoimidazole carboxylase 31](#_Toc303687179)

[NAD(P) tranhydrogenase 32](#_Toc303687180)

[Alkyl hydroxyperoxide reductase 32](#_Toc303687181)

[NADH dehydrogenase 32](#_Toc303687182)

[ATP synthase (F_0_F_1_) 32](#_Toc303687183)

[Cytochrome C oxidase 32](#_Toc303687184)

[Urease complex 32](#_Toc303687185)

[Acetolactate synthase 33](#_Toc303687186)

[Arginine–N–succinyltransferase 33](#_Toc303687187)

[ATP phosphoribosyltransferase 33](#_Toc303687188)

[Imidazole glycerol phosphate synthase 33](#_Toc303687189)

[Succinyl-CoA:acetoacetate-CoA transferase 33](#_Toc303687190)

[Sulfate adenylyltransferase 33](#_Toc303687191)

[Isocitrate dehydrogenase 34](#_Toc303687192)

[Rejected protein complexes 35](#_Toc303687193)

[D-ala-D-ala ligase A and B 35](#_Toc303687194)

[Glutamine synthetase 35](#_Toc303687195)

[Alanyl-tRNA synthetase 35](#_Toc303687196)

[Aspartate–semialdehyde dehydrogenase – Asd 35](#_Toc303687197)

[Allophanate hydrolase 35](#_Toc303687198)

[Riboflavin synthase / lumazine synthase 35](#_Toc303687199)

[Hole-filling, iteration 1 37](#_Toc303687200)

[β-alanine degradation I 37](#_Toc303687201)

[2-nitropropane degradation 37](#_Toc303687202)

[4-aminobutyrate degradation I 37](#_Toc303687203)

[4-aminobutyrate degradation II 37](#_Toc303687204)

[4-hydroxyproline degradation 37](#_Toc303687205)

[Acetate utilization and formation 37](#_Toc303687206)

[Acetyl CoA fermentation to butyrate 37](#_Toc303687207)

[ADP-L-glycero-β-D-*manno*-heptose biosynthesis 38](#_Toc303687208)

[Allantoin degradation II 38](#_Toc303687209)

[Arginine degradation I (and VI) 38](#_Toc303687210)

[Biosynthesis of 2’-(5’’-triphosphoribosyl)-3’dephospho-CoA 38](#_Toc303687211)

[Branched-chain α-keto acid dehydrogenase complex 39](#_Toc303687212)

[Catechol degradation to β-ketoadipate 39](#_Toc303687213)

[Citrulline biosynthesis 39](#_Toc303687214)

[Cobalamin biosynthesis II (late cobalt incorporation) 39](#_Toc303687215)

[Coenzyme A biosynthesis 39](#_Toc303687216)

[D-galactarate degradation / D-glucarate degradation 39](#_Toc303687217)

[Ethylene glycol degradation 39](#_Toc303687218)

[Fatty acid elongation – unsaturated II 40](#_Toc303687219)

[Fatty acid β-oxidation I 40](#_Toc303687220)

[Flavin biosynthesis 40](#_Toc303687221)

[Formaldehyde oxidation II (glutathione-dependent) 40](#_Toc303687222)

[FormylTHF biosynthesis I 40](#_Toc303687223)

[Fructose degradation to pyruvate and lactate (anaerobic) 40](#_Toc303687224)

[GDP-D-rhamnose biosynthesis 41](#_Toc303687225)

[GDP-mannose metabolism 41](#_Toc303687226)

[Gluconeogenesis 41](#_Toc303687227)

[Glucose degradation (oxidative) 41](#_Toc303687228)

[Glutamate degradation IV 41](#_Toc303687229)

[Glycine betaine degradation 42](#_Toc303687230)

[Glycogen degradation 42](#_Toc303687231)

[Histidine biosynthesis I 42](#_Toc303687232)

[KDO transfer to lipid IV_A_ 42](#_Toc303687233)

[KDO_2_-lipid A biosynthesis I 42](#_Toc303687234)

[Ketogluconate metabolism 42](#_Toc303687235)

[Lysine biosynthesis I 43](#_Toc303687236)

[Methylcitrate cycle 43](#_Toc303687237)

[Octane oxidation 44](#_Toc303687238)

[Peptidoglycan biosynthesis I 44](#_Toc303687239)

[Peptidoglycan biosynthesis II 44](#_Toc303687240)

[ppGpp biosynthesis 44](#_Toc303687241)

[Proline degradation II 44](#_Toc303687242)

[Purine degradation 44](#_Toc303687243)

[Purine nucleotides *de novo* biosynthesis I 44](#_Toc303687244)

[Salvage pathways of adenine, hypoxanthine, and their nucleosides 45](#_Toc303687245)

[Sulfate reduction I (assimilatory) 45](#_Toc303687246)

[Thiamine biosynthesis 45](#_Toc303687247)

[Trehalose biosynthesis V 45](#_Toc303687248)

[Trehalose degradation I (low osmolarity) 45](#_Toc303687249)

[Tryptophan biosynthesis 46](#_Toc303687250)

[Ubiquinone biosynthesis I (aerobic) 46](#_Toc303687251)

[UDP-*N*-acetyl-D-glucosamine biosynthesis 46](#_Toc303687252)

[UDP-glucose conversion 46](#_Toc303687253)

[Valine degradation I 46](#_Toc303687254)

[Pathway HoleFiller – iteration 2 47](#_Toc303687255)

[4-hydroxymandelate degradation 47](#_Toc303687256)

[Acetyl-CoA fermentation to butyrate II 47](#_Toc303687257)

[Adenosylcobalamin biosynthesis II (late cobalt incorporation)*^(95,96)^* 47](#_Toc303687258)

[Allantion degradation II / III 47](#_Toc303687259)

[Fatty acid elongation – unsaturated 47](#_Toc303687260)

[Fatty acid β-oxidation I 47](#_Toc303687261)

[Folate polyglutamylation I 47](#_Toc303687262)

[Formate to nitrate electron transfer 48](#_Toc303687263)

[Histidine degradation I 48](#_Toc303687264)

[Siroheme biosynthesis 48](#_Toc303687265)

[Trehalose biosynthesis V 48](#_Toc303687266)

[Valine degradation I 48](#_Toc303687267)

[Pathway Holefiller – Iteration 3 48](#_Toc303687268)

[Tetrahydrofolate biosynthesis II 48](#_Toc303687269)

[Uridine-5’-phosphate biosynthesis 48](#_Toc303687270)

# Probable enzymes assignment

These are gene products that were identified as putative enzymes by Pathway Tools, but that were not assigned a reaction by the automated reconstruction. Results and considerations of the manual curation are listed below.

### PFLU0987 – AlgK

Part of alginate transport/polymerization complex.

### PFLU0984 – AlgX

Necessary for alginate biosynthesis – function not entirely clear, but probably regulatory through interaction with MucD (AlgY) [[1](#_ENREF_1)].

### PFLU0981 – AlgJ

Involved in acetylation (with AlgI and AlgF); contains a conserved active-site histidine [[2](#_ENREF_2), [3](#_ENREF_3)].

### PFLU0980 – AlgF

Involved in acetylation (with AlgJ and AlgI); localised in periplasma; exact function unknown [[2](#_ENREF_2), [3](#_ENREF_3)].

### PFLU0989 – Alg8

*P. aeruginosa*: Necessary (and bottleneck) in alginate production [[4](#_ENREF_4)] – overexpression increased alginate significantly. Exact function unclear; some homology with glycosyltransferases.

### PFLU0988 – Alg44

*P. aeruginosa*: Necessary in alginate production [[5](#_ENREF_5)]. Periplasmic; possibly regulator affected by c-di-GMP, as binding domain is found in Alg44 [[6](#_ENREF_6)].

### PFLU5706 – Epd (/GapB)

*E. coli*: Erythrose-6-P dehydrogenase, closely related to GapA glyceraldehyde-3-P dehydrogenase, but (largely) functionally different [[7](#_ENREF_7)].

Erythrose-6-P + NAD^+^ + H_2_O 🡪 4-P-erythronate + NADH + 2H^+^.

EC 1.2.1.72

### PFLU4836 – Eda

KDPG aldolase (ED pathway enzyme) – crystal structure available for *P. putida* [[8](#_ENREF_8)]; high similarity to *E. coli* structure. Function unambiguous.

EC 4.1.2.14

### PFLU5919 – FolX

*E. coli*: In annotation given as ”D-erythro-7,8-dihydroneopterin tri P epimerase”, but BLAST also gives match to ”dihydroneopterin aldolase”. These enzymes apparently are very similar. L-monapterin (the product) is suggested to be a cofactor in *Pseudomonas*’ hydroxylation of phenylalanine to tyrosine [[9](#_ENREF_9)] Epimerisation is between triphosphates of dihydroneopterin and -monapterin. If it’s and aldolase, the rxns. are the synthesis of 6-hydroxymethyl-7,8-dihydropterin and glycolaldehyde from either 7,8-dihydro-D-neopterin or 7,8-dihydro-L-monapterin [[10](#_ENREF_10)]. **In both enzymes, all activities are present to some degree.** *folX* deletion does not affect growth in *E. coli*.

Assigns 3 rxns. to this protein, creating 6-hydroxymethyl-dihydropterin, epimerisation between triphosphates and the epimerisation of the non-phosphate compound.

### PFLU0982 – AlgI

Required for acetylation of alginate[[3](#_ENREF_3)], (putative) membrane protein. AlgI is found also in other bacteria; it is suggested that it is involved in esterification of surface or extracellular polysaccharides[[2](#_ENREF_2)].

### PFLU5932

Suggested as (positive) alginate regulator in annotation, but alignment indicates function in heme synthesis (HemX). In *P. freudenreichii* (Gram-positive) it has been implied in transport of heme[[11](#_ENREF_11)].

EC 2.1.1.107.

### PFLU4482 – CobC

This is listed as an α-ribazole-P phosphatase, but alignment indicates that it may also be a P-glycerate/PP-glycerate mutase. Indeed, these two enzymes are closely related, <http://www.ebi.ac.uk/interpro/IEntry?ac=IPR001345>.

EC 3.1.3.73

### PFLU3931 – FolD

Extremely conserved protein across species; a bifunctional enzyme related to methylene-THF. Crystal structure solved for E. coli [[12](#_ENREF_12)].

EC = 3.5.4.9

### PFLU0482 – HldE / RfaE

Bifunctional enzyme (D-β-D-heptose-7-P kinase and D-β-D-heptose-1-P adenylyltransferase). Called HldE in *E. coli* (experimentally characterised)[[13](#_ENREF_13), [14](#_ENREF_14)], but for some reason called RfaE in *Pseudomonas* – functionality is the same. Heptose-less LPS mutants can mostly survive, *but* it seems that *P. aeruginosa* is very sensitive[[14](#_ENREF_14)]. Highly conserved.

EC = 2.7.7.- and 2.7.1.-

### PFLU5820 – NudH / YgdP

Nudix hydrolase (by alignment with *E. coli*), hydrolysing adenosine polyphosphates[[15](#_ENREF_15)]. Seems to be involved in infections – possibly by silencing ”alarmons” [[16](#_ENREF_16)]. The localisation of the *nudH/ygdP* gene upstream of *ptsP* is also similar to *E. coli*.

Quite strongly conserved ([BLAST](PFLU5820%20-%20nudH%20or%20ygdP.htm)).

Functionality related to 3.6.1.41, but NudH has preference for Ap5A, not Ap4A.

EC 3.6.1.- and 3.6.1.41.

### PFLU1434 – PhaG

(R)-3-hydroxydecanoyl-ACP:CoA transacylase.

Extremely close match to (experimental) enzyme from *P. putida* KT2440 ([BLAST](PFLU1434%20-%20phaG.htm)), and it’s also biochemically characterized[[17](#_ENREF_17), [18](#_ENREF_18)].

### PFLU4394

This protein has very high sequence similarity to both propionyl-CoA carboxylases and acetyl-CoA carboxylases, but it is not clear what exactly is the substrate. A recent publication describes that the homologous protein in *P. fluorescens* Pf-5 [[19](#_ENREF_19)] is actually a (catabolic) **geranyl**-CoA carboxylase, AtuC, ivolved in catabolism of acyclic terpenes, which abound in plants. Existence of the *atuABCDEFGH* gene cluster in *P. fluorescens* (but not in *P. putida*) was concurrent with ability to grow on acyclic terpenes (which *P. putida* couldn’t). Gene inactivation (in *P. aeruginosa*) confirmed the function of the *atu* genes – aa sequence identity with AtuC in *P. fluorescens* Pf-5 was 82%. This gene is annotated as *accD1* in Pf-5 (also PFL4196), which may be an error, as the name indicates an acetyl-CoA carboxylase. PFLU4394 is homologous to PFL4196 as found by tblastx (ACT).

The *P. aeruginosa* PAO1 protein is PA2888 /locus AAG06276 (<http://www.ncbi.nlm.nih.gov/entrez/query.fcgi?cmd=Retrieve&db=Protein&list_uids=9948979&dopt=GenPept>)

Sequence identity of SWB25 to *P. aeruginosa* is 80% ([BLAST](PFLU4394%20-%20AtuC.htm)).

EC = 6.4.1.5

### PFLU5137/5138/5139/5140

There seems to be some confusion about the nomenclature here, but the gene is called *cyoB*, and is involved in ubiquinol oxidase / cytochrome oxidase complex. KEGG: <http://www.genome.ad.jp/dbget-bin/show_pathway?ko00190+ko:K02298>

From the review [[20](#_ENREF_20)] bacterial oxidases can use either cytochrome C or membrane-bound quinol as substrate; the former are named cytochrome C oxidases and the latter quinol oxidases *and they are not equivalent*, contrary to what the naming sometimes seem to suggest. Subunit I (CyoB) i apparently is unit most conserved between the cyt.C and quinol oxidases, whereas substrate specificity resides in subunit II (CyoA, PFLU5140).

In *E. coli* the *cyo* genes constitute the oxidase that is used under high oxygen conditions [[21](#_ENREF_21)] and are cloned [[22](#_ENREF_22)]. *In vitro* experiments demonstrate the oxidation of ubiquinol [[23](#_ENREF_23)] by purified terminal oxidase, and it is shown that cytochrome C is not a substrate [[24](#_ENREF_24)] in the characterisation of the enzyme (complex). The function in *Pseudomonas* seems to be based on sequence similarity rather than functional studies.

[BLAST](PFLU5140%20-%20cyoA.htm) for *cyoA*, and [BLAST](PFLU5139%20-%20cyoB.htm) for *cyoB* both show very high similarity to *E. coli*.

EC 1.9.3.1

### PFLU3271 – HpcC / HpaE

Difficult to get the original paper describing the enzyme in *P. putida* (**Alonso, J. M., and A. Garrido-Pertierra.** 1986. Carboxymethylhydroxymuconic semialdehyde dehydrogenase in the 4-hydroxyphenylacetate catabolic pathway of *Pseudomonas putida*. Biochem. Cell Biol. **64:**1288-1293.), but the enzyme is described in *E. coli* [[25](#_ENREF_25), [26](#_ENREF_26)] as part of the 4-HPA catabolic pathway. NB! Nomenclature of this enzyme seems to vary between HpcC and HpaE depending on which pathway is being studied.

Nevertheless, [BLAST alignment](PFLU3271%20-%20hpcC.htm) of PFLU3271 and HpcC, as described in the homoprotocatechuic acid pathway [[27](#_ENREF_27)] shows extremely high similarity (E-value 0.0), and the function of the enzyme is stated as unambiguous there.

NAD is the preferred oxidising cofactor, but the *E. coli* enzyme seems to be able to use NADP to some extent [[25](#_ENREF_25)].

EC = 1.2.1.60

### PFLU3275 – HpcG / HpaH

(Same nomenclature variation as described for PFLU3271)

[BLAST similarity](PFLU3275%20-%20hpcG.htm) very high to HpcG / HpaH 2-oxo-hepta-3-ene-1,7-dioic hydratase (OHED hydratase) from *E. coli [*[*26*](#_ENREF_26)*,* [*27*](#_ENREF_27)*]*. This enzyme adds water to a double bond without energetic cofactors (just Mg^2+^). The enzyme has been crystallised [[28](#_ENREF_28)].

Difficult to find EC number (only defined as 4.2.1.-), but by looking into PathwayTools, it is clear that the substrate here is called 2-hydroxyhepta-2,4-dienedioate, and the product 4-hydroxy-2-ketopimelate. The structures vary a bit from some papers, because of (spontaneous) keto-enol-iomerisation.

Note:

In PathwayTools, 2-oxo-hept-3-ene-1,7,-dioate, which is really the isomeric substrate of this rxn., is just left as a ”dead-end” in equilibrium with 2-hydroxyhepta-2,4-dienedioate, and the latter is used in the reactions. This is strictly speaking wrong, but should not affect the model as such.

### PFLU3276 – HpcH / HpaI

[BLAST similarity](PFLU3276%20-%20hpcH.htm) to *E. coli* and location on chromosome strongly suggest that this enzyme catalyses the last step in the hpc pathway, the aldol cleavage to form pyruvate and succinic semialdehyde [[26](#_ENREF_26)]. The function of the *E. coli* HpcH analog has been described [[29](#_ENREF_29), [30](#_ENREF_30)].

EC from PathwayTools: 4.1.2.-

### PFLU3579 – NspC

It has been shown that *nspC* is essential in *H. pylori* [[31](#_ENREF_31)] and the authors speculate that this may be connected to the lack of *speB, -C* and *–D* genes required for spermidine biosynthesis, and that NspC could fulfill that role. It is not clear whether *nspC* is essential in *P. fluorescens*.

Recently found to be a carboxynorspermidine carboxylase as alternative to SpeE spermidine synthase[[32](#_ENREF_32)].

### PFLU1865 – FadE

Extremely conserved within *Pseudomonas* – and also in relation to *e.g.* *E. coli* (E=0.0). This is an acyl-CoA dehydrogenase that catalyses the initial step in fatty acid degradation [[33](#_ENREF_33)].

Another acyl-CoA dehydrogenase is described in *P. putida* KT2440 that only takes short-chain substrates [[34](#_ENREF_34)] (PP2216), but this is not the same gene (PP1893) that aligns with PFLU1865 in [BLAST](PFLU1865%20-%20FadE.htm).

EC 1.3.99.3

### PFLU0860 – GatB

Identity unambiguous by [BLAST](PFLU0860%20-%20gatB.htm) with the functionally characterised homolog from *P. aeruginosa* PAO1 [[35](#_ENREF_35)].

Transamidates Asp-tRNA[Asn] to Asn-tRNA[Asn] by the help of Gln🡪Glu.

NB! The enzyme can also perform transamidation of (heterologous) Glu-tRNA[Gln] to Gln-tRNA[Gln], but there is no substrate for this rxn. in the host, so it is not necessary.

EC 6.3.5.6

### PFLU0618 – AccB

Identified unambiguously by [BLAST](PFLU0618%20-%20accB.htm) with the functionally characterised homolog from *P. aeruginosa* PAO1 (P37799) [[36](#_ENREF_36)], a biotin carboxyl carrier protein subunit of acetyl-CoA carboxylase.

### PFLU4560 – CcoQ

Cytochrome C oxidase (cbb3-type) subunit; a homolog with high [BLAST](PFLU4560.htm) similarity is functionally described in *P. stutzeri* [[37](#_ENREF_37)]. *ccoQ* is part of a cluster *ccoNOQP*; the CcoN, O and P subunits are functionally well understood [[37](#_ENREF_37)], but the CcoQ is more cryptic.

In *Rhodobacter sphaeroides*, CcoQ protects the CcoNOP complex [[38](#_ENREF_38)] under highly aerobic conditions, but was not essential for oxidase activity of the complex. Authors suggest that it could be an evolutionary remnant.

*P. aeruginosa* contains two cbb3-clusters (located together on the chromosome) [[39](#_ENREF_39)], which are selectively used under different oxygen tension. From Artemis, it seems like *P. fluorescens* SBW25 also contains two clusters, PFLU4553–4556 and 4558–4561.

EC 1.9.3.1 (subunit)

PFLU4554 = CcoO1

PFLU4555 = CcoQ1

PFLU4558 = CcoN2

PFLU4559 = CcoO2

NB! In *P. aeruginosa* a ccb3 cyt.ox. is found to be part of the AlgR regulon!

### PFLU5759 – PyrC (PyrB component)

A dihydroorotase (DHOase), but *inactive* by similarity with the homologous enzyme in *P. aeruginosa* PAO1[[40](#_ENREF_40)] (P0401, [BLAST](PFLU5759%20-%20pyrC.htm)). *P. aeruginosa* contains two active PyrC, and this inactive one.

It has been shown in *P. putida* [[41](#_ENREF_41)] that the inactive PyrC (denoted PyrC’) is required as a structural component for activity of PyrB (an aspartate transcarbamoylase, ATCase).

EC 2.1.3.2

### PFLU3269 – HpcE / HpaG

Annotated as "fumarylacetoacetate (FAA) hydrolase family protein", but FAA (EC 3.7.1.2) is only described in mammals (no prokaryote hits in PubMed either); this seems highly unlikely. However, decent [BLAST](PFLU3269%20-%20hpcE.htm) hits (E=4e-38) are found against experimentally verified *E. coli* HpcE / HpaG (nomenclature depends on which *E. coli* strain is studied) [[26](#_ENREF_26), [42](#_ENREF_42)]. This is a bifunctional enzyme in the degradation of HPC / 4-HPA, and in *P. fluorescens* SBW25, the PFLU3269 gene is located clustered with the other *hpc / hpa* genes.

EC 4.1.1.68 **and** 5.3.3.10 (bifunctional).

### PFLU0331 – HisF

Extremely conserved across species ([BLAST](PFLU0331%20-%20hisF.htm)), and localised together with *hisBHA*. HisBH makes a complex that catalyse the synthesis of D-erythro-imidazole-glycerol-phosphate in histidine synthesis by transmamidation from glutamine [[43](#_ENREF_43)]. This reaction has not been assigned a proper EC number (2.4.2.-) but exists in PathwayTools.

PFLU0328 is HisH.

### PFLU3966 – BkdB

Nice [BLAST](PFLU3966%20-%20BkdB.htm) match (P09062) against experimentally verified protein in *P. putida* [[44](#_ENREF_44)], the genes in *P. putida* were later cloned [[45](#_ENREF_45)].

This is the E2 component of the dehydrogenase complex, which has EC number 1.2.1.25.

### PFLU0463, 0464, 0465 – WaaC, G, P (alternative names RfaC, G, P)

Excellent [BLAST](PFLU0463%20-%20waaC.htm) homology with experimentally verified gene *waaC* in *P. aeruginosa* [[46](#_ENREF_46)] (acc.no. AAC45365), a heptosyltransferase involved in LPS core biosynthesis. There is also a patent (<http://www.google.com/patents?hl=en&lr=&vid=USPAT6444804&id=cR8LAAAAEBAJ&oi=fnd>) describing the *waaFCGP* cluster and a related GenBank acc.no. AAC33167 (no publication). There, it is described that WaaC (RfaC) adds the first heptose residue on KDO core, whereas WaaF (upstream *waaF* gene) adds the second.

WaaG in *E. coli* transfers a glucose moiety [[47](#_ENREF_47)], but the LPS structure is different in *P. aeruginosa* at this point – it contains a galactosamine residue instead [[46](#_ENREF_46)]

EC 2.4.1.58.

The same goes for the phosphorylating WaaP – the substrate is different from the *E. coli* enzyme.

EC 2.7.1.-

### PFLU3940 – AmaB

Seems like *P. fluorescens* SBW25 contains (at least) two *amaB* genes; PFLU3940 and 3987. Quite strongly [conserved](PFLU3940%20-%20amaB.htm) – assume annotation is correct here.

Both PFLU3940 and 3987 give very strong hits against the same *P. aeruginosa* protein (locus AAG03833), so they are essentially the same protein.

EC 3.5.1.87

### PFLU3823, 3830 – NuoG, N and 0783 – ndh

NADH dehydrogenase subunits; all other subunits already assigned – all genes are clustered. This system is very conserved and well characterised [[48](#_ENREF_48)]. Recently, the genes from *P. fluorescens* WCS365 (almost perfect BLAST match) have been characterised [[49](#_ENREF_49)]. They also found a second cluster *ndh* in *P. fluorescens* WCS365 for NADH dehydrogenase; the homolog in SBW25 is PFLU0783.

Note: Nuo enzymes can use both NADH and deamino-NADH as substrates.

EC 1.6.5.3 and the more general 1.6.99.5 both apply.

### PFLU4182 – MetZ

Virtually identical to MetZ from *P. putida* [[50](#_ENREF_50)]([BLAST](PFLU4182%20-%20metZ.htm), acc.no. AAK29460). It is desccribed that the substrate for this enzyme in *P. aeruginosa* is O-succinyl-homoserine, but even if the *P. putida* paper is somewhat unclear, it seems that the *P. putida* protein is more similar to the *P. syringae*, which uses O-acetyl-homoserine.

EC 2.5.1.49

### PFLU4533 – PpiA

Peptidyl-prolyl-cis-trans isomerase; strongly conserved. This enzyme (periplasmic) helps refold misfolded proteins by isomerising proline residues.

EC 5.2.1.8 – not connected to any pathways in PathwayTools.

### PFLU5017 – PurT

Strongly [conserved](PFLU5017%20-%20purT.htm) across species – phosphoribosylglycinamide formyltransferase 2. Characterised in *E. coli* [[51](#_ENREF_51)] (acc.no. 1EYZ_A). This enzyme differs from PurN in that it uses formate and not formyl tetrahydrofolate as formate donor.

### PFLU5743, 42, 41, 40, 39, 38, 37, 36, 35 – MdcA, B, C, D, E, G, H, MadL, MadM (/McdL, McdM)

Perfect hit (MdcA [BLAST](PFLU5743%20-%20mdcA.htm), acc. no. BAA36204; McdM [BLAST](PFLU5735%20-%20mdcM.htm), acc.no BAA36212) against experimentally verified malonate/malonyl-CoA decarboxylase from *P. putida* [[52](#_ENREF_52)].

MdcACDEH constitute the carboxylase subunits (EC 4.1.1.9).

MdcB is responsible for synthesis of the ACP prosthetic 2’-(5’’-phosphoribosyl)-3’-dephospho-CoA (EC 2.7.8.25).

McdG is responsible for attachment of the prosthetic group to the ACP to make the active enzyme.

Note: This prosthetic group is also used in citrate lyase! (EC 4.1.3.6)!

MdcLM are malonate transporters.

Malonate metabolism is reviewed in [[53](#_ENREF_53)].

### PFLU0031

[BLAST](PFLU0031.htm) suggest function as KPDG aldolase, but homology isn’t very high (falls off), and the gene is located among putative galactonate-converting enzymes; probably EC 4.1.2.21 (the KPDG aldolase working on the galactonate substrate, KPDGal, making G3P and pyruvate).

The pathway is active in *A. vinelandii* and several *Pseudomonas* [[54](#_ENREF_54)]. The upstream and downstream genes (PFLU0030/*dgoK* and PFLU0032/*dgoD*) are already assigned by PathwayTools.

EC 4.1.2.21

### PFLU2150 – PbhA

Very nice [BLAST](PFLU2150%20-%20phbA.htm) similarity to the *Azotobacter* FA8 /*vinelandii* (experimentally verified) enzyme [[55](#_ENREF_55), [56](#_ENREF_56)] (acc.no. CAC41637). The *phbBC* genes does not seem to be co-localised, but is has been described that *A. vinelandii* has several β-ketothiolase genes [[56](#_ENREF_56)].

EC 2.3.1.9

### PFLU5940 – CyaA

Adenylate cyclase; a large protein (800aa) with good [similarity](PFLU5940%20-%20CyaA.htm) to the *E. coli* gene [[57](#_ENREF_57)] (E=6e-117; acc.no. P00936). One other adenylate cyclase candidate in the SBW25 genome – an *exoY* homolog to *P. aeruginosa* (PFLU1622).

EC 4.6.1.1

### PFLU4482, 4484, 4487, 4488, 3211, 2666, 0604, 0607, 2670, 2669, – Cobalamin biosynthesis

Litterature: [[58-60](#_ENREF_58)] Nice, recent review with genome organisation from *P. fluorescens* in [[61](#_ENREF_61)]. Review of biosynthesis (from 2002) in [[62](#_ENREF_62)].

* = enzyme is already assigned by PathwayTools

[r] = reverse strand

The cobalamin biosynthesis genes seem to be organised in several clusters:

*[r] PFLU4491 🡪 assigned by PathwayTools as CobO (EC 2.5.1.17)

*[r] PFLU4490 🡪 assigned by PathwayTools as CobB (EC6.3.5.9)

[r] PFLU4489 🡪 ”putative oxidoreductase”; not assigned

[r] PFLU4488 🡪 CobD

[r] PFLU4487 🡪 ”putative cobalamin biosynthesis aminotransferase protein”

*[r] PFLU4486 🡪 assigned by PathwayTools; CobQ ”putative cobyric acid synthase protein” (EC 6.3.5.10)

[r] PFLU4484 🡪 putative cobinamide kinase / guanylyltransferase

*[r] PFLU4483 🡪 assigned by PathwayTools, CobT (EC2.4.2.21)

[r] PFLU4482 🡪 probably CobC; already flagged, see above

*[r] PFLU4481 🡪 assigned by PathwayTools, CobS (EC 2.7.8.26)

*[r] PFLU0602 🡪 CobK (EC 1.3.1.54)

*[r] PFLU0603 🡪 CobL (EC 2.1.1.132)

PFLU0604 🡪 probably CobG

*PFLU0605 🡪 CobH (EC 5.4.1.2)

*PFLU0606 🡪 CobI (2.1.1.130)

PFLU0607 🡪 probably CbiG

PFLU1078 🡪 probably CobW (”lonely” gene – no surronding *cob* genes)

PFLU1987 🡪 no gene name annotated (also ”lonely” *cob* gene)

*[r] PFLU2665 🡪 CobM (EC 2.1.1.133)

[r] PFLU2666 🡪 no gene name annotated

PFLU2669 🡪 CobW

PFLU2670 🡪 probably CobN (long protein, 1253aa)

[r] PFLU5331 🡪 no name annotated (possibly YjiA in *E. coli*); ”lonely” gene

[r] PFLU6083 🡪 no gene name annotated (possibly YeiR in *E. coli*)

[r] PFLU6085 🡪 no gene name annotated

Based on the analysis of genetic organisation in *P. fluorescens* [[61](#_ENREF_61)] quite a lot of the genes can be assigned, see below.

There seems to be some confusion in terms of nomenclature in the *P. fluorescens* SBW25 annotation, as the *Salmonella typhimurium* names are used for some – but not all – genes.

***PFLU4491:** CobO / BtuR; Cob(I)yrinic acid a,c-diamide 🡪 Ado-cobyrinic acid a,c-diamide; EC2.5.1.17

***PFLU4490:** CobB / CbiA; Hydrogenobyrinic acid 🡪 hydrogenobyrinic adis a,c-diamide; EC6.3.5.9

**PFLU4489:** BluB, proposed in Co reduction [[61](#_ENREF_61)] (probably wrong); the function was elucidated very recently [[63](#_ENREF_63), [64](#_ENREF_64)], and shown to be FMNH2 + O2 🡪 H2O + DMB + E4P, where DMB is 5,6-dimethylbenzimidazole. This rxn is not in BRENDA yet or in PathwayTools.

This enzyme is assigned to EC 1.16.8.1 but no evidence exists.

**PFLU4488:** CobD / CbiB; Ado-cobyrinic acid / -cobyrate + aminopropanol(-O-2-phosphate)🡪 Ado-cobinamide(-phosphate). Probably, the phosphorylated compounds are used here [[62](#_ENREF_62)].

EC6.3.1.10

***PFLU4486:** CobQ / CbiP; Ado-cobyrinic acid a,c-diamide 🡪 Ado-cobyrininc acid

EC 6.3.5.10

**PFLU4484:** CobP / CobU; Ado-cobinamide(-phosphate) 🡪 Ado-GDP-cobinamide

EC2.7.7.62

***PFLU4483:** CobU / CobT; DMB + NMN 🡪 α-ribazole-5P

EC 2.4.2.21

**PFLU4482:** ? / CobC; α-ribazole-5P 🡪 α-ribazole. The timing of the phosphatase activity is questioned [[62](#_ENREF_62)]; however, this should not complicate metabolic network.

EC 3.1.3.73

***PFLU4481:** CobV / CobS; Ado-GDP-cobinamide + α-ribazole 🡪 Ado-cobalamin

EC 2.7.8.26

***PFLU0602:** CobK; precorrin-6x 🡨 🡪 precorrin-6y

EC 1.3.1.54

***PFLU0603:** CobL; precorrin-6y 🡨 🡪 precorrin-8x

EC 2.1.1.132

**PFLU0604:** CobG; precorrin-3A + O2 🡪 precorrin-3B

EC 1.14.13.83

***PFLU0605:** CobH; precorrin-8x 🡪 Hydrogenobyrinic acid

EC 5.4.1.2

***PFLU0606:** CobI; precorrin-2 🡪 precorrin-3A

EC 2.1.1.130

**PFLU0607:** CbiG; contains large deletion 🡪 probably nonfunctional (only really needed in anaerobic B12 synthesis) [[61](#_ENREF_61)].

***PFLU2665:** CobM / CbiF; precorrin-3 🡪 precorrin-5

EC 2.1.1.133

**PFLU2670:** CobN; hydrogenobyrinic acid a,c-diamide + Co 🡪 Cob(II)yrinic acid a,c-diamide (complex with CobST / ChlDI = PFLU2671 and 2672).

EC 6.6.1.2

Also added PFLU2671 and 2672 to the same enzyme activity (cobalt chelatase subunit).

### PFLU1647

Annotated as a bifunctional protein, PFLU1647 is highly homologous ([BLAST](PFLU1647%20-%20tyrAc-aroF.htm), acc.no. AAD47362) to a ”supraoperon” in *P. stutzeri* and *P. aeruginosa*. The operon is functionally investigated in *P. stutzeri* [[65](#_ENREF_65)] and there is no doubt about gene functions, but in *P. stutzeri*, the two enzymatic functions (TyrAc and AroF) encoded in PFLU1647 are actually two different proteins, albeit separated only by 9 nt. The authors suggest that the two proteins are translationally coupled under at least some conditions.

Prephenate was shown to be a much better substrate than L-arogenate for the TyrAc activity [[66](#_ENREF_66)], but both activites should probably be assigned.

EC = 1.3.1.43 and 1.3.1.12

The AroF activity is a 5-enolpyruvylshikimate 3-P synthase.

EC = 2.5.1.19

### PFLU0346

**Periplasmic** chorismate mutase (AroQ) by similarity ([BLAST](PFLU0346%20-%20aroQ%20periplasmic.htm)) with the *P. aeruginosa* enzyme (acc.no. AAK73353). All active-site residues are conserved in *P. fluorescens* SBW25 [[67](#_ENREF_67)], even if overall similarity isn’t very good.

EC = 5.4.99.5

### PFLU3944

Strongly conserved protein (BLAST), with high similarity to dihydropyrimidine dehydrogenase from *Brevibacillus* (acc. no. AAO66291), where it is part of a *pydABC* gene cluster [[68](#_ENREF_68)]. PFLU3942 constitutes the PydB activity, so the identity of PFLU3944 to PydA is good. The PydA activity was never directly proven, but seems highly likely.

EC = 1.3.1.2

### PFLU2304 – Gcd

Membrane-bound, PQQ-dependent glucose dehydrogenase by [BLAST](PFLU2304%20-%20gcd.htm) similarity to *E. coli* [[69](#_ENREF_69)](acc.no. BAA02174).

EC = 1.1.5.2

### PFLU2323

Involved in glutamine biosynthesis; co-localised on the genome with *glxBCD* genes. Annotated as *glnT*, and [BLAST](PFLU2323%20-%20glnA.htm) (acc.no P31592) gives decent similarity to experimentally verified *Rhizobium* gene [[70](#_ENREF_70)]. And conserved residues in all species also seem to be conserved here [[70](#_ENREF_70)] by manual inspection.

EC = 6.3.1.2

### PFLU3208

General amidase; [BLAST](PFLU3208%20-%20amidase.htm) (acc.no P27765) gives excellent similarity to experimentally verified enzyme from *Pseudomonas chlororaphis* B23 [[71](#_ENREF_71)]. Gene organisation around is similar to *P. chl.*, indicating that PFLU3211 (CobW) is involved in production/nitrilase activity of PFLU3209/3210.

EC = 3.5.1.4

**PFLU3209** can be assigned by similarity as nitrile hydratase (subunit α; subunit β – PFLU3210 – is already assigned).

EC = 4.2.1.84

### PFLU2328 – FolD

Notoriously difficult to find experimental evidence for the [BLAST](PFLU2328%20-%20folD.htm) hits, even though the protein is highly conserved. Must resort to do [manual alignment](PFLU2328%20-%20folD%20Blast2P%20Ecoli.htm) against experimentally verified *E. coli* enzyme [[72](#_ENREF_72)], which gives 50% identity and E-value of 3e-63, *i.e.* good match. Also, all the highly conserved amino acids are conserved in the SBW25 protein.

EC = 1.5.1.5 and 3.5.4.9

### PFLU4459, 4460 – PhhB, C

PFLU4459

Nearly perfect similarity ([BLAST](PFLU4459%20-%20phhB.htm)) to *P. aeruginosa* protein PhhB (acc.no. P43335), experimentally verified [[73](#_ENREF_73)] as 4-α-carbinolamine dehydratase.

EC = 4.2.1.96

PFLU4460 – ”AspC”, reannotated as PhhC

By homology to gene organisation (*phhABC*) operon in *P. aeruginosa*, we can confidently say that this is wrongly annotated, even if the enzyme can also compensate for AspC activity at high expression levels [[74](#_ENREF_74)]. Substrates were found to be

L-aspartate (EC 2.6.1.1)

L-phenylalanine (EC 2.6.1.1 too)

L-tyrosine (preferred substrate) (EC 2.6.1.5)

### PFLU2344 – RibBA

Very conserved across species ([BLAST](PFLU2344%20-%20ribAB.htm)); best experiementally verified similarity is probably to *Photobacterium phosphoreum* (acc.no. BAC44851) [[75](#_ENREF_75)].

*P. fluorescens* SBW25 already contains the *rib* cluster (PFLU5470 and surrounding), so PFLU2344 may be inactive / regulated alternatively.

This rxns. doesn’t have EC number, but the enzyme is bifunctional:

The GTP cyclohydrolase II is EC 3.5.4.25

### PFLU0953 – LpxC

UDP-3-O-acyl-GlcNAc deacetylase, by [BLAST](PFLU0953%20-%20lpxC.htm) (acc.no. AAC44974) similarity to exp. verified *P. aeruginosa* enzyme [[76](#_ENREF_76)].

### PFLU1280 - LpxD

LpxD by both sequence similarity (original annotation) and co-localisation with *fabZ-lpxA-lpxB*.

Adds 3-OH myristoyl chain to lipidA (N-linked)

### PFLU0389 – UbiE

UbiE by sequence similarity in original annotation (69.5% id. to *E. coli*) [[77](#_ENREF_77)]. C-methylates demethyl-ubiquinone (EC = 2.1.1.64) and also demethyl-menaquinone (no EC number).

### PFLU5773 – ThiG

Good similarity with *E. coli* enzyme (original annotation), recently experimentally verified [[78](#_ENREF_78)].

Also: ThiI – PFLU0349 is assigned to same enzymatic activity.

### PFLU0492 – ThiC

Biosynthesis of thiamin precursor [[79](#_ENREF_79)] Hmp-PP.

### PFLU1816, 1817 – SdhC, D

By original annotoation, succinate dehydrogenase subunits. The *E. coli* analog (high similarity) uses ubiquinone as electron acceptor [[80](#_ENREF_80)] (SdhC is the ubiquinone-binding domain).

EC = 1.3.5.1

### PFLU4902 – NadA

Virtually perfect BLAST2P match to exp. verified enzyme in another *P. fluorescens* strain [[81](#_ENREF_81)].

Used for *de novo* NAD biosynthesis, but no EC number assigned.

Note: One *nadB* gene (PFLU1457) is annotated as pseudo, but is required for synthesis of quinolinate. There is another NadB (PFLU1465) that is complete.

Note 2: NadB is annotated as EC 1.4.3.16: O_2_ + H_2_O + L-aspartate 🡪 NH_3_ + H_2_O_2_ + oxaloacetate. The NH_3_ and oxaloacetate are products of spontaneous decomposition [[82](#_ENREF_82)] of iminoaspartate, which is really the product of NadB **and** the substrate for NadA (NadA-B is a complex).

### PFLU1063 – PdxJ

Very similar to *E. coli* enzyme (by original annotation), which has its function (in vitamin B6 synthesis) verified [[83](#_ENREF_83)].

EC = 2.6.99.2

### PFLU0387 – UbiB

Nice [similarity](PFLU0387%20-%20UbiB%20Blast2P_Ecoli.htm) with experimentally verified *E. coli* enzyme [[84](#_ENREF_84)]. Involved in coenzyme Q biosynthesis, but no EC number defined.

### PFLU5879 – UbiH

Good similarity with *E. coli* enzyme (original annotation). Function known [[84](#_ENREF_84)], but no EC number.

### PFLU6035 – UbiC

Identified by similarity (original annotation) and position relative to UbiA-encoding gene (operon) [[85](#_ENREF_85)].

### PFLU0123 – TauD

Original annotation shown high similarity to *E. coli* TauD, **even** if it is not localised in operon as in *E. coli*.

EC = 1.14.11.17

### PFLU5400 – ThiE

Decent similarity to *E. coli* (original annotation) ThiE, even though the *thiE* gene does not seem to be localised in operon.

EC = 2.5.1.3

### PFLU5774 – ThiS

OK similarity to *E. coli* enzyme (very short protein), and localised together with *thiG*.

Part of thiazole synthesis complex.

# Rejected enzyme assignment

This section contains proteinthat are considered putative metabolic enzymes by Pathway Tools, but that were rejected based on manual curation.

### PFLU4110

Annotated as *mhpA* by similarity with *E. coli*. MhpA is a 3-(3-hydroxyphenyl)propionate hydroxylase; however, PFLU4110 may more likely be a 3-(2-HP)P hydroxylase (”melilotate hydroxylase”) as is described from *Pseudomonas* [[86](#_ENREF_86)] earlier. The two enzymes take different substrates (and with narrow specificity), but the product, 2,3-dihydroxyphenyl-propionate, is the **same [**[**87**](#_ENREF_87)**]**. FAD is prosthetic group, whereas NADH is cofactor. It is the first step in the HPP catabolic pathway that eventually makes acetyl-CoA and feeds into TCA [[87](#_ENREF_87)].

If it is melilotate hydroxylase, the EC is 1.14.13.4 (rec. name ”melilotate 3-monooxygenase”).

### PFLU3268

Shows high similarity to regulatory protein HpaA involved in catabolism of 4-Hydroxyphenylacetate [[88](#_ENREF_88)], and more generally AraC-type **regulators** in aromatic compound degradation pathways [[89](#_ENREF_89)].

### PFLU0323 – MutY

[BLAST](PFLU0323%20-%20mutY.htm) shows it’s a highly conserved DNA repair enzyme. Most functional studies are done in *E. coli* [[90](#_ENREF_90), [91](#_ENREF_91)], but function has also been demonstrated in *P. aeruginosa* [[92](#_ENREF_92)]. The enzyme removes mismatched adenines.

The enzyme works on the same mismatch as MutM (EC 3.2.2.23), but on the opposite base [[92](#_ENREF_92)], and even though the function/identity is unambiguous, it does not seem to have been assigned an EC number yet.

Not a metabolic enzyme.

### PFLU6061 – AphA

Very likely an acetylpolyamine aminohydrolase by [BLAST similarity](PFLU6061%20-%20aphA.htm), but this is a very poorly described enzyme, only two occurences – the biological function is not clear [[93](#_ENREF_93), [94](#_ENREF_94)], but it may be involved in regulation and recycling of spermine/spermidine/putrescine [[94](#_ENREF_94)]. No EC number is assigned.

### PFLU3199

This is a somewhat particular enzyme; [BLAST](PFLU3199.htm) does not give analogs in other *Pseudomonas*. The sequence hits are against 3-oxoacyl-ACP synthases or beta-ketoacyl synthases, and the best hits are mostly against *Streptomyces, Bacillus* and *Synechoccus* – no matches in *E. coli*.

Probably involved in some PKS / FAS system, but not possible to determine exact reaction.

### PFLU5597, 5598, 5599, 5601, 5602 – PqqF, A, B, D, E

PqqC (PFLU5560) has been assigned by PathwayTools as a 'PQQ synthase' (EC 1.3.3.11), but is not connected to other pathways. The Pqq cluster makes pyrroloquinoline quinone presumably by fusion of conserved glutamate and tyrosine residues in PqqA [[95](#_ENREF_95)]. PpqF (the largest protein of the set) is quite conserved within *Pseudomonas* as determined by [BLAST](PFLU5597%20-%20ppqF.htm), but not entirely. Homology with other species falls off relatively quickly.

PQQ is a cofactor for (at least) bacterial dehydrogenases. The pathway isn’t elucidated further [[96](#_ENREF_96)], so it’s presently difficult to assign.

### PFLU2642 – MltD

Membrane-bound lytic murein transglycosylase D – exact funtion unknown, but involved in peptidoglycan degradation.

### PFLU0283 – NudE

Most likely ADP / nudix hydrolase, but exact substrate unknown. Homology not very high outside *Pseudomonas*.

### PFLU5408 – Lnt

Apolipoprotein N-acyltransferase [[97](#_ENREF_97)], quite conserved in [BLAST](PFLU5408%20-%20Lnt.htm). No entries for ”apolipoprotein” or smiliar activities in BRENDA.

### PFLU0382 – Dtd

D-tyrosyl-tRNA[Tyr] deacylase as suggested by [BLAST](PFLU0382%20-%20dtd.htm), but there is not much experimental evidence on this enzyme (mainly *E. coli*), so probably most of the hits are just assigned by seq. similarity. Could it be a more general aminoacyl deacylase?

### PFLU5578 – KsgA

An rRNA methyltransferase that is very conserved ([BLAST](PFLU5578%20-%20ksgA.htm)) throughout all organisms, the function is elucidated [[98](#_ENREF_98)] (as well as structure). Not essential, but knock-out increases doubling time and reduces virulence in *Yersinia*.

EC 2.1.1.48

Not a metabolic enzyme.

### PFLU1326 – EstC

Carboxylesterase, described from *P. fluorescens* [[99](#_ENREF_99)](almost perfect [BLAST](PFLU1326%20-%20estC.htm) hit). The original paper not available, but EC number is given in the abstract (EC 3.1.1.1, however, this is a generalized rxn.).

### PFLU3186 - Ggt

Gamma-glytamyltransferase/-transpeptidase; nice [BLAST](PFLU3186%20-%20ggt.htm) similarity (E=9e-110, locus AAA23869) to the experimentally analysed *E. coli* enzyme [[100](#_ENREF_100)]. This enzyme is unusual in that it contains both chains of a heterodimer, and thus is post-translationally processed.

EC 2.3.2.2

### PFLU5119 – BphO

Most likely a heme oxygenase; [BLAST](PFLU5119%20-%20bphO.htm) gives decent hits against other *Pseudomonas*, but the score falls off quickly. Best hit against experimentally verified protein is for *P. aeruginosa* PAO1 (locus PA4116, E=3e-17, 37% identities).

*P. aeruginosa* uses BphO to release iron from heme, and seems closely related to pathogenicity (also in *C. diphteriae*) where iron may be limited [[101](#_ENREF_101)]. Thus, this may be a protein that is necessary for *P. aeruginosa* but not the non-pathogenic *Pseudomonads*, which could explain the low homolgy score. Also, it could mean that the *P. fluorescens* BphO is non-functional. It is also mentioned that *bhpO* occurs in an operon with *bhpP* (downstream) in *P. aeruginosa*. The analog in *P. fluorescens* SBW25 is likely to be PFLU5120.

PathwayTools seems to have excluded BhpP from the possible list.

EC 1.14.99.3

### PFLU3802 – Aat

Leucyl/phenylalanyl-tRNA-protein transferase by [BLAST](PFLU3802-%20aat.htm) similarity with *E. coli* [[102](#_ENREF_102)]. Involved in degradation of proteins by transfer of an amino acid residue (Leu or Phe) to the N-terminus of the protein.

EC 2.3.2.6 is the rxn with leucyl-tRNA, but no EC number is found for the Phe rxn. However, 2.3.2.8 is an equivalent for Arg.

### PFLU3699 – WaaE

No [BLAST](PFLU3699.htm) hits against other *Pseudomonas* (!). Putative glycosyltransferase, but difficult to find experimental evidence. Annotators have assigned the name Waa, but this is most likely wrong. The *waaE* gene (a glycosyl transferase) is described in *Klebsiella pneumoniae [*[*103*](#_ENREF_103)*,* [*104*](#_ENREF_104)*]* but this is not the same protein. Also, there is some nomenclature confusion. PathwayTools suggest the rxn. described in *K. pneumoniae* (EC 2.4.-.-), but this is not sure enough.

### PFLU5416, 5417 – LipA, LipB

It seems that then name LipA is not unique – it is used both about lipoyl synthase, and (in *Pseudomonas*) about a lipase. Nice [BLAST](PFLU3416%20-%20lipA.htm) homology to the investigated *E. coli* enzyme [[105](#_ENREF_105)], which converts octanoyl-ACP to lipoyl-ACP by insertion of two sulfur atoms.

According to [[105](#_ENREF_105)], LipB (PFLU5417) catalyses the next step, which is transfer of the lipoyl group to at least three apo-enzymes, among others pyruvate dehydrogenase, to make the holo-enzyme. However, other publications show that the order of LipA and LipB action can be reversed, *i.e.* that octanoyl moiety is transferred to the apo-enzyme, and then converted to lipoyl on the enzyme [[106](#_ENREF_106), [107](#_ENREF_107)].

The EC number 2.8.1.8 covers LipA (and possibly LipB?) function, even though the rxn. seems quite ”lumped”.

EC 2.8.1.8 does not exist in PathwayTools, and neither does the csp. rxn.

EC 2.3.1.181 csp. to the transfer of octanoyl from ACP to a lysine residue, and is thus the LipB rxn. (at least with some of the substrate).

### PFLU5776 – MtgA(?)

Very difficult to find experimental evidence both in BLAST and PubMed, but quite conserved.

### PFLU5585 – Cca

This very conserved enzyme ([BLAST](PFLU5585%20-%20Cca.htm)) catalyse addition of the nucleotides cca to the 3’ end of tRNA [[108](#_ENREF_108), [109](#_ENREF_109)]. The identity is unambiguous (*E. coli* acc.no. P06961), and contains EC functions 2.7.7.21 and 2.7.7.25 (additional functions mentioned in annotation).

### PFLU0798 – AmpD

The *ampD* homolog from *P. aeruginosa* (acc.no. AAC98783, [BLAST](PFLU0798%20-%20ampD.htm)) has been characterised [[110](#_ENREF_110)]; function is unambiguous and related to β-lactam resistance. It is an N-acetylmuramoyl-L-alanine amidase involved in recycling of the cell membrane, and also functions as a negative regulator of the AmpC β-lactamase.

Note: It is shown in *A. vinelandii* that interruption of the *ampDE* operon increases *algD* transcription – and thus alginate synthesis [[111](#_ENREF_111)].

EC = 3.5.1.28

### PFLU3172 – NemA

Annotated as N-ethylmaleimide reductase. Very little is published on this enzyme, and N-ethylmaleimide seems to be used primarily as an inducer of cellular responses. The only direct publication [[112](#_ENREF_112)] is not easily available. Apparently, the enzyme catalyses reduction of a C=C double bond of five-membered ring compounds which have two conjugated carbonyl groups on both sides of the bond.

Not many hits against other *Pseudomonas* – this may be a non-ubiquitous enzyme. Similarity with morphinone reductase is also mentioned, and generally as an ”NADH:flavin oxidoreductase”.

### PFLU1657, 1658 – WbjB, C

Localised in cluster *wbjBCD*, where WbjD is already assigned by PathwayTools. The pathway (UDP-N-acetyl-L-fucosamine) has been elucidated in *P. aeruginosa* (locus AAD45266, acc.no. 147795) [[113](#_ENREF_113), [114](#_ENREF_114)]. ([BLAST](PFLU1658%20-%20wbjC.htm)). Pathway does not exist in the database collection.

### PFLU0880 – PtsN

Component of PTS system; highly homologous to the studied protein in *P. putida* [[115](#_ENREF_115)]([BLAST](PFLU0880%20-%20ptsN.htm), acc.no. 2007260A – note that the protein is called RpoN in the GenBank entry; this is wrong – the RpoN is a much larger protein, but the csp. gene is located just upstream). The authors do not describe any membrane-associated component, and thus suggest that the function is purely regulatory in terms of N-metabolism.

Upstream PFLU0878 is probably PtsO protein, which is a regulator.

### PFLU0394, 0395, 0396 – PhaC, B, A (and PFLU0391 – PhaI)

Polyhydroxyalkanoate-synthesis related (homology to *P. oleovorans* [[116](#_ENREF_116)]. Seems like these systems aren’t completely genetically defined.

### PFLU3670 – WcaF

Most likely involved in cholanic acid (CA) biosynthesis, both from annotation and the surrounding genes.

Reasonable [BLAST](PFLU3670%20-%20WcaF.htm) similarity to *E. coli* gene (acc.no. P0ACD2); the colanic acid genes have been elucidated to some extent [[117](#_ENREF_117)] and they are described in a recent review of EPS biosynth. and assembly in *E. coli* [[118](#_ENREF_118)]. However, the analogous genes in *P. fluorescens* are not organised in a completely similar manner, and some analogs are missing.

### PFLU3211

A CobW analog ([BLAST](PFLU3211%20-%20cobW.htm)).

Could be involved in nitrilase activity of PFLU3209+3210 [[71](#_ENREF_71)], but not entirely cleae.

### PFLU5612, 5613 – BioC, BioH

Biotin biosynthesis – the genes sit in the middel of *bioBFHCD* cluster. E-value ([BLAST](PFLU5612%20-%20bioC.htm)) outside of *Pseudomonas* falls off, but that is also due to short proteins. Reasonable similarity to *Serratia* experimentally verified proteins (acc.no. P36571, Q8GHL1).

Biotin biosynthesis in microbed is reviewed [[119](#_ENREF_119)], and BioC + BioH are required for the first step (synthesis of pimeloyl-CoA), but it is not clear what is the substrate for this rxn. Pimelic acid in *not* the substrate in Gram-negative bacteria, but it might be L-alanine and/or acetate [[120](#_ENREF_120), [121](#_ENREF_121)].

### PFLU3943 – GltB

Annotated as glutamate synthase subunit, but [BLAST](PFLU3943%20-%20gltB(possible).htm) doesn’t give clear hits against experimentally verified proteins from *Pseudomonas*. Also, SBW25 already contains this enzymatic activity (PFLU0414).

### PFLU0366 – HutH

Localised immediately upstream of (assigned) *hutH* gene (histidine-ammonia lyase) in *hut* gene cluster. The two proteins have reasonable [pairwise similarity](PFLU0366%20-%20hutH%20Blast2P%20-%200367.htm) (37% id., E=1e-77). General [BLAST](PFLU0366%20-%20hutH(possible).htm) gives that the protein is quite conserved across species, so it’s likely to have a function. Not clear if this is active.

The *hut* genes have been investigated [[122](#_ENREF_122)] in *P. fluorescens* SBW25, although that study doesn’t focus on the HutH proteins.

EC = 4.3.1.3

### PFLU2547 – PvdF

Very high similarity ([BLAST](PFLU2547%20-%20pvdF.htm)) to *P. aeruginosa* enzyme involved in pyoverdin (= fluorescein, the characteristic green siderophore) synthesis [[123](#_ENREF_123)].

Assumed rxn.: N5-hydroxyornithine 🡪 N5-formyl-N5-hydroxyornithine (formylation, but formyl donor not precisely known).

### PFLU1586, 0614 – DusA, B

Dihydrouridine synthases, modifies bases in the D-loops of tRNA [[124](#_ENREF_124)]. No EC number defined.

### PFLU3486 – MiaE

tRNA hydroxylase – good match (original annotation) against *S. typhimurium* verified enzyme [[125](#_ENREF_125)]. It was shown that mutants with inactive MiaE were unable to grow aerobically on the dicarboxylic acids of the TCA.

### PFLU3364, 3365

Either GlgAB or TreYZ – one of the genes is wrongly annotated anyway. Both sequences PFLU3364 and 3365 are very conserved ([BLAST](PFLU3364%20-%20GlgA.htm)) against GlgA and TreZ, respectively.

GlgA is assigned EC 2.4.1.21.

GlgB is putatively a branching enzyme, probably EC 2.4.1.18.

Not a lot is published on the trehalose synthesis, but apparently the glycogen and trehalose pathways are somewhat related [[126](#_ENREF_126)]. (The precursor is the same).

# Assigning protein complexes

The following protein complexes were suggested, but not assigned, by Pathway Tools, and were subsequently verified by manual curation. Where no external reference is mentioned, the E-value for sequence similarty within Pathway Tools as well as manual BLAST and analysis of gene organisation were considered sufficient for complex assignment.

### Thiazole synthase

PFLU0349(ThiI)+5773(ThiG)+5774(ThiS)

### CobN - cobalamin cobalt insertion complex

PFLU2670(CobN)+2671+2672

### CarAB – Carbamoyl phosphate synthase

PFLU5265(CarB)+5266(CarA)

### ACC; acetyl CoA carboxylase

Suggested complex:

PFLU0617 – AccC (immediately downstream of PFLU0618, AccB).

PFLU1286 – AccA

PFLU1997 – AccC

PFLU4187 – AccD

PFLU6071 – AccC

The acetyl CoA carboxylase complex [[127](#_ENREF_127)] consists of AccABCD, but it is not clear why there are more *accC* (biotin carboxylase) genes in *P. fluorescens* – this is not described in *E. coli*. Alignment of the three AccC proteins show that they are quite conserved.

PFLU4024-4025 also seems to be another AccCB pair, even if they are assigned different substrates.

AccB (PFLU0618) is the BCCP.

PFLU5543 (EC 6.3.4.15) is the protein-biotin ligase that catalyses this rxn.

Made complex PFLU0617+1286+4187

### Aspartyl/glutamyl-tRNA(Asn/Gln) amidotransferase

Suggested complex:

PFLU5856

PFLU3682

PFLU2057

Annotated as alkanesulfonate monooxygenase. PFLU3682 does not seem connected to anything and annotation seems ambiguous. 5856 and 2057 are both SsuD analogues and both localised together with SsuE analogues. PFLU5856 is located in an *ssuABCDE* locus / operon (with transporters) like the one described in *P. putida* [[128](#_ENREF_128)].

PFLU2057 is most likely MsuD (part of MsuCDE) rather than SsuD, as described in *P. aeruginosa* [[129](#_ENREF_129)], with some specificity for methanesulfonate.

Made complex PFLU2057+5856.

### Aspartyl/glutamyl-tRNA(Asn/Gln) amidotransferase

Suggested complex:

PFLU0861+0862 – glutamyl tRNA amidotransferase subunits. It seems extremely likely that PFLU0860+0861+0862 constitute a dual-specific amidotransferase for both glutamine and aspargine by similarity (via *P. putida*) with *Acidithiobacillus ferrooxidans [*[*130*](#_ENREF_130)*]*.

EC number 6.3.5.6 and 6.3.5.7.

Made complex PFLU0860+0861+0862

### Succinyl-CoA synthetase

Suggested complex:

PFLU1823+1824 – SucC+D – succinyl-CoA synthetase (Ssc).

The enzyme is well described, *e.g.* in *P. aeruginosa* [[131](#_ENREF_131)] – these are indeed subunits in a complex. Both Ssc and Ndk should probably be strongly upregulated in alginate synthesis stage.

### Phenylalanyl-tRNA synthetase

Suggested complex:

PFLU4143+4144 – phenylalanyl-tRNA synthetase α and β chains – PheTS

### Glycyl-tRNA synthetase

Suggested complex:

PFLU0010+0011 – glycyl-tRNA synthetase α and β chains – GlySQ

### Ubiquinol–cytochrome C reductase

Suggested complex:

PFLU0843+0841 – ubiquinol–cytochrome C reductase – PetCA

This is really a trimer complex PetABC [[132](#_ENREF_132)] (also called FbcFBC); PetB (PFLU0842) was included.

### Cytochrome O / ubiquinol oxidase

Suggested complex:

PFLU5137+5138+5140+5139+1901

PFLU1901 is CioB and PFLU1900 is CydA – these are both annotated as subunits of cytochrome D terminal oxidase. (CioB should most likely be CydB.)

PFLU5137–40 are subunits I–IV of CyoABCD; cytochrome O oxidase, *i.e.* different system.

In *E. coli* CyoABCD(E) and CydAB are two independent complexes performing the same rxn. under different oxygen tension. Assuming the same in *P. fluorescens*, PFLU1900 should be included in a complex.

Made complex PFLU5137–40

Assigned PFLU1900 to same rxn. as PFLU1901, and make this complex too; cytochrome D / ubiquinol oxidase

### Protochatechuate 3,4–dioxygenase

Suggested complex:

PFLU1366+1367 – PcaHG – protochatechuate 3,4–dioxygenase β and α chains

### Benzoate 1,2–dioxygenase

Suggested complex:

PFLU5194+5195 – BenAB – benzoate 1,2–dioxygenase α and β subunits.

This complex is described in (closely related) *Acinetobacter calcoaceticus* [[133](#_ENREF_133)], but also includes BenC / PFLU5196 (gene part of same operon) – was annotated with a very general electron transport rxn in PathwayTools.

Made complex PFLU5194+5195+5196

### Ribonucleoside-diphosphate reductase

Suggested complex:

PFLU2783+4726+4768 – ribonucleoside-PP reductase.

PFLU4726 and 4768 are most likely the α and β subunits that interact, as described in the heterotetrameric composition in *P. aeruginosa* [[134](#_ENREF_134)]. PFLU2783 is annotated as an alternative β subunit.

Made complex PFLU4726+4768

### Tryptophan synthase

Suggested complex:

PFLU0035+0036 – tryptophan synthase α + β chains – TrpAB

### Nitrile hydratase (cobalt-containing)

Suggested complex:

PFLU3209+3210 – nitrile hydratase α + β chains – Nhb

### Branched-chain keto acid dehydrogenase

Suggested complex:

PFLU3964+3965+3966 – BkdA1A2B – 2–oxoisovalerate dehydrogenase.

This complex actually also (physically) includes PFLU3967 (LpdV) [[45](#_ENREF_45)], but this protein is assigned another EC number.

### 3–isoropylmalate dehydratase

Suggested complex:

PFLU2049+2050+4195+4196 – 3–isoropylmalate dehydratase.

These are two sets of LeuCD complexes (also fits with genome localisation).

Made complexes PFLU2049+2050 **and** 4195+4196;

### Succinate dehydrogenase

Suggested complex:

PFLU1816+1817+1818+1819 – succinate dehydrogenase.

Since it is known that ubiquinone is electron acceptor, EC 1.3.5.1 should be used.

### Anthranilate synthase

Suggested complex:

PFLU1384+1385+5560+5561 – anthranilate synthase.

These are two isoenzyme complexes. Note that only PFLU5560+5561 sits together with other *trp* (anthranilate) genes.

Made complexes PFLU1384+1385 **and** 5560+5561

### Glutamate synthase

Suggested complex:

PFLU2326+2325+1107+0414+0415 – glutamate synthase.

The bacterial (NADP-dependent) enzyme consists of two subunits [[135](#_ENREF_135)]. Only PFLU1107 seems to be without its other subunit.

Made complexes PFLU2325+2326 **and** 0414+0415

### Sarcosine oxidase

Suggested complex:

PFLU5648–5651 + 2316–2319 – sarcosine oxidase Sox.

Two sets of α–δ subunits.

Made complexes 5648–5651 **and** 2316–2319

### Phosphoribosylaminoimidazole carboxylase

Suggested complex:

PFLU6054+6055 – phosphoribosylaminoimidazole carboxylase (PurKE).

ATPase and catalytic subunits – so this is a complex, although the catalytic subunit has some activity on its own.

### NAD(P) tranhydrogenase

Suggested complex:

PFLU0111+0113 – NAD(P) **transhydrogenase**, subunits β and α1 (PntBA).

Finds that PFLU0112 is also part of the 0111+0113 complex [[136](#_ENREF_136)].

### Alkyl hydroxyperoxide reductase

Suggested complex:

PFLU1152+2989 – alkyl hydroxyperoxide reductase (AhpCF).

Exists as complex in *E. coli*, but the partners are most likely PFLU2989 and 2990.

Made complex PFLU2989+2990

### NADH dehydrogenase

Suggested complex:

PFLU3820–3822 + 3825–3827 + 3829 – NADH dehydrogenase I.

Part of the *nuo* operon [[48](#_ENREF_48)].

### ATP synthase (F_0_F_1_)

Suggested complex:

PFLU6117–6125+4436 – ATP synthase.

The ATP synthase operon is strongly conserved in all bacteria [[137](#_ENREF_137)] with defined subunits; only the AtpI protein is not found in the complexes. However, experiments in *Bacillus* indicate a role in Mg2+ binding [[138](#_ENREF_138)].

Made complex without PFLU4436

### Cytochrome C oxidase

Suggested complex:

PFLU0058–0061 + 4553–4560 + 5345–5346 – cytochrome C oxidase.

PFLU0058–0061 (CtaCBGE) is clearly cytochrome C oxidase.

PFLU5345 and 5346 are annotated as CioB and CydA; this should be CioAB, homologous to a cyanide-insensitive terminal oxidase from *P. aeruginosa*.

PFLU4553–4556 and 4558–4561 are actually *two adjacent* CcoNOQP oxidase complexes, as found in *P. aeruginosa* [[39](#_ENREF_39)].

Made complexes PFLU0058­–0061 **and** PFLU5345+5346 **and** PFLU4553–4556 **and** PFLU4558–4561.

### Urease complex

Suggested complex:

PFLU0561–63 + 0578,0579,0582 – urease complex (UreABCEFG).

This complex is quite conserved [[139](#_ENREF_139)]; however, the genes in *P. fluorescens* SBW25 are not entirely co-localised.

Note: UreD (PFLU0583) should also be included in the suggested complex.

### Acetolactate synthase

Suggested complex:

PFLU1503+3087+5219+5220 – acetolactate synthase (IlvHI)

Based on similarity with *E. coli*, only PFLU5219+5220 in incorporated in the complex.

### Arginine–N–succinyltransferase

Suggested complex:

PFLU4755+4756 – arginine–N­–succinyltransferase. PFLU4756 is AruF, by similarity with *P. aeruginosa* [[140](#_ENREF_140)], whereas PFLU4755 is AruG. The Ast naming in the annotation is an *E. coli* remnant.

Note: The annotation of PFLU4758 as ArgD is somewhat misleading; for consistency it should be either AruC or AstC.

### ATP phosphoribosyltransferase

Suggested complex:

PFLU0524+0895 – ATP phosphoribosyltransferase (HisZG).

HisZ is a necessary subunit of HisZG, but has a regulatory role [[141](#_ENREF_141)]. It is quite conserved across species.

### Imidazole glycerol phosphate synthase

Suggested complex:

PFLU0328+0331 – imidazole glycerol phosphate synthase subunits (HisFH).

This is a complex, by similarity with other species.

### Succinyl-CoA:acetoacetate-CoA transferase

Suggested complex:
PFLU2151+2152 – succinyl-CoA:acetoacetate-CoA transferase (ScoAB).

Similarity of ScoA with *B. subtilis* enzyme with verified function [[142](#_ENREF_142)] is very high.

### Sulfate adenylyltransferase

Suggested complex:

PFLU4624+0760+0761 – sulfate adenylyltransferase.

PFLU0760 and 0761 are CysD and CysN; very high similarity with *E. coli* enzyme means complex can be inferred. PFLU4624 has low confidence level and is not considered here.

Made complex PFLU0760+0761

### Isocitrate dehydrogenase

Suggested complex:

PFLU3808+3809 – IcdA+Icd – isocitrate dehydrogenase.

There are some reports of bacteria with two ICD, among them *Acinetobacter* ADP 1 [[143](#_ENREF_143)], which is a close relative of *Pseudomonas*. An old paper describes another *Acinetobacter* where two ICDs have been functionally characterised [[144](#_ENREF_144)]. Functional characterisation has also been performed in *Mycobacterium tuberculosis* [[145](#_ENREF_145)], and possible roles in regulation of TCA vs. glyoxylate shunt are suggested.

# Rejected protein complexes

### D-ala-D-ala ligase A and B

Suggested complex:

PFLU0706+0949 – D-ala-D-ala ligase A and B. These are quite surely *not* a complex; two separate ligases are described in *E. coli* [[146](#_ENREF_146)], and in *S. typhimurium*, two alanine racemases are described, one constitutive and one inducible.

### Glutamine synthetase

Suggested complex:

PFLU2323 + 5849 + 5847 + 3065 + 2163 + 1514 + 0348 – gltuamine synthetase.

Some of these are annotated as GlnA, one is GlnT , and some are YcjK. As the GlnT seems to be involved in nitrogen sensing and assimilation, it is not unreasonable that the organism could have alternative synthetases. The YcjK protein is not described in PubMed or ISI, but Google Scholar gives hits that indicate role in the catabolism of polyamines [[147](#_ENREF_147)] like putrescine in *E. coli*. *S. coelicolor* has been shown to contain three additional *glnA*-type genes [[148](#_ENREF_148)] in addition to the two verified GSs, but the extra GSs can not complement an *E. coli* mutant, so they have other functions. This paper also states that GSs are homo-12-mers, *i.e.* not protein complexes anyway.

### Alanyl-tRNA synthetase

Suggested complex:

PFLU4627+4748 – alanyl-tRNA synthetase

Not a heteromeric aminoacyl-tRNA synthetase (as opposed to Phe and Gly); these enzymes reviewed in [[149](#_ENREF_149)].

### Aspartate–semialdehyde dehydrogenase – Asd

Suggested complex:

PFLU4191+4192 – aspartate–semialdehyde dehydrogenase – Asd

The *asd* gene is studied in *P. aeruginosa* [[150](#_ENREF_150)]; by similarity, PFLU4191 is probably not involved in the Asd phenotype, and not in a complex.

### Allophanate hydrolase

Suggested complex:

PFLU4022+4023 – allophanate hydrolase (subunits 1 and 2).

Allophanate hydrolase is described in *Pseudomonas* [[151](#_ENREF_151)], but not as a complex, but rather two separate activities AtzE and AtzF. These probably correspond to PFLU4022 and 4023. However, the AtzD gene upstream seem to be lacking.

### Riboflavin synthase / lumazine synthase

Suggested complex:

PFLU4750+5470+5472.

In contrast to *B. subtilis*, *E. coli* does not complex any of these [[152](#_ENREF_152)]; PFLU4750 and 5470 are RibH (riboflavin synthase), PFLU5472 is RibE (lumazine synthase).

# Hole-filling, iteration 1

Pathway Tools' algorithm for detection of metabolic 'holes' in the network lists the pathways with holes, the specific holes found in that pathway and – if possible – candidates to fill these holes for manual consideration. Below is listed the pathway holes (identified by either EC numer or complete raction) that were filled in the construction of *iSB1139*.

### β-alanine degradation I

**EC 1.2.1.18**: Rxn. not found in other bacteria , but inducible in *P. fluorescens* [[153](#_ENREF_153)]. Gene not known; choose top suggestion – methylmalonyl semialdehyde dehydrogenase (slightly altered substrate). Filled PFLU5203 and 0676.

### 2-nitropropane degradation

**EC 1.13.11.32**: The enzyme from *P. aeruginosa* has been structure elucidated[[154](#_ENREF_154)]. E-value is not very good in PathwayTools, but that’s most likely because only fungi are used. In BLAST, high similarity is achieved. Filled PFLU2972.

### 4-aminobutyrate degradation I

**EC 1.2.1.24**: GabD and GabD2 both give extremely high similarity. Both are listed as NADP+ dependent, not NAD+. But only GabD is colocalised with GabT. One enzyme with both cofactors recently described in *E. coli* [[155](#_ENREF_155)], and the gene is also found in *P. fluorescens* Pf-5 and PfO-1. Very difficult to get hold of sequence, but it seems very likely that GabD2 is the correct enzyme (from gene organisation). Filled PFLU1938.

### 4-aminobutyrate degradation II

**EC 1.4.1.2**: Only one real candidate, PFLU5326 – but this one is originally NADP-specific, whereas 1.4.1.2 uses NAD. PFLU5326 is GdhA, and seems strictly NADP-specific (BRENDA), but GdhB is characterized in *P. aeruginosa*. GdhB (<http://www.ncbi.nlm.nih.gov/entrez/viewer.fcgi?db=protein&id=12484094>) is NAD-specific[[156](#_ENREF_156)]. Searching by the initial amino acid sequence from *P. aeruginosa* reveals that PFLU3504 is the correct gene (extremely good match). Filled PFLU3504.

### 4-hydroxyproline degradation

**EC 4.1.3.16**: Top candidate is PFLU4836 – Eda. This rxn is catalysed by Eda in *E. coli*, so accept this.

### Acetate utilization and formation

**EC 2.7.2.1**: This enzyme (PurT) should be there – present in all other bacteria. Annotation has high confidence on PurT, so accept this. Plus: Only high-scoring candidate. Filled PFLU5017.

### Acetyl CoA fermentation to butyrate

**EC 4.2.1.55**: Top candidate, PFLU3030, is annotated as PaaF. However, the *paa* genes for styrene degradation are usually clustered. And the similarity matches are towards crotonyl in many cases. However, a BLAST reference for *P. putida* indicates aromatic substrates. PFLU3029 catalyses the next step in the pathway. Filled PFLU3030.

**EC 1.1.1.35**: No perfect candidate here; PFLU4661 and PFLU1553 are reasonable suggestions, but not clear. BLAST search on the sequence of PFLU1553 gives virtually perfect match to (exp verified) gene from *P. fragi*, FaoA, part of FaoAB complex for fatty acid oxidation. Also, FaoB is right downstream. Original paper not retrieved, but newer paper includes relevant results[[157](#_ENREF_157)]. α2β2 tetramer, but the α2 dimer exhibits 1.1.1.35 enzymatic activity alone. Synonym: FadB. Filled PFLU1553.

### ADP-L-glycero-β-D-*manno*-heptose biosynthesis

**[no EC number: D-sedoheptulose-7-P = D-α,β-D-heptose-7-P]:**

Only real candidate is PFLU0933 (DiaA) – annotated as DnaA initiator-associating protein in *E. coli* ; this is most likely wrong. Correct gene is almost certainly GmhA[[158](#_ENREF_158)] – exp. verified gene in *P. aeruginosa* is virtually identical. Exists as homotetramer. Filled PFLU0933.

**EC 5.1.3.20**: Only real candidate PFLU0483. Present in *E. coli* (RfaD), but not in Gram positive bacteria. Reaction is required for lipopolysaccharide biosynthesis. Just upstream is PFLU0482 encoding same function as RfaE in *E. coli*. Also called GmhD and HldD. Not possible to find papers here, but seems very likely that this is correct. Alignment against *E. coli* protein gives E=2e-12, with some regions well conserved. Filled PFLU0483.

### Allantoin degradation II

**EC 4.3.2.3**: Only one candidate, PFLU4362 (AllA). PFLU4361 and 4360 are annotated as related functions. Good match to ureidoglycolate hydrolase *E. coli*, but this rxn. gives NH_3_ and CO_2_, not urea – this function is already assigned to PFLU4362. However, there is a recent reference[[159](#_ENREF_159)] where it is cited that *Pseudomonas* generally use rxn 4.3.2.3 instead of 3.5.3.19 (as *E. coli*). Filled PFLU4362.

### Arginine degradation I (and VI)

**EC 2.6.1.13**: At least two good candidates (both annotated as ArgD); PFLU1624 and 4758. The latter is right upstream of other arginine utilisation genes. Both align very well in BLAST to a gene from *P. syringae*, but substrate there is N-acetyl-L-ornithine, not L-ornithine. From comparison with other *Pseudomonas* [[160](#_ENREF_160)] the PFLU4758 gene must be equal to AruC, with EC 2.6.1.13. Most likely, PFLU1624 fills same role. Note: The 2.6.1.13 used in the book includes a succinyl moiety that is used as a carrier. The ArgD gene is actually not identified explicitly. A paper that addresses the issued is found in[[140](#_ENREF_140)] – seems like AruC accepts acetylornithine as well, and that these two genes might be complementary inducible/repressible. Filled PFLU4758 *and* PFLU1624.

### Biosynthesis of 2’-(5’’-triphosphoribosyl)-3’dephospho-CoA

**EC 2.7.7.61**: Only real candidate is PFLU5742 – MdcB. Lies in Mdc (malonate decarboxylase) gene cluster elucidated in *P. putida[*[*161*](#_ENREF_161)*]* but very little is said about MdcB.

It seems that *P. fluorescens* MdcB is analogous to *K. pneumoniae* CitG[[162](#_ENREF_162)] (also good similarity, E=1e-68). The authors in this paper suggest that *P. putida* MdcG is analogous to the CitX that perform 2.7.7.61. A recent paper cites this function as known in *P. putida*. (Difficult to get reference on this, but it points to original article[[163](#_ENREF_163)].) MdcG performing EC 2.7.7.61 in *P. fluorescens* is then *gene PFLU5738*. Leave function 2.7.8.25 on PFLU5724. Assigned PFLU5738 to EC 2.7.7.61.

### Branched-chain α-keto acid dehydrogenase complex

**EC 2.3.1.168**: Suggested PFLU3966 (BkdB) – part of operon, and with related genes on each side. BLAST gives near perfect hit to exp. verified enzyme in *P. putida* [[44](#_ENREF_44)]. Filled with PFLU3966.

### Catechol degradation to β-ketoadipate

**EC 3.1.1.24**: Uncomplicated – lots of work has been done in *P. putida* [[164](#_ENREF_164)]. Filled PFLU1370 (PcaD).

### Citrulline biosynthesis

**EC 1.5.99.8**: Proline dehydrogenase. Only one real candidate – PFLU0451. Same gene (extremely good similarity) characterised in *P. putida* [[165](#_ENREF_165)]. Bifunctional – contains both 1.5.99.8 and 1.5.1.12. Filled PFLU0451.

### Cobalamin biosynthesis II (late cobalt incorporation)

**EC 1.16.8.1**: Only one real candidate – PFLU4489. Review of cobalamin biosynth in [[61](#_ENREF_61)]. The gene csp. to PFLU4489 is not well characterised – it is suggested[[61](#_ENREF_61)] that is it the cobalt reductase. It has decent sequence similarity (E=2e-40) to BluB from *S. melliloti[*[*63*](#_ENREF_63)*] –* but this gene is cited as involved in DMB formation. Bifunctional? From recent publications[[166](#_ENREF_166)] it seems possible that PFLU4489 is the correct gene, also called CobR. Still not entirely sure, but filled with PFLU4489.

**EC 2.1.1.131**: PFLU0607 – this is straight forward. CobJ[[61](#_ENREF_61)] (called CbiG in annotation). Filled with PFLU0607.

### Coenzyme A biosynthesis

**EC 2.7.1.33**: Only one real candidate – PFLU5542. This gene is annotated as a transcriptional regulator, but is really the type III pantothenate kinase described in other species[[167](#_ENREF_167)] (see supplemental material for the seq. of the *P. aeruginosa* homolog). Filled PFLU5542.

**EC 4.1.1.36**: Only one real candidate – PFLU5983 (CoaBC). This is straight forward from the annotation. Note that CoaA is not upstream – CoaX (PFLU5542, see above) does the job. Filled PFLU5983.

### D-galactarate degradation / D-glucarate degradation

[**no EC number: 5-keto-4-deoxy-D-glucarate = pyruvate + tartronate semialdehyde**]: PFLU3276 is assigned to very closely related rxn. – depending on whether this is HpcH or DDGA[[168](#_ENREF_168)]. Fill PFLU3276.

### Ethylene glycol degradation

[**no EC number: NAD+ + ethylene glycol = glycoaldehyde + NADH**]:

Alcohol dehydrogenases are generally promiscuous, and it is difficult to find any specific info about enzymes catalysing this particular rxn. PFLU1412 (best candidate) is already catalysing various 1.1.1.1-rxns (with other alcohols). Filled with PFLU1412.

### Fatty acid elongation – unsaturated II

**EC 4.2.1.60**: Top candidate PFLU1836 (FabA) is already catalysing a variant of 4.2.1.60, and aligns very well. Filled PFLU1836.

[**no EC number: trans-Δ2-decenoyl-ACP = cisΔ3-decenoyl-ACP**]:

Only real candidate PFLU1836 – FabA. FabA is known to be both a dehydratase and isomerase in *P. aeruginosa* [[169](#_ENREF_169)] – virtually identical by BLAST. Filled PFLU1836.

[**no EC number: NADPH+β-keto-cis-Δ5-dodecenoyl-ACP = NADP+ + β-hydroxy-cis-Δ5-dodecenoyl-ACP**]:

PFLU4705 (FabG) performs this, and there is no reason to doubt annotation here. Filled PFLU4705.

### Fatty acid β-oxidation I

**EC 5.3.3.8**: Top candidate has this EC listed, but under the /similarity tag, so it’s not registered in PathwayTools. Enz. activities of the FadAB / FaoAB complex: 4.2.1.17, 5.3.3.8, 1.1.1.35 and 5.1.2.3. The complex from *P. fragi* has been functionally characterised[[157](#_ENREF_157)], and here EC 2.3.1.16 is also included with the complex. Filled PFLU1553.

### Flavin biosynthesis

**EC 3.5.4.26**: In all organisms investigated, this function is in the same protein as 1.1.1.193, and *P. fluorescens* annotation also cites it as bifunctional. Filled PFLU5473.

**EC 2.7.7.2**: Only one real candidate, PFLU0767. Annotated as RibF (EC 2.7.1.26), with both enz. functions in annotation. Filled PFLU0767.

### Formaldehyde oxidation II (glutathione-dependent)

**EC 3.1.2.12**: Only one real candidate; PFLU1296. High similarity to *E. coli* YeiG, recently characterised[[170](#_ENREF_170)]. No bacterial enz. was char. before this. One other enz. with same function but higher specificity is described; FrmB. Inverse BLAST using the *E. coli* aa seq. against *P. fluorescens* genome yields the same (!) gene PFLU1296 as homologue for FrmB too with very good similarity. So probably, *P. fluorescens* has only one paralogue. Filled PFLU1296.

### FormylTHF biosynthesis I

**EC 6.3.2.17**: One candidate; PFLU4186, already connected to 6.3.2.12. These two EC are catalysed by the same enzyme in *E. coli* (FolC, very good similarity), so this is straight forward. Filled PFLU4186.

### Fructose degradation to pyruvate and lactate (anaerobic)

**EC 2.7.1.69**: This rxn is expected to be only in *Firmicutes*, and there is an alternative pwy in *P. fluorescens* (2.7.1.4, phosphorylation by ATP). Two candidate systems; PFLU0804–0806 (FruRAKB) and PFLU5027–28. The former most confident – the latter annotated as glucose-specific. Actually, PFLU0804 seems to be the fructose-specific PTS component. PTS system for fructose is described in *P. putida[*[*171*](#_ENREF_171)*]*, and similarity is virtually perfect. Filled PFLU0804.

### GDP-D-rhamnose biosynthesis

**EC 1.1.1.187**: Described in *P. aeruginosa* [[172](#_ENREF_172)]. The top candidate from PathwayTools, PFLU3667 is annotated as GDP-fucose synthetase, but alignment in BLAST of the two does not yield very good results. Bl2seq yields best hit against 3^rd^ candidate in PathwayTools, PFLU0483. Quite good similarity (E=2e-19) and conserved residues are conserved here too[[172](#_ENREF_172)]. Also, it is placed near other lipopolysaccharide genes. Filled PFLU0483.

### GDP-mannose metabolism

**EC 5.3.1.8**: This rxn is the AlgA rxn. and PathwayTools suggest PFLU0979 correctly. Filled PFLU0979.

### Gluconeogenesis

**EC 1.1.1.40**: The NADP-dependent variant of malic enzyme. Not a lot of papers on this, but one[[173](#_ENREF_173)] that describes two different forms from *E. coli*. Using the *E. coli* MaeB to BLAST *P. fluorescens* genome yields very good hit to PFLU0405 (same as PathwayTools suggest). Filled PFLU0405.

**EC 1.1.1.37**: Malate dehydrogenase Mdh. BLASTing with the *E. coli* enzyme does not give good hit in *P. fluorescens* genome. 1.1.1.37 already analysed (see above), and concluded that we **can not fill**. By alignment, most of the key residues are conserved between *E. coli* and the *P. fluorescens* candidate (PFLU2704), but not all. Apparently, this enzyme is characterised by large variability in primary amino acid sequence[[174](#_ENREF_174)]. NB! There is a paper on NAD/NADP cycling in *P. fluorescens* [[175](#_ENREF_175)] but it contains no sequence info of any kind.

### Glucose degradation (oxidative)

**EC 1.1.99.3**: Best candidate is PFLU0052. BLASTing with this gives extremely good hit (E=0) to gluconate 2-dehydrogenase from *Pectobacterium cypripedii* (tidl. *Erwinia cyp.*). Experimentally verified[[176](#_ENREF_176)], GenBank acc.no. U97665. By BLASTing against *P. fluorescens* genome, PFLU0051 is the homologue of subunit III, and PFLU0053 (AdhB) is the homologue of the last subunit. Filled PFLU0052.

**EC 1.1.1.43 and 2.7.1.13**: Described in[[177](#_ENREF_177)] from *P. aeruginosa*. This describes the *kgu* operon for 2-ketogluconate utilisation. GenBank acc.no. AF012100.

Homologues (extremely high similarity) by BLASTing *P. fluorescens* genome: KguK = PFLU2714, KguE =PFLU2715, PtxS = PFLU2716 so can be assumed that that KguT=PFLU2714, KguD=2713. The enzymes have not been biochemically characterised in detail, but are shown to be necessary for growth on 2-ketogluconate, and authors do a nice job on trying to elucidate functions based on sequence. Filled 2.7.1.13 with PFLU2714 and 1.1.1.43 with PFLU2713.

### Glutamate degradation IV

**EC 1.2.1.24**: Described in recent paper[[155](#_ENREF_155)]. The “real” GabD is very likely to be PFLU0180, as GabT is just downstream. That means PFLU1938 is not really GabD as annotated, but probably YneI. GabD is NADP+-dependent, whereas YneI is NAD(P)+. By BLASTing *P. fluorescens* genome with *E. coli* YneI seq., the *P. fluorescens* homologue is actually found to be PFLU4212. E=1e-169, i.e. very good match (and much better than next-best).

This means: PFLU0180 and PFLU1938 are both GabD (NADP+, EC 1.2.1.16), whereas PFLU4212 is YneI (NAD(P)+, EC 1.2.1.24).

### Glycine betaine degradation

**EC 1.5.99.2**: Not much published about this in bacteria in general, but one very recent paper from *P. aeruginosa* [[178](#_ENREF_178)] describes the whole pathway. 1.5.99.2 is catalysed by newfound DgcAB complex; PA5398–5399 = PFLU5664–5663. Filled PFLU5664 and PFLU5663.

**EC 2.1.1.5**: Not sure if the cofactors (L-homocysteine) are correct – not investigated in the paper[[178](#_ENREF_178)] as far as I can see. But the glycine betaine demethylase GbcAB performs the transformation of glycine betaine. PA5410–PA5411 in *P. aeruginosa*, equals (GbcAB) PFLU5660–PFLU5659. Filled PFLU5660–5659.

### Glycogen degradation

**EC 3.2.1.33**: One real candidate, PFLU3369 (GlgX). Seems like this is the eukaryotic version; EC 3.2.1.68 is the one in bacteria. On the other hand, 3.2.1.- is also used, so this is probably a minor imprecision. Very good similarity with *E. coli* enzyme GlgX (E=e-162) exp. verified[[179](#_ENREF_179)], so function OK. Filled PFLU3369.

**EC 2.4.1.25**: One candidate; PFLU3366. High similarity to *E. coli* MalQ, which in well characterized. Filled PFLU3366.

### Histidine biosynthesis I

**EC 3.1.3.15**: Best candidate PFLU0327. This is a bifunctional enzyme (HisB) catalyzing both 4.2.1.19 and 3.1.3.15. Filled PFLU0327.

### KDO transfer to lipid IV_A_

[**EC 2.-.-.-: KDO-lipidIV 🡪 KDO2-lipidIV**]: Both rxns. in this pwy are catalysed by same enzyme in all other species (WaaA/KdtA in *E. coli*); same moiety is attached twice to the lipid core[[180](#_ENREF_180)]. Only one candidate in PathwayTools; PFLU0490. Filled PFLU0490.

### KDO_2_-lipid A biosynthesis I

[**EC 2.3.1.-: KDO2-(lauroyl)-lipid IVA 🡪 KDO2-lipid A**]: This is a little bit tricky; the suggested rxn. in PathwayTools adds myristoyl moieties to lipid IV, but this is cited as not possible in *P. aeruginosa[*[*181*](#_ENREF_181)*]*; it can only use lauroyl-ACP. Filled PFLU4368.

### Ketogluconate metabolism

Some ambiguity exists here; 2-keto-G-gluconate is sometimes considered synonym to 2-keto-L-gulonate. This is not correct; the formulas (from Sigma) show different stereochemistry (gulonate left, gluconate right):


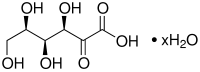

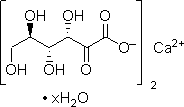


It is not easy to find out whether these two can serve as alternative substrates. It actually seems like some enzymes can utilize both (like YiaJ from *E. coli* [[182](#_ENREF_182)]), but the products are different; idonate from 2-keto-L-gulonate and gluconate from 2-keto-D-gluconate. Try to sort this out:

| *E. coli* gene name | YqhE | YafB | YiaE | YjgU / idnO |
| --- | --- | --- | --- | --- |
| *P. fluorescens* gene | PFLU2183 (E=8e-42) | PFLU2183 (E=e‑104) | PFLU2712 (E=4e‑89)  PFLU0968 (E=6e‑74) | PFLU4705 (E=2e‑36)  PFLU2089 (E=2e‑35) |
| Enz. name | 25DKGR-A | 25DKGR-B | 2KR | 5KDGR |
| EC | 1.1.1.274* | 1.1.1.274* | 1.1.1.215 | 1.1.1.69 |
| Rxn 1 | 25DKG 🡪 2KLG | 25DKG 🡪 2KLG | 25DKG 🡪 5KDG | 5KDG 🡪 D-gluconate |
| Rxn 2 |  |  | 2KLG 🡪 IA |  |
| Rxn 3 |  |  | 2KDG 🡪 D-gluconate |  |

Abbreviations are ref. to [[182](#_ENREF_182)].

*P. fluorescens* genes based on best hit when BLASTing *P. fluorescens* genome with *E. coli* homologue

*BRENDA also cites the product here as 2KDG. Error?

**EC 1.1.1.264**: Not a lot described, but IdnD from *E. coli* [[183](#_ENREF_183)] used in BLASTing *P. fluorescens* genome yields best hit in PFLU3993 (E=e-21, short protein). Filled PFLU3993.

[**EC 1.1.1.-: 2-dehydro-D-gluconate + NADPH = L-idonate + NADP+**]:

This rxn is catalysed by the same enzyme as 1.1.1.215 in *E. coli* (YiaE/GhrB). That EC is assigned to PFLU0968, which is also top candidate in PathwayTools. But PFLU2712 has higher seq. similarity isolated. Quite some work has been done in *E. coli* [[182](#_ENREF_182)]. Filled PFLU2712.

**EC 1.1.1.69**: Lots of short-chain dehydrogenases here. Best inverse BLAST hits on PFLU4705 (FabG) and PFLU2089, but one of the suggestions in PathwayTools, PFLU3991 with good E-value is right downstream of a GntK gene and PFLU3993 which encodes L-idonate 5-dehydrogenase, an upstream step in pwy. Note: PFLU3729 gives higher homology, and is located after ribose transporters. Filled PFLU3991.

### Lysine biosynthesis I

**EC 2.6.1.17**: Catalysed by bifunctional ArgD (/DapC), PFLU4758. An isozyme in PFLU1624 – also ArgD. By inverse BLASTing with the *E. coli* ArgM (AstC) isozyme, however, it turns out that PFLU4758 should be ArgM/AstC[[184](#_ENREF_184)], not ArgD (E=e-146, extremely good match). AstC prefers succinylornithine, but can also use acetylornithine. Note also that ArgD is involved in arginine biosynthesis, whereas AstC is involved in degradation. As AstC can complement ArgD functionally[[184](#_ENREF_184)], is should possess succinyl-DAP substrate acceptance too. Filled PFLU1624 and PFLU4758.

### Methylcitrate cycle

**EC 4.2.1.99**: Only one real candidate in PathwayTools; PFLU4628, 2-methylcitrate dehydratase. This is a bit tricky: In the normal TCA cycle it seems like conversion from citrate via cis-aconitate to isocitrate (incl. dehydrataion – rehydration) is catalysed by the same enzyme; *aconitase*. That is probably why PathwayTools has assumed the same for the methylcitrate cycle. But this is not cited as correct; in *Ralstonia eutropha* [[185](#_ENREF_185)] (very closely related to Pseudomonas) detailed analysis indicated either that “ORF5” (csp. PFLU4629) and AcnM (csp. PFLU4630) catalyse dehydration and rehydration, respectively, or that they perform both in a complex. BLASTing *P. fluorescens* with *R. eutropha* AcnM gives perfect hit to PFLU4630. Gene organisation is also conserved. That means this one annotation should be updated, and the PrpD protein (PFLU4628) should also be re-annotated; it is not essential for methylcitrate conversion.

Assigned both dehydration and rehydration (4.2.1.79 and 4.2.1.99) to both PFLU4629 and PFLU4630.

**EC 4.1.3.30**: Only one real candidate, PFLU4632 (PrpB). Very good similarity to exp.verified *R. eutropha* PrpB[[185](#_ENREF_185)]. Filled PFLU4632**.**

### Octane oxidation

**EC 1.14.15.3**: Only one real candidate, PFLU3535. BLAST gives good hit (e-82) to *Pseudomonas oleovorans* (exp. verified, AlkB). Filled PFLU3535.

### Peptidoglycan biosynthesis I

**EC 5.1.1.3**: Glutamate racemase. Lots of hits in PathwayTools with very low E-value, but most of them in NRPS clusters or pyoverdin biosynthesis. Only candidate left after exclusion of these is PFLU0741. BLASTing with *E. coli* MurI gives same hit (E=e-35, short protein), so this is OK. Filled PFLU0741.

### Peptidoglycan biosynthesis II

**EC 2.4.1.129**: One paper describes Mra/Mur genes in *P. aeruginosa* [[186](#_ENREF_186)]; seems identical in *P. fluorescens*. Best hit (E-value) is actually against PFLU5223, which catalyses this rxn in *E. coli*. PFLU0941 (in the *mur* cluster) is not as well characterised. Filled PFLU5223.

**EC 6.3.2.7**: MurE protein, csp. to PFLU0942[[186](#_ENREF_186)]. Filled PFLU0942.

### ppGpp biosynthesis

**EC 3.6.1.40**: One real candidate; PFLU5911. Annotated as Ppx – exopolyphosphatase. The highly similar (e-97) protein from *E. coli* has both activities[[187](#_ENREF_187)], even though there exists two separate enzymes in *E. coli*. Searching *P. fluorescens* genome with *E. coli* GppA gave only one his, so this might be the only one in *P. fluorescens*. Filled PFLU5911.

### Proline degradation II

**EC 1.5.99.8**: One real candidate; PFLU0451. This is a bifunctional protein with both 1.5.99.8 and 1.5.1.12 in the annotation. Filled PFLU0451.

### Purine degradation

Note that this pwy is only expected in plants, even though most of the rxns are present in *P. fluorescens*.

**EC 3.1.3.5**: One good candidate; SurE, PFLU1299. Annotation is confident that this is SurE, and EC 3.1.3.5 is certainly catalysed by this enzyme[[188](#_ENREF_188)]. Filled PFLU1299.

### Purine nucleotides *de novo* biosynthesis I

A very recent review on purine biosynth. has been published[[189](#_ENREF_189)].

[**no EC number: NDP 🡪 dNDP**]: One real candidate, PFLU4726, subunit (with PFLU4768) of ribonucleoside-PP reductase, NrdAB.

Seems like the whole story of ribnucleotide reductases (3 classes) is a bit complicated. A recent paper from *P. aeruginosa* [[190](#_ENREF_190)] is very informative. From the PathwayTools rxn., it seems that we’re looking for a type Ib RNR, NrdEF+NrdH, but according to the paper, only type Ia is found in *P. aeruginosa*. Only hit when BLASTing *P. fluorescens* genome with *E. coli* NrdE, is NrdA (semi-good match). 🡪 Rxn probably not present in *P. fluorescens*.

**EC 6.3.4.18**: In *E. coli*, this is PurK. In *P. fluorescens*, top candidate is PFLU6054, subunit together with PurE of P-ribosylaminoimidazole carboxylase. These rxns. have been elucidated in *E. coli* [[191](#_ENREF_191)]. Filled PFLU6054.

**EC 6.3.4.1**: Only one real candidate; PFLU5043 (GuaA). Very good similarity to *E. coli* enzyme[[192](#_ENREF_192)]. Filled PFLU5043.

**EC 2.1.2.3**: One real candidate; PFLU0612 (PurH; bifunctional EC 2.1.2.3 + 3.5.4.10)[[189](#_ENREF_189)]. Filled PFLU0612.

### Salvage pathways of adenine, hypoxanthine, and their nucleosides

[**no EC: hypoxanthine + H2O = 2H^+^ + xanthine**]: Xanthine dehydrogenase. XdhAB annotated in *P. fluorescens* genome. In *E. coli*, they complex with XdhC, but *e.g.* in *Rhodobacter*, the comples is only XdhAB. There are indications that PFLU4594 is XdhC – an accessory protein. Filled PFLU4592.

### Sulfate reduction I (assimilatory)

**EC 1.8.1.2**: Very good E-value for PFLU3426, but PFLU2657 is CysI. This protein performs the rxn. in *E. coli*. An article regarding *P. putida* [[193](#_ENREF_193)] describes that CysI has this function, so this is probably OK. Filled PFLU2657.

### Thiamine biosynthesis

A recent review [[194](#_ENREF_194)] is very useful here.

**EC 2.7.1.49**: Top candidate is PFLU5399, annotated as “conserved hypothetical protein”. This is almost surely ThiD – don’t know why annotation is strange here. ThiE is downstream, as described in [[194](#_ENREF_194)]. BLASTing with *E. coli* ThiD identifies PFLU5399 too. Filled PFLU5399.

**EC 2.7.4.7**: Also ThiD (PFLU5399) – a bifunctional enzyme[[194](#_ENREF_194)]. Filled PFLU5399. The TenA protein is probably associated to ThiD, but function is unknown[[194](#_ENREF_194)].

### Trehalose biosynthesis V

**EC 3.2.1.68**: Only one really high-soring candidate; GlgX (PFLU3369). The enzyme with this EC has been characterised from another *Pseudomonas* [[195](#_ENREF_195)], and BLASTing with this enzyme gives good hit against PFLU3369. Filled PFLU3369.

### Trehalose degradation I (low osmolarity)

[**no EC number, trehalose phosphotransferase system**]: This transport is characterised in another strain of *P. fluorescens* [[196](#_ENREF_196)]; TreP, the transporter, is identical to PFLU5040, which is originally annotated as ScrA (sucrose-specific). This is also the only uptake system for trehalose. Filled PFLU5040.

### Tryptophan biosynthesis

[**no EC number; indole-3-glycerol phosphate 🡪 indole**]: Filled PFLU0035+0036 (good candidate). Rxn. described in old paper from *E. coli* [[197](#_ENREF_197)].

### Ubiquinone biosynthesis I (aerobic)

The ubiquinone biosynthesis in microbes is reviewed in [[198](#_ENREF_198)]. The pathway is well conserved, so can transfer this info directly to *P. fluorescens*.

Bifunctional enzyme UbiG (strong similarity to *E. coli*). Filled PFLU1639.

UbiE – good similarity to *E. coli.* Filled PFLU0389.

UbiF. Good similarity to top PathwayTools candidate PFLU5877, even if this one isn’t formally annotated as UbiF. Filled PFLU5877.

### UDP-*N*-acetyl-D-glucosamine biosynthesis

**EC 2.7.7.23**: Clearlycatalyzed by bifunctional protein GlmU. Filled PFLU6116.

### UDP-glucose conversion

**EC 5.1.3.2**: The enzyme (GalE) is not marked in *E. coli* (PathwayTools), but searching with the *Vibrio vulnificus* GalE [[199](#_ENREF_199)] yields same hit as PathwayTools; PFLU0483. Annotation also indicates that this could be the correct gene. BLASTing with the WspP protein from *P. aeruginosa* [[200](#_ENREF_200), [201](#_ENREF_201)] gives good hit in the same gene. This protein, however, is found to prefer the N-acetylated sugars as substrate, even though it will process the unacetylated. Filled PFLU0483.

### Valine degradation I

**EC 3.1.2.4**: There is a paper on this enzyme from *P. aeruginosa* [[202](#_ENREF_202)] but it’s not possible to find any reference to where they found the gene. Searching with the human protein gives two OK hits: PFLU1440 (E=8e-36, 28 % identity) and PFLU3032 (E=3e-49, 33 % identity). This is pretty significant. So can’t find any direct evidence, but seems likely. Filled PFLU3032. Also, this gene is placed in immediate vicinity of other related genes.

# Pathway HoleFiller – iteration 2

After metabolic 'holes' in the network were filled in iteration 1, the presence of biochemical pathways in the model was re-assessed (by Pathway Tools), and based on this, new 'holes' were identified. These were filled as described below.

### 4-hydroxymandelate degradation

**EC 1.2.1.28**: PFLU3306 has very good similarity to *P. putida* XylC. Filled PFLU3306.

### Acetyl-CoA fermentation to butyrate II

**EC 1.1.1.36**: Lots of candidates, but one stands out on E-value; PFLU4705. This is FabG, which is an 3-oxoacyl-ACP-reductase, so not likely the correct one. But PathwayTools top candidate PFLU3027 sits next to genes that seem related; PFLU3208 is putative acetyl-CoA acetyltransferase, PFLU3209 is Bcd2, butyryl-CoA dehydrogenase, PFLU3030 is crotonyl-CoA hydratase. The three first genes seem like an operon too. Filled PFLU3207.

**EC 2.8.3.8**: The enzyme is not very well described. Best hit (only real hit) is succinyl-CoA:3-ketoacid-CoA transferase complex (ScoAB, PFLU2151-2152). PFLU2150 is an acetyl-CoA acetyltransferase. ScoAB already has different substrates – filled PFLU2151.

### Adenosylcobalamin biosynthesis II (late cobalt incorporation)[[60](#_ENREF_60), [61](#_ENREF_61)]

**EC 2.1.1.107**: Two very good candidates; PFLU3796 (CysG) and PFLU4611 – both very low E-value. PFLU4611 is the CobA gene – clearly highest similarity to *P. denitrificans* gene, followed by PFLU3796. The 2.1.1.107 activity is supposed to exist in both[[203](#_ENREF_203)]. Filled PFLU3976 and PFLU4611.

### Allantion degradation II / III

**EC 3.5.2.5**: Recent paper[[204](#_ENREF_204)] deals with allantoinase from *P. fluorescens* and shows that the PuuE protein performs this activity. PuuE is PFLU4359 (annotated as “conserved hypothetical protein”) with perfect match. Filled PFLU4359.

### Fatty acid elongation – unsaturated

**EC 5.3.3.14**: Only one real (very good) candidate – FabA (is 4.2.1.60). These EC are performed by same enz. in *E. coli* too. Filled PFLU1836.

[**no EC number; β-hydroxyacyl-ACP dehydratase**]: *E. coli* FabA and FabZ has good similarity. Filled PFLU1281 and PFLU1836.

### Fatty acid β-oxidation I

**EC 5.3.3.8**: FadA / FaoA. Very good match (*E. coli*) to PFLU1553. Also very good match to PFLU4661, but this might be a separate gene cluster. Filled PFLU1553.

**EC 1.1.1.35**: Same as above (same enzymes match). Filled PFLU1553.

### Folate polyglutamylation I

**EC 6.3.2.17**: Only one candidate (very good) – FolC in annotation, this is also the enzyme in *E. coli*. Filled PFLU4186.

[**no EC number: glycine + THF + NAD+ = ammonia + CO2 + 5,10-methylene-THF + NADH**]: Good sequence similarity to glycine cleavage system for *E. coli* – this is a complex. Adds the function to members of two gcv complexes. Filled PFLU4897 and PFLU5874.

### Formate to nitrate electron transfer

**EC 1.2.1.2**: One (good) candidate – PFLU3426. Located downstream of *nirB* (nitrate-related). Filled PFLU3426.

### Histidine degradation I

**EC 3.5.3.8**: One (good) candidate; PFLU4510. Note that this pathway is not marked present in *E. coli*, but exists in *Vibrio cholerae* and *A. tumefaciens* ( plus Gram+ organisms). PFLU4510 is already annotated as agmatinase in SBW25.

NB! It turns out that *Pseudomonas* uses another pathway; Histidine degradation II with an extra step[[205](#_ENREF_205)]. The two terminal enzymes, HutF and HutG, are EC 3.5.3.13 and 3.5.1.68.

Searching with HutG from *P. putida* KT2440 gives hit only against PFLU0370 (e=-113). This is immediately downstream of HutI, with organisation same as in *P. putida*.

Searching with HutF yields PFLU0358 as only hit – this is again in accordance with organisation in *P. putida*. Assigned EC 3.5.3.13 to PFLU0358 and EC 3.5.1.68 to PFLU0370.

### Siroheme biosynthesis

**EC 4.99.1.4 (and 1.3.1.76)**: In *E. coli*, all four rxns in this pwy is catalysed by same enzyme CysG. Also, both candidates in PathwayTools catalyse the other rxns. Filled both ECs with both PFLU3796 and PFLU4611.

### Trehalose biosynthesis V

**EC 5.4.99.15**: Only one real candidate, PFLU3367. Surrounding genes fit well, functionally. Filled PFLU3367.

### Valine degradation I

**EC 1.2.1.25**: Not present in a lot of databases, but is described as very well known in other *Pseudomonas[*[*206*](#_ENREF_206)*]*. Filled PFLU3964–3967.

# Pathway Holefiller – Iteration 3

After metabolic 'holes' in the network were filled in iteration 2, the presence of biochemical pathways in the model was re-assessed (by Pathway Tools), and based on this, new 'holes' were identified. These were filled as described below. The subsequent iteration 4 did not identify any new pathways or holes.

### Tetrahydrofolate biosynthesis II

**EC 6.3.2.17**: One very good candidate, also by comparison with *E. coli*. Filled PFLU4186.

### Uridine-5’-phosphate biosynthesis

**EC 1.3.5.2**: Sequence similarity with other organisms in Pathway Tools gives very good candidate. Filled PFLU4603.

# References

1. Gutsche, J., U. Remminghorst, and B.H. Rehm, *Biochemical analysis of alginate biosynthesis protein AlgX from Pseudomonas aeruginosa: purification of an AlgX-MucD (AlgY) protein complex.* Biochimie, 2006. **88**(3-4): p. 245-51.

2. Franklin, M.J., S.A. Douthit, and M.A. McClure, *Evidence that the algI/algJ gene cassette, required for O acetylation of Pseudomonas aeruginosa alginate, evolved by lateral gene transfer.* J Bacteriol, 2004. **186**(14): p. 4759-73.

3. Franklin, M.J. and D.E. Ohman, *Mutant analysis and cellular localization of the AlgI, AlgJ, and AlgF proteins required for O acetylation of alginate in Pseudomonas aeruginosa.* J Bacteriol, 2002. **184**(11): p. 3000-7.

4. Remminghorst, U. and B.H. Rehm, *In vitro alginate polymerization and the functional role of Alg8 in alginate production by Pseudomonas aeruginosa.* Appl Environ Microbiol, 2006. **72**(1): p. 298-305.

5. Remminghorst, U. and B.H. Rehm, *Alg44, a unique protein required for alginate biosynthesis in Pseudomonas aeruginosa.* FEBS Lett, 2006. **580**(16): p. 3883-8.

6. Amikam, D. and M.Y. Galperin, *PilZ domain is part of the bacterial c-di-GMP binding protein.* Bioinformatics, 2006. **22**(1): p. 3-6.

7. Bardey, V., et al., *Characterization of the molecular mechanisms involved in the differential production of erythrose-4-phosphate dehydrogenase, 3-phosphoglycerate kinase and class II fructose-1,6-bisphosphate aldolase in Escherichia coli.* Mol Microbiol, 2005. **57**(5): p. 1265-87.

8. Bell, B.J., et al., *Structure of 2-keto-3-deoxy-6-phosphogluconate (KDPG) aldolase from Pseudomonas putida.* Acta Crystallogr D Biol Crystallogr, 2003. **59**(Pt 8): p. 1454-8.

9. Ahn, C., J. Byun, and J. Yim, *Purification, cloning, and functional expression of dihydroneopterin triphosphate 2'-epimerase from Escherichia coli.* J Biol Chem, 1997. **272**(24): p. 15323-8.

10. Haussmann, C., et al., *Biosynthesis of pteridines in Escherichia coli. Structural and mechanistic similarity of dihydroneopterin-triphosphate epimerase and dihydroneopterin aldolase.* J Biol Chem, 1998. **273**(28): p. 17418-24.

11. Hashimoto, Y., M. Yamashita, and Y. Murooka, *The Propionibacterium freudenreichii hemYHBXRL gene cluster, which encodes enzymes and a regulator involved in the biosynthetic pathway from glutamate to protoheme.* Appl Microbiol Biotechnol, 1997. **47**(4): p. 385-92.

12. Shen, B.W., et al., *The crystal structure of a bacterial, bifunctional 5,10 methylene-tetrahydrofolate dehydrogenase/cyclohydrolase.* Protein Sci, 1999. **8**(6): p. 1342-9.

13. Kneidinger, B., et al., *Biosynthesis pathway of ADP-L-glycero-beta-D-manno-heptose in Escherichia coli.* J Bacteriol, 2002. **184**(2): p. 363-9.

14. McArthur, F., et al., *Functional analysis of the glycero-manno-heptose 7-phosphate kinase domain from the bifunctional HldE protein, which is involved in ADP-L-glycero-D-manno-heptose biosynthesis.* J Bacteriol, 2005. **187**(15): p. 5292-300.

15. Bessman, M.J., et al., *The gene ygdP, associated with the invasiveness of Escherichia coli K1, designates a Nudix hydrolase, Orf176, active on adenosine (5')-pentaphospho-(5')-adenosine (Ap5A).* J Biol Chem, 2001. **276**(41): p. 37834-8.

16. Hand, N.J. and T.J. Silhavy, *Null mutations in a Nudix gene, ygdP, implicate an alarmone response in a novel suppression of hybrid jamming.* J Bacteriol, 2003. **185**(22): p. 6530-9.

17. Hoffmann, N., et al., *Biochemical characterization of the Pseudomonas putida 3-hydroxyacyl ACP:CoA transacylase, which diverts intermediates of fatty acid de novo biosynthesis.* J Biol Chem, 2002. **277**(45): p. 42926-36.

18. Rehm, B.H., N. Kruger, and A. Steinbuchel, *A new metabolic link between fatty acid de novo synthesis and polyhydroxyalkanoic acid synthesis. The PHAG gene from Pseudomonas putida KT2440 encodes a 3-hydroxyacyl-acyl carrier protein-coenzyme a transferase.* J Biol Chem, 1998. **273**(37): p. 24044-51.

19. Forster-Fromme, K., et al., *Identification of genes and proteins necessary for catabolism of acyclic terpenes and leucine/isovalerate in Pseudomonas aeruginosa.* Appl Environ Microbiol, 2006. **72**(7): p. 4819-28.

20. Calhoun, M.W., J.W. Thomas, and R.B. Gennis, *The cytochrome oxidase superfamily of redox-driven proton pumps.* Trends Biochem Sci, 1994. **19**(8): p. 325-30.

21. Cotter, P.A., et al., *Cytochrome o (cyoABCDE) and d (cydAB) oxidase gene expression in Escherichia coli is regulated by oxygen, pH, and the fnr gene product.* J Bacteriol, 1990. **172**(11): p. 6333-8.

22. Au, D.C. and R.B. Gennis, *Cloning of the cyo locus encoding the cytochrome o terminal oxidase complex of Escherichia coli.* J Bacteriol, 1987. **169**(7): p. 3237-42.

23. Matsushita, K., et al., *Reconstitution of active transport in proteoliposomes containing cytochrome o oxidase and lac carrier protein purified from Escherichia coli.* Proc Natl Acad Sci U S A, 1983. **80**(16): p. 4889-93.

24. Matsushita, K., L. Patel, and H.R. Kaback, *Cytochrome o type oxidase from Escherichia coli. Characterization of the enzyme and mechanism of electrochemical proton gradient generation.* Biochemistry, 1984. **23**(20): p. 4703-14.

25. Alonso, J.M. and A. Garrido-Pertierra, *Kinetic properties of 5-carboxymethyl-2-hydroxymuconate semialdehyde dehydrogenase from Escherichia coli.* Biochimie, 1986. **68**(5): p. 731-7.

26. Prieto, M.A., E. Diaz, and J.L. Garcia, *Molecular characterization of the 4-hydroxyphenylacetate catabolic pathway of Escherichia coli W: engineering a mobile aromatic degradative cluster.* J Bacteriol, 1996. **178**(1): p. 111-20.

27. Roper, D.I., J.M. Stringfellow, and R.A. Cooper, *Sequence of the hpcC and hpcG genes of the meta-fission homoprotocatechuic acid pathway of Escherichia coli C: nearly 40% amino-acid identity with the analogous enzymes of the catechol pathway.* Gene, 1995. **156**(1): p. 47-51.

28. Izumi, A., et al., *Structure and Mechanism of HpcG, a Hydratase in the Homoprotocatechuate Degradation Pathway of Escherichia coli.* J Mol Biol, 2007. **370**(5): p. 899-911.

29. Jenkins, J.R. and R.A. Cooper, *Molecular cloning, expression, and analysis of the genes of the homoprotocatechuate catabolic pathway of Escherichia coli C.* J Bacteriol, 1988. **170**(11): p. 5317-24.

30. Stringfellow, J.M., B. Turpin, and R.A. Cooper, *Sequence of the Escherichia coli C homoprotocatechuic acid degradative operon completed with that of the 2,4-dihydroxyhept-2-ene-1,7-dioic acid aldolase-encoding gene (hpcH).* Gene, 1995. **166**(1): p. 73-6.

31. Chalker, A.F., et al., *Systematic identification of selective essential genes in Helicobacter pylori by genome prioritization and allelic replacement mutagenesis.* J Bacteriol, 2001. **183**(4): p. 1259-68.

32. Lee, J., et al., *An Alternative Polyamine Biosynthetic Pathway Is Widespread in Bacteria and Essential for Biofilm Formation in Vibrio cholerae.* Journal of Biological Chemistry, 2009. **284**(15): p. 9899-9907.

33. Campbell, J.W. and J.E. Cronan, Jr., *The enigmatic Escherichia coli fadE gene is yafH.* J Bacteriol, 2002. **184**(13): p. 3759-64.

34. McMahon, B., M.E. Gallagher, and S.G. Mayhew, *The protein coded by the PP2216 gene of Pseudomonas putida KT2440 is an acyl-CoA dehydrogenase that oxidises only short-chain aliphatic substrates.* FEMS Microbiol Lett, 2005. **250**(1): p. 121-7.

35. Akochy, P.M., et al., *Direct glutaminyl-tRNA biosynthesis and indirect asparaginyl-tRNA biosynthesis in Pseudomonas aeruginosa PAO1.* J Bacteriol, 2004. **186**(3): p. 767-76.

36. Best, E.A. and V.C. Knauf, *Organization and nucleotide sequences of the genes encoding the biotin carboxyl carrier protein and biotin carboxylase protein of Pseudomonas aeruginosa acetyl coenzyme A carboxylase.* J Bacteriol, 1993. **175**(21): p. 6881-9.

37. Pitcher, R.S., M.R. Cheesman, and N.J. Watmough, *Molecular and spectroscopic analysis of the cytochrome cbb(3) oxidase from Pseudomonas stutzeri.* J Biol Chem, 2002. **277**(35): p. 31474-83.

38. Oh, J.I. and S. Kaplan, *Oxygen adaptation. The role of the CcoQ subunit of the cbb3 cytochrome c oxidase of Rhodobacter sphaeroides 2.4.1.* J Biol Chem, 2002. **277**(18): p. 16220-8.

39. Comolli, J.C. and T.J. Donohue, *Differences in two Pseudomonas aeruginosa cbb3 cytochrome oxidases.* Mol Microbiol, 2004. **51**(4): p. 1193-203.

40. Brichta, D.M., et al., *Pseudomonas aeruginosa dihydroorotases: a tale of three pyrCs.* Arch Microbiol, 2004. **182**(1): p. 7-17.

41. Schurr, M.J., et al., *Aspartate transcarbamoylase genes of Pseudomonas putida: requirement for an inactive dihydroorotase for assembly into the dodecameric holoenzyme.* J Bacteriol, 1995. **177**(7): p. 1751-9.

42. Tame, J.R., et al., *The crystal structure of HpcE, a bifunctional decarboxylase/isomerase with a multifunctional fold.* Biochemistry, 2002. **41**(9): p. 2982-9.

43. Klem, T.J., Y. Chen, and V.J. Davisson, *Subunit interactions and glutamine utilization by Escherichia coli imidazole glycerol phosphate synthase.* J Bacteriol, 2001. **183**(3): p. 989-96.

44. Burns, G., et al., *Comparison of the amino acid sequences of the transacylase components of branched chain oxoacid dehydrogenase of Pseudomonas putida, and the pyruvate and 2-oxoglutarate dehydrogenases of Escherichia coli.* Eur J Biochem, 1988. **176**(1): p. 165-9.

45. Sykes, P.J., et al., *Molecular cloning of genes encoding branched-chain keto acid dehydrogenase of Pseudomonas putida.* J Bacteriol, 1987. **169**(4): p. 1619-25.

46. de Kievit, T.R. and J.S. Lam, *Isolation and characterization of two genes, waaC (rfaC) and waaF (rfaF), involved in Pseudomonas aeruginosa serotype O5 inner-core biosynthesis.* J Bacteriol, 1997. **179**(11): p. 3451-7.

47. Yethon, J.A., et al., *Mutation of the lipopolysaccharide core glycosyltransferase encoded by waaG destabilizes the outer membrane of Escherichia coli by interfering with core phosphorylation.* J Bacteriol, 2000. **182**(19): p. 5620-3.

48. Falk-Krzesinski, H.J. and A.J. Wolfe, *Genetic analysis of the nuo locus, which encodes the proton-translocating NADH dehydrogenase in Escherichia coli.* J Bacteriol, 1998. **180**(5): p. 1174-84.

49. Camacho Carvajal, M.M., et al., *Characterization of NADH dehydrogenases of Pseudomonas fluorescens WCS365 and their role in competitive root colonization.* Mol Plant Microbe Interact, 2002. **15**(7): p. 662-71.

50. Alaminos, M. and J.L. Ramos, *The methionine biosynthetic pathway from homoserine in Pseudomonas putida involves the metW, metX, metZ, metH and metE gene products.* Arch Microbiol, 2001. **176**(1-2): p. 151-4.

51. Thoden, J.B., et al., *Molecular structure of Escherichia coli PurT-encoded glycinamide ribonucleotide transformylase.* Biochemistry, 2000. **39**(30): p. 8791-802.

52. Chohnan, S., et al., *Cloning and characterization of mdc genes encoding malonate decarboxylase from Pseudomonas putida.* FEMS Microbiol Lett, 1999. **174**(2): p. 311-9.

53. Kim, Y.S., *Malonate metabolism: biochemistry, molecular biology, physiology, and industrial application.* J Biochem Mol Biol, 2002. **35**(5): p. 443-51.

54. Wong, T.Y. and X.T. Yao, *The DeLey-Doudoroff Pathway of Galactose Metabolism in Azotobacter vinelandii.* Appl Environ Microbiol, 1994. **60**(6): p. 2065-2068.

55. Pettinari, M.J., et al., *Poly(3-hydroxybutyrate) synthesis genes in Azotobacter sp. strain FA8.* Appl Environ Microbiol, 2001. **67**(11): p. 5331-4.

56. Segura, D., E. Vargas, and G. Espin, *Beta-ketothiolase genes in Azotobacter vinelandii.* Gene, 2000. **260**(1-2): p. 113-20.

57. Aiba, H., et al., *The complete nucleotide sequence of the adenylate cyclase gene of Escherichia coli.* Nucleic Acids Res, 1984. **12**(24): p. 9427-40.

58. Debussche, L., et al., *Biosynthesis of the corrin macrocycle of coenzyme B12 in Pseudomonas denitrificans.* J Bacteriol, 1993. **175**(22): p. 7430-40.

59. Raux, E., et al., *Salmonella typhimurium cobalamin (vitamin B12) biosynthetic genes: functional studies in S. typhimurium and Escherichia coli.* J Bacteriol, 1996. **178**(3): p. 753-67.

60. Scott, A.I. and C.A. Roessner, *Biosynthesis of cobalamin (vitamin B(12)).* Biochem Soc Trans, 2002. **30**(4): p. 613-20.

61. Rodionov, D.A., et al., *Comparative genomics of the vitamin B12 metabolism and regulation in prokaryotes.* J Biol Chem, 2003. **278**(42): p. 41148-59.

62. Warren, M.J., et al., *The biosynthesis of adenosylcobalamin (vitamin B12).* Nat Prod Rep, 2002. **19**(4): p. 390-412.

63. Taga, M.E., et al., *BluB cannibalizes flavin to form the lower ligand of vitamin B12.* Nature, 2007. **446**(7134): p. 449-53.

64. Gray, M.J. and J.C. Escalante-Semerena, *Single-enzyme conversion of FMNH2 to 5,6-dimethylbenzimidazole, the lower ligand of B12.* Proceedings of the National Academy of Sciences, 2007. **104**(8): p. 2921-2926.

65. Xie, G., C.A. Bonner, and R.A. Jensen, *A probable mixed-function supraoperon in Pseudomonas exhibits gene organization features of both intergenomic conservation and gene shuffling.* J Mol Evol, 1999. **49**(1): p. 108-21.

66. Xie, G., C.A. Bonner, and R.A. Jensen, *Cyclohexadienyl dehydrogenase from Pseudomonas stutzeri exemplifies a widespread type of tyrosine-pathway dehydrogenase in the TyrA protein family.* Comp Biochem Physiol C Toxicol Pharmacol, 2000. **125**(1): p. 65-83.

67. Calhoun, D.H., et al., *The emerging periplasm-localized subclass of AroQ chorismate mutases, exemplified by those from Salmonella typhimurium and Pseudomonas aeruginosa.* Genome Biol, 2001. **2**(8): p. RESEARCH0030.

68. Kao, C.H. and W.H. Hsu, *A gene cluster involved in pyrimidine reductive catabolism from Brevibacillus agri NCHU1002.* Biochem Biophys Res Commun, 2003. **303**(3): p. 848-54.

69. Yamada, M., et al., *Characterization of the gcd gene from Escherichia coli K-12 W3110 and regulation of its expression.* J Bacteriol, 1993. **175**(2): p. 568-71.

70. Chiurazzi, M., et al., *The Rhizobium leguminosarum biovar phaseoli glnT gene, encoding glutamine synthetase III.* Gene, 1992. **119**(1): p. 1-8.

71. Nishiyama, M., et al., *Cloning and characterization of genes responsible for metabolism of nitrile compounds from Pseudomonas chlororaphis B23.* J Bacteriol, 1991. **173**(8): p. 2465-72.

72. D'Ari, L. and J.C. Rabinowitz, *Purification, characterization, cloning, and amino acid sequence of the bifunctional enzyme 5,10-methylenetetrahydrofolate dehydrogenase/5,10-methenyltetrahydrofolate cyclohydrolase from Escherichia coli.* J Biol Chem, 1991. **266**(35): p. 23953-8.

73. Zhao, G., et al., *Pseudomonas aeruginosa possesses homologues of mammalian phenylalanine hydroxylase and 4 alpha-carbinolamine dehydratase/DCoH as part of a three-component gene cluster.* Proc Natl Acad Sci U S A, 1994. **91**(4): p. 1366-70.

74. Gu, W., et al., *PhhC is an essential aminotransferase for aromatic amino acid catabolism in Pseudomonas aeruginosa.* Microbiology, 1998. **144 ( Pt 11)**: p. 3127-34.

75. Kasai, S. and T. Sumimoto, *Stimulated biosynthesis of flavins in Photobacterium phosphoreum IFO 13896 and the presence of complete rib operons in two species of luminous bacteria.* Eur J Biochem, 2002. **269**(23): p. 5851-60.

76. Hyland, S.A., S.S. Eveland, and M.S. Anderson, *Cloning, expression, and purification of UDP-3-O-acyl-GlcNAc deacetylase from Pseudomonas aeruginosa: a metalloamidase of the lipid A biosynthesis pathway.* J Bacteriol, 1997. **179**(6): p. 2029-37.

77. Lee, P.T., et al., *A C-methyltransferase involved in both ubiquinone and menaquinone biosynthesis: isolation and identification of the Escherichia coli ubiE gene.* J Bacteriol, 1997. **179**(5): p. 1748-54.

78. Kriek, M., et al., *Thiazole synthase from Escherichia coli: an investigation of the substrates and purified proteins required for activity in vitro.* J Biol Chem, 2007. **282**(24): p. 17413-23.

79. Leonardi, R. and P.L. Roach, *Thiamine biosynthesis in Escherichia coli: in vitro reconstitution of the thiazole synthase activity.* J Biol Chem, 2004. **279**(17): p. 17054-62.

80. Yang, X., et al., *The quinone-binding site in succinate-ubiquinone reductase from Escherichia coli. Quinone-binding domain and amino acid residues involved in quinone binding.* J Biol Chem, 1998. **273**(48): p. 31916-23.

81. Marek-Kozaczuk, M., J. Rogalski, and A. Skorupska, *The nadA gene of Pseudomonas fluorescens PGPR strain 267.1.* Curr Microbiol, 2005. **51**(2): p. 122-6.

82. Ceciliani, F., et al., *Cloning, overexpression, and purification of Escherichia coli quinolinate synthetase.* Protein Expr Purif, 2000. **18**(1): p. 64-70.

83. Laber, B., et al., *Vitamin B6 biosynthesis: formation of pyridoxine 5'-phosphate from 4-(phosphohydroxy)-L-threonine and 1-deoxy-D-xylulose-5-phosphate by PdxA and PdxJ protein.* FEBS Lett, 1999. **449**(1): p. 45-8.

84. Poon, W.W., et al., *Identification of Escherichia coli ubiB, a gene required for the first monooxygenase step in ubiquinone biosynthesis.* J Bacteriol, 2000. **182**(18): p. 5139-46.

85. Siebert, M., K. Severin, and L. Heide, *Formation of 4-hydroxybenzoate in Escherichia coli: characterization of the ubiC gene and its encoded enzyme chorismate pyruvate-lyase.* Microbiology, 1994. **140 ( Pt 4)**: p. 897-904.

86. Strickland, S. and V. Massey, *The purification and properties of the flavoprotein melilotate hydroxylase.* J Biol Chem, 1973. **248**(8): p. 2944-52.

87. Ferrandez, A., J.L. Garcia, and E. Diaz, *Genetic characterization and expression in heterologous hosts of the 3-(3-hydroxyphenyl)propionate catabolic pathway of Escherichia coli K-12.* J Bacteriol, 1997. **179**(8): p. 2573-81.

88. Prieto, M.A. and J.L. Garcia, *Identification of a novel positive regulator of the 4-hydroxyphenylacetate catabolic pathway of Escherichia coli.* Biochem Biophys Res Commun, 1997. **232**(3): p. 759-65.

89. Tropel, D. and J.R. van der Meer, *Bacterial transcriptional regulators for degradation pathways of aromatic compounds.* Microbiol Mol Biol Rev, 2004. **68**(3): p. 474-500.

90. Au, K.G., et al., *Escherichia coli mutY gene encodes an adenine glycosylase active on G-A mispairs.* Proc Natl Acad Sci U S A, 1989. **86**(22): p. 8877-81.

91. Michaels, M.L. and J.H. Miller, *The GO system protects organisms from the mutagenic effect of the spontaneous lesion 8-hydroxyguanine (7,8-dihydro-8-oxoguanine).* J Bacteriol, 1992. **174**(20): p. 6321-5.

92. Oliver, A., J.M. Sanchez, and J. Blazquez, *Characterization of the GO system of Pseudomonas aeruginosa.* FEMS Microbiol Lett, 2002. **217**(1): p. 31-5.

93. Hildmann, C., et al., *A new amidohydrolase from Bordetella or Alcaligenes strain FB188 with similarities to histone deacetylases.* J Bacteriol, 2004. **186**(8): p. 2328-39.

94. Sakurada, K., et al., *Acetylpolyamine amidohydrolase from Mycoplana ramosa: gene cloning and characterization of the metal-substituted enzyme.* J Bacteriol, 1996. **178**(19): p. 5781-6.

95. Magnusson, O.T., et al., *Quinone biogenesis: Structure and mechanism of PqqC, the final catalyst in the production of pyrroloquinoline quinone.* Proc Natl Acad Sci U S A, 2004. **101**(21): p. 7913-8.

96. Toyama, H., et al., *Factors required for the catalytic reaction of PqqC/D which produces pyrroloquinoline quinone.* Biochem Biophys Res Commun, 2007. **354**(1): p. 290-5.

97. Robichon, C., D. Vidal-Ingigliardi, and A.P. Pugsley, *Depletion of apolipoprotein N-acyltransferase causes mislocalization of outer membrane lipoproteins in Escherichia coli.* J Biol Chem, 2005. **280**(2): p. 974-83.

98. Desai, P.M. and J.P. Rife, *The adenosine dimethyltransferase KsgA recognizes a specific conformational state of the 30S ribosomal subunit.* Arch Biochem Biophys, 2006. **449**(1-2): p. 57-63.

99. Kim, Y.S., et al., *Cloning of Pseudomonas fluorescens carboxylesterase gene and characterization of its product expressed in Escherichia coli.* Biosci Biotechnol Biochem, 1994. **58**(1): p. 111-6.

100. Suzuki, H., et al., *DNA sequence of the Escherichia coli K-12 gamma-glutamyltranspeptidase gene, ggt.* J Bacteriol, 1989. **171**(9): p. 5169-72.

101. Wegele, R., et al., *The heme oxygenase(s)-phytochrome system of Pseudomonas aeruginosa.* J Biol Chem, 2004. **279**(44): p. 45791-802.

102. Abramochkin, G. and T.E. Shrader, *The leucyl/phenylalanyl-tRNA-protein transferase. Overexpression and characterization of substrate recognition, domain structure, and secondary structure.* J Biol Chem, 1995. **270**(35): p. 20621-8.

103. Izquierdo, L., et al., *The wavB gene of Vibrio cholerae and the waaE of Klebsiella pneumoniae codify for a beta-1,4-glucosyltransferase involved in the transfer of a glucose residue to the L-glycero-D-manno-heptose I in the lipopolysaccharide inner core.* FEMS Microbiol Lett, 2002. **216**(2): p. 211-6.

104. Regue, M., et al., *Genetic characterization of the Klebsiella pneumoniae waa gene cluster, involved in core lipopolysaccharide biosynthesis.* J Bacteriol, 2001. **183**(12): p. 3564-73.

105. Miller, J.R., et al., *Escherichia coli LipA is a lipoyl synthase: in vitro biosynthesis of lipoylated pyruvate dehydrogenase complex from octanoyl-acyl carrier protein.* Biochemistry, 2000. **39**(49): p. 15166-78.

106. Jordan, S.W. and J.E. Cronan, Jr., *The Escherichia coli lipB gene encodes lipoyl (octanoyl)-acyl carrier protein:protein transferase.* J Bacteriol, 2003. **185**(5): p. 1582-9.

107. Zhao, X., et al., *Assembly of the covalent linkage between lipoic acid and its cognate enzymes.* Chem Biol, 2003. **10**(12): p. 1293-302.

108. Cudny, H., et al., *Cloning, sequencing, and species relatedness of the Escherichia coli cca gene encoding the enzyme tRNA nucleotidyltransferase.* J Biol Chem, 1986. **261**(14): p. 6444-9.

109. Shi, P.Y., N. Maizels, and A.M. Weiner, *CCA addition by tRNA nucleotidyltransferase: polymerization without translocation?* Embo J, 1998. **17**(11): p. 3197-206.

110. Langaee, T.Y., M. Dargis, and A. Huletsky, *An ampD gene in Pseudomonas aeruginosa encodes a negative regulator of AmpC beta-lactamase expression.* Antimicrob Agents Chemother, 1998. **42**(12): p. 3296-300.

111. Nunez, C., et al., *Inactivation of the ampDE operon increases transcription of algD and affects morphology and encystment of Azotobacter vinelandii.* J Bacteriol, 2000. **182**(17): p. 4829-35.

112. Miura, K., et al., *Molecular cloning of the nemA gene encoding N-ethylmaleimide reductase from Escherichia coli.* Biol Pharm Bull, 1997. **20**(1): p. 110-2.

113. Dean, C.R., et al., *Characterization of the serogroup O11 O-antigen locus of Pseudomonas aeruginosa PA103.* J Bacteriol, 1999. **181**(14): p. 4275-84.

114. Mulrooney, E.F., et al., *Biosynthesis of UDP-N-acetyl-L-fucosamine, a precursor to the biosynthesis of lipopolysaccharide in Pseudomonas aeruginosa serotype O11.* J Biol Chem, 2005. **280**(20): p. 19535-42.

115. Pfluger, K. and V. de Lorenzo, *Growth-dependent Phosphorylation of the PtsN (EIINtr) Protein of Pseudomonas putida.* J Biol Chem, 2007. **282**(25): p. 18206-11.

116. Solaiman, D.K. and R.D. Ashby, *Genetic characterization of the poly(hydroxyalkanoate) synthases of various Pseudomonas oleovorans strains.* Curr Microbiol, 2005. **50**(6): p. 329-33.

117. Stevenson, G., et al., *Organization of the Escherichia coli K-12 gene cluster responsible for production of the extracellular polysaccharide colanic acid.* J Bacteriol, 1996. **178**(16): p. 4885-93.

118. Whitfield, C., *Biosynthesis and assembly of capsular polysaccharides in Escherichia coli.* Annu Rev Biochem, 2006. **75**: p. 39-68.

119. Streit, W.R. and P. Entcheva, *Biotin in microbes, the genes involved in its biosynthesis, its biochemical role and perspectives for biotechnological production.* Appl Microbiol Biotechnol, 2003. **61**(1): p. 21-31.

120. Sanishvili, R., et al., *Integrating structure, bioinformatics, and enzymology to discover function: BioH, a new carboxylesterase from Escherichia coli.* J Biol Chem, 2003. **278**(28): p. 26039-45.

121. Ifuku, O., et al., *Origin of carbon atoms of biotin. 13C-NMR studies on biotin biosynthesis in Escherichia coli.* Eur J Biochem, 1994. **220**(2): p. 585-91.

122. Zhang, X.X., et al., *The histidine utilization (hut) genes of Pseudomonas fluorescens SBW25 are active on plant surfaces, but are not required for competitive colonization of sugar beet seedlings.* Microbiology, 2006. **152**(Pt 6): p. 1867-75.

123. McMorran, B.J., et al., *Involvement of a transformylase enzyme in siderophore synthesis in Pseudomonas aeruginosa.* Microbiology, 2001. **147**(Pt 6): p. 1517-24.

124. Bishop, A.C., et al., *Identification of the tRNA-dihydrouridine synthase family.* J Biol Chem, 2002. **277**(28): p. 25090-5.

125. Persson, B.C., et al., *The ms2io6A37 modification of tRNA in Salmonella typhimurium regulates growth on citric acid cycle intermediates.* J Bacteriol, 1998. **180**(12): p. 3144-51.

126. Carpinelli, J., R. Kramer, and E. Agosin, *Metabolic engineering of Corynebacterium glutamicum for trehalose overproduction: role of the TreYZ trehalose biosynthetic pathway.* Appl Environ Microbiol, 2006. **72**(3): p. 1949-55.

127. Cronan, J.E., Jr. and G.L. Waldrop, *Multi-subunit acetyl-CoA carboxylases.* Prog Lipid Res, 2002. **41**(5): p. 407-35.

128. Kahnert, A., et al., *The ssu locus plays a key role in organosulfur metabolism in Pseudomonas putida S-313.* J Bacteriol, 2000. **182**(10): p. 2869-78.

129. Kertesz, M.A., K. Schmidt-Larbig, and T. Wuest, *A novel reduced flavin mononucleotide-dependent methanesulfonate sulfonatase encoded by the sulfur-regulated msu operon of Pseudomonas aeruginosa.* J Bacteriol, 1999. **181**(5): p. 1464-73.

130. Salazar, J.C., et al., *A dual-specific Glu-tRNA(Gln) and Asp-tRNA(Asn) amidotransferase is involved in decoding glutamine and asparagine codons in Acidithiobacillus ferrooxidans.* FEBS Lett, 2001. **500**(3): p. 129-31.

131. Kapatral, V., X. Bina, and A.M. Chakrabarty, *Succinyl coenzyme A synthetase of Pseudomonas aeruginosa with a broad specificity for nucleoside triphosphate (NTP) synthesis modulates specificity for NTP synthesis by the 12-kilodalton form of nucleoside diphosphate kinase.* J Bacteriol, 2000. **182**(5): p. 1333-9.

132. Darrouzet, E., et al., *Structure and function of the bacterial bc1 complex: domain movement, subunit interactions, and emerging rationale engineering attempts.* J Bioenerg Biomembr, 1999. **31**(3): p. 275-88.

133. Neidle, E.L., et al., *Nucleotide sequences of the Acinetobacter calcoaceticus benABC genes for benzoate 1,2-dioxygenase reveal evolutionary relationships among multicomponent oxygenases.* J Bacteriol, 1991. **173**(17): p. 5385-95.

134. Torrents, E., et al., *Ribonucleotide reductase modularity: Atypical duplication of the ATP-cone domain in Pseudomonas aeruginosa.* J Biol Chem, 2006. **281**(35): p. 25287-96.

135. van den Heuvel, R.H., et al., *Glutamate synthase: a fascinating pathway from L-glutamine to L-glutamate.* Cell Mol Life Sci, 2004. **61**(6): p. 669-81.

136. Fjellstrom, O., et al., *Catalytic properties of hybrid complexes of the NAD(H)-binding and NADP(H)-binding domains of the proton-translocating transhydrogenases from Escherichia coli and Rhodospirillum rubrum.* Biochemistry, 1999. **38**(1): p. 415-22.

137. Das, A. and L.G. Ljungdahl, *Clostridium pasteurianum F1Fo ATP synthase: operon, composition, and some properties.* J Bacteriol, 2003. **185**(18): p. 5527-35.

138. Hicks, D.B., et al., *A tenth atp gene and the conserved atpI gene of a Bacillus atp operon have a role in Mg2+ uptake.* Proc Natl Acad Sci U S A, 2003. **100**(18): p. 10213-8.

139. Koper, T.E., et al., *Urease-encoding genes in ammonia-oxidizing bacteria.* Appl Environ Microbiol, 2004. **70**(4): p. 2342-8.

140. Itoh, Y., *Cloning and characterization of the aru genes encoding enzymes of the catabolic arginine succinyltransferase pathway in Pseudomonas aeruginosa.* J Bacteriol, 1997. **179**(23): p. 7280-90.

141. Martin, M.C., et al., *Sequencing, characterization and transcriptional analysis of the histidine decarboxylase operon of Lactobacillus buchneri.* Microbiology, 2005. **151**(Pt 4): p. 1219-28.

142. Stols, L., et al., *New vectors for co-expression of proteins: structure of Bacillus subtilis ScoAB obtained by high-throughput protocols.* Protein Expr Purif, 2007. **53**(2): p. 396-403.

143. Barbe, V., et al., *Unique features revealed by the genome sequence of Acinetobacter sp. ADP1, a versatile and naturally transformation competent bacterium.* Nucleic Acids Res, 2004. **32**(19): p. 5766-79.

144. Self, C.H. and P.D. Weitzman, *The isocitrate dehydrogenases of Acinetobacter woffi. Separation and properties of two nicotinamide-adenine dinucleotide phosphate-linked isoenzymes.* Biochem J, 1972. **130**(1): p. 211-9.

145. Banerjee, S., et al., *Comparison of Mycobacterium tuberculosis isocitrate dehydrogenases (ICD-1 and ICD-2) reveals differences in coenzyme affinity, oligomeric state, pH tolerance and phylogenetic affiliation.* BMC Biochem, 2005. **6**: p. 20.

146. Zawadzke, L.E., T.D. Bugg, and C.T. Walsh, *Existence of two D-alanine:D-alanine ligases in Escherichia coli: cloning and sequencing of the ddlA gene and purification and characterization of the DdlA and DdlB enzymes.* Biochemistry, 1991. **30**(6): p. 1673-82.

147. Kurihara, S., et al., *A novel putrescine utilization pathway involves gamma-glutamylated intermediates of Escherichia coli K-12.* J Biol Chem, 2005. **280**(6): p. 4602-8.

148. Rexer, H.U., et al., *Investigation of the functional properties and regulation of three glutamine synthetase-like genes in Streptomyces coelicolor A3(2).* Arch Microbiol, 2006. **186**(6): p. 447-58.

149. Szymanski, M., M. Deniziak, and J. Barciszewski, *The new aspects of aminoacyl-tRNA synthetases.* Acta Biochim Pol, 2000. **47**(3): p. 821-34.

150. Hoang, T.T., et al., *Molecular genetic analysis of the region containing the essential Pseudomonas aeruginosa asd gene encoding aspartate-beta-semialdehyde dehydrogenase.* Microbiology, 1997. **143 ( Pt 3)**: p. 899-907.

151. Cheng, G., et al., *Allophanate hydrolase, not urease, functions in bacterial cyanuric acid metabolism.* Appl Environ Microbiol, 2005. **71**(8): p. 4437-45.

152. Mortl, S., et al., *Biosynthesis of riboflavin. Lumazine synthase of Escherichia coli.* J Biol Chem, 1996. **271**(52): p. 33201-7.

153. Hechtman, P., C.R. Scriver, and R.B. Middleton, *Isolation and Properties of a {beta}-Alanine Transaminaseless Mutant of Pseudomonas fluorescens.* J. Bacteriol., 1970. **104**(2): p. 851-856.

154. Ha, J.Y., et al., *Crystal Structure of 2-Nitropropane Dioxygenase Complexed with FMN and Substrate: IDENTIFICATION OF THE CATALYTIC BASE.* J. Biol. Chem., 2006. **281**(27): p. 18660-18667.

155. Fuhrer, T., et al., *Computational Prediction and Experimental Verification of the Gene Encoding the NAD+/NADP+-Dependent Succinate Semialdehyde Dehydrogenase in Escherichia coli.* J. Bacteriol., 2007. **189**(22): p. 8073-8078.

156. Lu, C.-D. and A.T. Abdelal, *The gdhB Gene of Pseudomonas aeruginosa Encodes an Arginine-Inducible NAD+-Dependent Glutamate Dehydrogenase Which Is Subject to Allosteric Regulation.* J. Bacteriol., 2001. **183**(2): p. 490-499.

157. Ishikawa, M., et al., *Reconstitution, morphology and crystallization of a fatty acid beta-oxidation multienzyme complex from Pseudomonas fragi.* Biochem. J., 1997. **328**(3): p. 815-820.

158. Taylor, P.L., et al., *Structure and Function of Sedoheptulose-7-phosphate Isomerase, a Critical Enzyme for Lipopolysaccharide Biosynthesis and a Target for Antibiotic Adjuvants.* J. Biol. Chem., 2008. **283**(5): p. 2835-2845.

159. Stephane Raymond, A.T.E.A.Y.L.M.-N.H.A.M.M.C., *Crystal structure of ureidoglycolate hydrolase (AllA) from <I>Escherichia coli</I> O157:H7.* Proteins: Structure, Function, and Bioinformatics, 2005. **61**(2): p. 454-459.

160. Ramos, J.L., *Pseudomonas: Biosynthesis of macromolecules and molecular metabolism*. 2004: Kluwer Academic/Plenum.

161. Sigeru Chohnan, Y.K.H.N.Y.T., *Cloning and characterization of <i>mdc</i> genes encoding malonate decarboxylase from <i>Pseudomonas putida</i>.* FEMS Microbiology Letters, 1999. **174**(2): p. 311-319.

162. Schneider, K., P. Dimroth, and M. Bott, *Biosynthesis of the Prosthetic Group of Citrate Lyase&#x2020.* Biochemistry, 2000. **39**(31): p. 9438-9450.

163. Hoenke, S., M.R. Wild, and P. Dimroth, *Biosynthesis of Triphosphoribosyl-dephospho-coenzyme A, the Precursor of the Prosthetic Group of Malonate Decarboxylase&#x2020.* Biochemistry, 2000. **39**(43): p. 13223-13232.

164. José Ignacio Jiménez, B.M.J.L.G.E.D., *Genomic analysis of the aromatic catabolic pathways from <i>Pseudomonas putida</i> KT2440.* Environmental Microbiology, 2002. **4**(12): p. 824-841.

165. Vilchez, S., et al., *Proline Catabolism by Pseudomonas putida: Cloning, Characterization, and Expression of the put Genes in the Presence of Root Exudates.* J. Bacteriol., 2000. **182**(1): p. 91-99.

166. Lawrence, A.D., et al., *Identification, Characterization, and Structure/Function Analysis of a Corrin Reductase Involved in Adenosylcobalamin Biosynthesis.* J. Biol. Chem., 2008. **283**(16): p. 10813-10821.

167. Brand, L.A. and E. Strauss, *Characterization of a New Pantothenate Kinase Isoform from Helicobacter pylori.* J. Biol. Chem., 2005. **280**(21): p. 20185-20188.

168. Rea, D., et al., *Structure and Mechanism of HpcH: A Metal Ion Dependent Class II Aldolase from the Homoprotocatechuate Degradation Pathway of Escherichia coli.* Journal of Molecular Biology, 2007. **373**(4): p. 866-876.

169. Hoang, T.T. and H.P. Schweizer, *Fatty acid biosynthesis in Pseudomonas aeruginosa: cloning and characterization of the fabAB operon encoding beta-hydroxyacyl-acyl carrier protein dehydratase (FabA) and beta-ketoacyl-acyl carrier protein synthase I (FabB).* J. Bacteriol., 1997. **179**(17): p. 5326-5332.

170. Gonzalez, C.F., et al., *Molecular Basis of Formaldehyde Detoxification: CHARACTERIZATION OF TWO S-FORMYLGLUTATHIONE HYDROLASES FROM ESCHERICHIA COLI, FrmB AND YeiG.* J. Biol. Chem., 2006. **281**(20): p. 14514-14522.

171. Pfluger, K. and V. de Lorenzo, *Evidence of In Vivo Cross Talk between the Nitrogen-Related and Fructose-Related Branches of the Carbohydrate Phosphotransferase System of Pseudomonas putida.* J. Bacteriol., 2008. **190**(9): p. 3374-3380.

172. Minna Mäki, N.J.J.R.C.R.H.M.R.R., *Functional expression of <i>Pseudomonas aeruginosa</i> GDP-4-keto-6-deoxy-d-mannose reductase which synthesizes GDP-rhamnose.* European Journal of Biochemistry, 2002. **269**(2): p. 593-601.

173. Bologna, F.P., C.S. Andreo, and M.F. Drincovich, *Escherichia coli Malic Enzymes: Two Isoforms with Substantial Differences in Kinetic Properties, Metabolic Regulation, and Structure.* J. Bacteriol., 2007. **189**(16): p. 5937-5946.

174. Goward, C.R. and D.J. Nicholls, *Malate dehydrogenase: A model for structure, evolution, and catalysis.* Protein Sci, 1994. **3**(10): p. 1883-1888.

175. Singh, R., et al., *A novel strategy involved anti-oxidative defense: the conversion of NADH into NADPH by a metabolic network.* PLoS ONE, 2008. **3**(7): p. e2682.

176. Yum, D.Y., Y.P. Lee, and J.G. Pan, *Cloning and expression of a gene cluster encoding three subunits of membrane-bound gluconate dehydrogenase from Erwinia cypripedii ATCC 29267 in Escherichia coli.* J. Bacteriol., 1997. **179**(21): p. 6566-6572.

177. Britta L. Swanson, P.H.P.P.J.U.O.M.L.V.A.N.H., *Characterization of the 2-ketogluconate utilization operon in <i>Pseudomonas aeruginosa</i> PAO1.* Molecular Microbiology, 2000. **37**(3): p. 561-573.

178. Wargo, M.J., B.S. Szwergold, and D.A. Hogan, *Identification of Two Gene Clusters and a Transcriptional Regulator Required for Pseudomonas aeruginosa Glycine Betaine Catabolism.* J. Bacteriol., 2008. **190**(8): p. 2690-2699.

179. Dauvillee, D., et al., *Role of the Escherichia coli glgX Gene in Glycogen Metabolism.* J. Bacteriol., 2005. **187**(4): p. 1465-1473.

180. Clementz, T. and C.R. Raetz, *A gene coding for 3-deoxy-D-manno-octulosonic-acid transferase in Escherichia coli. Identification, mapping, cloning, and sequencing.* J. Biol. Chem., 1991. **266**(15): p. 9687-9696.

181. Mohan, S. and C.R. Raetz, *Endotoxin biosynthesis in Pseudomonas aeruginosa: enzymatic incorporation of laurate before 3-deoxy-D-manno-octulosonate.* J. Bacteriol., 1994. **176**(22): p. 6944-6951.

182. Yum, D.-Y., B.-Y. Lee, and J.-G. Pan, *Identification of the yqhE and yafB Genes Encoding Two 2,5-Diketo-D-Gluconate Reductases in Escherichia coli.* Appl. Environ. Microbiol., 1999. **65**(8): p. 3341-3346.

183. Bausch, C., M. Ramsey, and T. Conway, *Transcriptional Organization and Regulation of the L-Idonic Acid Pathway (GntII System) in Escherichia coli.* J. Bacteriol., 2004. **186**(5): p. 1388-1397.

184. Schneider, B.L., A.K. Kiupakis, and L.J. Reitzer, *Arginine Catabolism and the Arginine Succinyltransferase Pathway in Escherichia coli.* J. Bacteriol., 1998. **180**(16): p. 4278-4286.

185. Bramer, C.O. and A. Steinbuchel, *The methylcitric acid pathway in Ralstonia eutropha: new genes identified involved in propionate metabolism.* Microbiology, 2001. **147**(8): p. 2203-2214.

186. Azzolina, B.A., et al., *The Cell Wall and Cell Division Gene Cluster in the Mra Operon of Pseudomonas aeruginosa: Cloning, Production, and Purification of Active Enzymes.* Protein Expression and Purification, 2001. **21**(3): p. 393-400.

187. Keasling, J.D., L. Bertsch, and A. Kornberg, *Guanosine pentaphosphate phosphohydrolase of Escherichia coli is a long-chain exopolyphosphatase.* Proceedings of the National Academy of Sciences of the United States of America, 1993. **90**(15): p. 7029-7033.

188. Proudfoot, M., et al., *General Enzymatic Screens Identify Three New Nucleotidases in Escherichia coli: BIOCHEMICAL CHARACTERIZATION OF SurE, YfbR, AND YjjG.* J. Biol. Chem., 2004. **279**(52): p. 54687-54694.

189. Zhang, Y., M. Morar, and S.E. Ealick, *Structural biology of the purine biosynthetic pathway.* Cell Mol Life Sci, 2008. **65**(23): p. 3699-724.

190. Jordan, A., et al., *Ribonucleotide Reduction in Pseudomonas Species: Simultaneous Presence of Active Enzymes from Different Classes.* J. Bacteriol., 1999. **181**(13): p. 3974-3980.

191. Meyer, E., et al., *Evidence for the Direct Transfer of the Carboxylate of N5-Carboxyaminoimidazole Ribonucleotide (N5-CAIR) To Generate 4-Carboxy-5-aminoimidazole Ribonucleotide Catalyzed by Escherichia coli PurE, an N5-CAIR Mutase&#x2020.* Biochemistry, 1999. **38**(10): p. 3012-3018.

192. Tiedeman, A.A., J.M. Smith, and H. Zalkin, *Nucleotide sequence of the guaA gene encoding GMP synthetase of Escherichia coli K12.* J. Biol. Chem., 1985. **260**(15): p. 8676-8679.

193. Kouzuma, A., et al., *Transcription Factors CysB and SfnR Constitute the Hierarchical Regulatory System for the Sulfate Starvation Response in Pseudomonas putida.* J. Bacteriol., 2008. **190**(13): p. 4521-4531.

194. Rodionov, D.A., et al., *Comparative Genomics of Thiamin Biosynthesis in Procaryotes. NEW GENES AND REGULATORY MECHANISMS.* J. Biol. Chem., 2002. **277**(50): p. 48949-48959.

195. Amemura, A., et al., *Cloning and nucleotide sequence of the isoamylase gene from Pseudomonas amyloderamosa SB-15.* J. Biol. Chem., 1988. **263**(19): p. 9271-9275.

196. Matthijs, S., et al., *The trehalose operon of Pseudomonas fluorescens ATCC 17400.* Research in Microbiology, 2000. **151**(10): p. 845-851.

197. Lim, W.K., S.K. Sarkar, and J.K. Hardman, *Enzymatic properties of mutant Escherichia coli tryptophan synthase alpha-subunits.* J. Biol. Chem., 1991. **266**(30): p. 20205-20212.

198. Meganathan, R., *Ubiquinone biosynthesis in microorganisms.* FEMS Microbiology Letters, 2001. **203**(2): p. 131-139.

199. Valiente, E., et al., *Vibrio vulnificus Biotype 2 Serovar E gne but Not galE Is Essential for Lipopolysaccharide Biosynthesis and Virulence.* Infect. Immun., 2008. **76**(4): p. 1628-1638.

200. Creuzenet, C., et al., *Expression, Purification, and Biochemical Characterization of WbpP, a New UDP-GlcNAc C4 Epimerase from Pseudomonas aeruginosa Serotype O6.* J. Biol. Chem., 2000. **275**(25): p. 19060-19067.

201. Ishiyama, N., et al., *Crystal Structure of WbpP, a Genuine UDP-N-acetylglucosamine 4-Epimerase from Pseudomonas aeruginosa: SUBSTRATE SPECIFICITY IN UDP-HEXOSE 4-EPIMERASES.* J. Biol. Chem., 2004. **279**(21): p. 22635-22642.

202. Wong, B.J. and J.A. Gerlt, *Divergent Function in the Crotonase Superfamily:&nbsp; An Anhydride Intermediate in the Reaction Catalyzed by 3-Hydroxyisobutyryl-CoA Hydrolase.* Journal of the American Chemical Society, 2003. **125**(40): p. 12076-12077.

203. Vévodová, J., et al., *Structure/Function Studies on a S-Adenosyl-l-methionine-dependent Uroporphyrinogen III C Methyltransferase (SUMT), a Key Regulatory Enzyme of Tetrapyrrole Biosynthesis.* Journal of Molecular Biology, 2004. **344**(2): p. 419-433.

204. Ramazzina, I., et al., *Logical Identification of an Allantoinase Analog (puuE) Recruited from Polysaccharide Deacetylases.* J. Biol. Chem., 2008. **283**(34): p. 23295-23304.

205. Hu, L. and A.T. Phillips, *Organization and multiple regulation of histidine utilization genes in Pseudomonas putida.* J. Bacteriol., 1988. **170**(9): p. 4272-4279.

206. Hester, K.L., et al., *Crc Is Involved in Catabolite Repression Control of the bkd Operons of Pseudomonas putida and Pseudomonas aeruginosa.* J. Bacteriol., 2000. **182**(4): p. 1144-1149.
